# Halogenation-Guided Chemical Screening Uncovers Cyanobacterin Analogues from the Cyanobacterium *Tolypothrix* sp. PCC9009

**DOI:** 10.1021/acs.jnatprod.5c00591

**Published:** 2025-09-12

**Authors:** Franziska Schanbacher, Arthur Guljamow, Valerie I. C. Rebhahn, Peter Schmieder, Heike Enke, Elke Dittmann, Martin Baunach, Timo H. J. Niedermeyer

**Affiliations:** † Department of Pharmaceutical Biology, Institute of Pharmacy, 9166 Freie Universität Berlin, 14195 Berlin, Germany; # Department of Pharmaceutical Biology/Pharmacognosy, Institute of Pharmacy, Martin-Luther-University Halle-Wittenberg, 06120 Halle (Saale), Germany; ‡ Institute for Biochemistry and Biology, 26583 University of Potsdam, 14476 Potsdam, Germany; § Leibniz-Forschungsinstitut für Molekulare Pharmakologie, Department of NMR-Supported Structural Biology, 13125 Berlin, Germany; ∥ Simris Biologics GmbH, 12489 Berlin, Germany; ⊥ Institute of Pharmaceutical Biology, University of Bonn, 53115 Bonn, German

## Abstract

Halogenated specialized metabolites show high chemical diversity and exhibit a range of biological activities. A targeted screening of a cyanobacteria extract library for halogenated specialized metabolites using HPLC-HRMS combined with MassQL and Haloseeker indicated that several of the extracts contained halogenated compounds, among them an extract of *Tolypothrix* sp. PCC9009. This freshwater cyanobacterium has been known since the early 1980s for producing the chlorinated specialized metabolite cyanobacterin, containing a γ-lactone core structure with a hydroxy group that is essential for its herbicidal activity against cyanobacteria and green algae. Mass-spectrometry-based molecular networking was employed to explore the chemical space of natural cyanobacterin analogues. This analysis led to the identification of 15 previously unknown compounds structurally related to cyanobacterin, most of which are related to anhydrocyanobacterin, including a dimer formed by [2 + 2] photocycloaddition. Two further analogues, previously reported following heterologous expression of the cyanobacterin biosynthetic gene cluster in *E. coli* as the nonchlorinated precyanobacterin I and II, were now isolated from the natural cyanobacterin producer. Cytotoxicity assays of cyanobacterin, anhydrocyanobacterin and one further isolated analogue showed only modest activity of the compounds against HCT116 cells.

## Graphical abstract

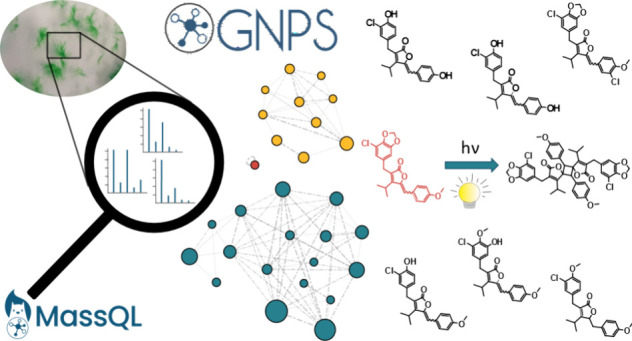

Cyanobacteria are a prolific source of chemically diverse bioactive natural products and known for their production of halogenated specialized metabolites (HSMs).^1–3^ Approximately 8,400 naturally occurring organohalogen compounds are known today.^4^ Evaluation of the cyanobacteria-focused specialized metabolite database CyanoMetDB 3.0 showed that about 8% of the known HSMs are produced by cyanobacteria (about 20% of the 3087 entries).^5,6^ Most of the known cyanobacterial HSMs are chlorinated, and have been isolated from three major genera: *Microcystis*, *Nostoc*, and *Moorena*. In terms of structural diversity, 41% of chlorinated SMs are peptides. Among these, 77% belong to nonribosomal peptides such as the aeruginosins^7^ and cyanopeptolins,^8^ or to hybrid NRPS–PKS compounds such as microginins,^9^ cryptophycins,^10^ and lyngbyabellins.^11–13^ The remaining 59% of chlorinated SMs are distributed among polyketides (10%), alkaloids (13%), fatty acid derivatives (18%), and other core structures (18%). Representative nonpeptide HSMs include the hapalindoles,^14–17^ welwitindolinones,^18^ fischerindoles,^19^ ambiguines,^20^ ambigoles,^21,22^ tjipanazoles,^23,24^ malyngamides,^25^ cyclophanes,^26–28^ bartolosides,^29^ luquilloamides,^30^ beru’amide,^31^ and anaenamides.^32^

Cyanobacterin (CB), a chlorinated SM first isolated from the freshwater cyanobacterium *Tolypothrix* sp., formerly known as *Scytonema hofmanni*, in the early 1980s,^33^ shows activity against other cyanobacteria and green algae, as demonstrated by coculture experiments and bioactivity assays against *Synechococcus* sp.^33–35^ By the mid-1980s, the chemical structure and configuration of CB had been determined, identifying a lactone core structure with a hydroxy group essential for binding to its target and thus its biological activity.^36–38^ The anhydro congener lacking the hydroxy group showed no herbicidal activity.^39^ Similar results were observed with synthetic derivatives lacking this key element.^37,40^ It was also discovered that the halogenation as found in the naturally occurring compound is necessary for biological activity.^37,40^ In addition to its effect on cyanobacteria and green algae, CB has shown activity against some angiosperms, including duckweed, peas, and maize.^41^ Its primary target is the photosystem (PS) II, although it binds at a different site than inhibitors such as 3-(3,4-dichlorophenyl)-1,1-dimethylurea (DCMU).^35,42–44^ This made CB a promising candidate for the development of novel herbicides that can bypass resistance mechanisms against traditional PSII inhibitors. However, no CB-based herbicide has entered the market, yet.

The discovery of novel SMs or the comprehensive exploration of the chemical diversity within known SM families from cyanobacteria is often hindered by their low abundance in the initial material, sometimes making them difficult to access with traditional research methods, and requiring considerable investment of resources to isolate and characterize them. Recent methodological developments, particularly in mass spectrometry enhanced by computational approaches, are accelerating the discovery process of the previously inaccessible chemical space. They also enable the efficient screening of large sample sets,^45^ which is crucial for the analysis of the chemical space covered by cyanobacterial SMs. Accurate tandem mass spectrometry raw data is extremely information-rich, capturing unique features of chemical structures or isotope patterns. It is particularly effective in identifying chlorinated and brominated compounds, due to their distinctive isotope patterns (^35^Cl^/37^Cl and ^79^Br/^81^Br). Tools such as Haloseeker^46^ or MassQL^47^ can be used to filter for (poly-)­halogenated ions in complex HRMS data sets, while Classical Molecular Networking (CMN)^48^ and Feature-Based Molecular Networking (FBMN)^49^ facilitate the rapid suggestion of compound structures without isolation, provided prior knowledge of the structure and biosynthesis of key reference compounds of the same compound family is available.

Our ongoing interest in cyanobacterial HSMs^21,23,50–52^ prompted us to conduct an HRMS-based halogenation-guided screening of a library of 547 cyanobacterial extracts, revealing several extracts containing HSMs that seemed worthwhile for subsequent work. In this manuscript, we describe the isolation, structure elucidation and cytotoxicity of HSMs from one of these strains: analogues of CB produced by *Tolypothrix* sp. PCC9009. Most interesting, we report the discovery of a cyanobacterial cyclobutane-linked dimer, a structural motif previously unreported in cyanobacteria.

## Results and Discussion

### Halogenation-Guided Screening

A library of 363 cyanobacteria biomass and 184 medium extracts was analyzed by HPLC-HRMS^2^ and subsequently evaluated using HaloSeeker,^53^ an open-source postacquisition processing software designed for nontargeted screening for halogenated compounds within HRMS data sets. We also used MassQL,^47^ an SQL (Structured Query Language)-inspired language for finding mass spectrometry data patterns. The screening was done separately for positive and negative ionization mode. For the MassQL query results, signals were grouped as the same compound/feature based on retention time, accurate mass, and matching isotope patterns. This was particularly important when multiple *m*/*z* features of one single SM artificially increased the number of detected features. Adduct formation was also taken into account. Initial evaluation of the MassQL and HaloSeeker outputs quickly showed that isotope pattern–based screening can be affected by false positives. This prompted a more detailed examination of the limitations of isotope pattern recognition, as outlined below.

As demonstrated in several studies, examining mass spectra for isotope patterns - particularly those of bromine and chlorine - remains a valuable strategy when screening for HSMs.^23,46,47,54^ However, upon evaluating the data using our first MassQL query (Figure S1) and Haloseeker, we obtained numerous false positives for higher-molecular weight compounds due to the contribution of ^13^C isotopes to the isotope pattern. While the contribution of ^13^C isotopes is most prominent at the M+1 peak in compounds with a low number of carbon atoms and generally negligible at M+2, their influence becomes increasingly relevant in carbon-rich compounds. We calculated that carbon-rich compounds exceeding 70–75 carbon atoms have a natural isotope pattern (regarding the M+2 peak) that mimics the pattern of monochlorinated compounds (Figure S2). This calculation should be regarded as a rough guidance rather than an exact value. Our manual calculations were corroborated by a web-based isotope pattern calculator, enviPat.^55^ Thus, pattern-matching approaches in case of larger compounds may misinterpret ^13^C-induced peaks as chlorine signatures or misidentify combined contributions from ^13^C and ^37^Cl as indicative of dichlorinated compounds, leading to both false positives or false negatives. To more accurately define the molecular weight range susceptible to such misinterpretations, we also considered the presence of other elements such as hydrogen, oxygen, and nitrogen. This limitation in mind, we decided to exclude compounds with molecular weights above 1000 Da in future queries and focus on smaller molecules, regardless of whether we were screening for mono-, di-, or polychlorinated compounds, to maintain a consistent and unified screening approach. In this situation, the advantage of MassQL over Haloseeker became clear. The MassQL query can be easily amended a priori to exclude compounds above 1000 Da for such an extensive screening (Figure S3). In addition, using single MassQL queries for monochlorinated, dichlorinated, or polychlorinated compounds simplified data evaluation, as it reduces the number of false positives, and increases the flexibility of setting the appropriate parameters (e.g., mass range) for screening a data set.

Using refined MassQL queries and taking these findings into account during the evaluation of HaloSeeker outputs, we next selected extracts for in-depth HPLC-HRMS analysis and dereplication. To do this, we focused on those extracts containing at least 20 features exhibiting characteristic chlorination or bromination isotope patterns (Figure S3 and Figure S4).^47,56^ As expected, no bromine-containing SMs were identified, since the media used for the cultivation of the cyanobacterial strains used to generate our extract collection did not contain any added bromide. From the library of biomass and media extracts screened, 11 extracts containing at least 20 features for chlorinated metabolites were shortlisted. Prioritization was further refined by dereplication to exclude known HSMs, thereby concentrating on extracts with novel chlorinated compounds. Both Haloseeker and MassQL, when applied independently, consistently identified this same subset of extracts. Among these, one biomass extract stood out due to its particularly rich profile of chlorinated features, warranting further in-depth analysis and isolation of HSMs.

This extract was derived from *Tolypothrix* sp. PCC9009 (Figure 1A). We detected 60/123 chlorinated features (pos./neg. ionization mode) in its biomass extract, and 81/89 features (pos./neg. ionization mode) in its medium extract. Fractionation of the biomass extract of this strain using flash chromatography resulted in five fractions. One of the fractions contained all chlorinated SMs detected during the initial screening. As dereplication using HRMS^2^ was unsuccessful, we proceeded to isolate the major compound, which was present in both biomass and medium extract (Figure 1A; [M + H]^+^, *m*/*z* 413.1147). This enabled us to extend the MS-based dereplication workflow by NMR-based structure elucidation. 1D- and 2D NMR spectroscopy rapidly identified the compound as anhydrocyanobacterin (**1**, Table 1, Figures 3 and 4), the anhydro congener of CB, which were both first isolated by Mason et al. and Pignatello et al.^33,38,39^

**Figure 1:**
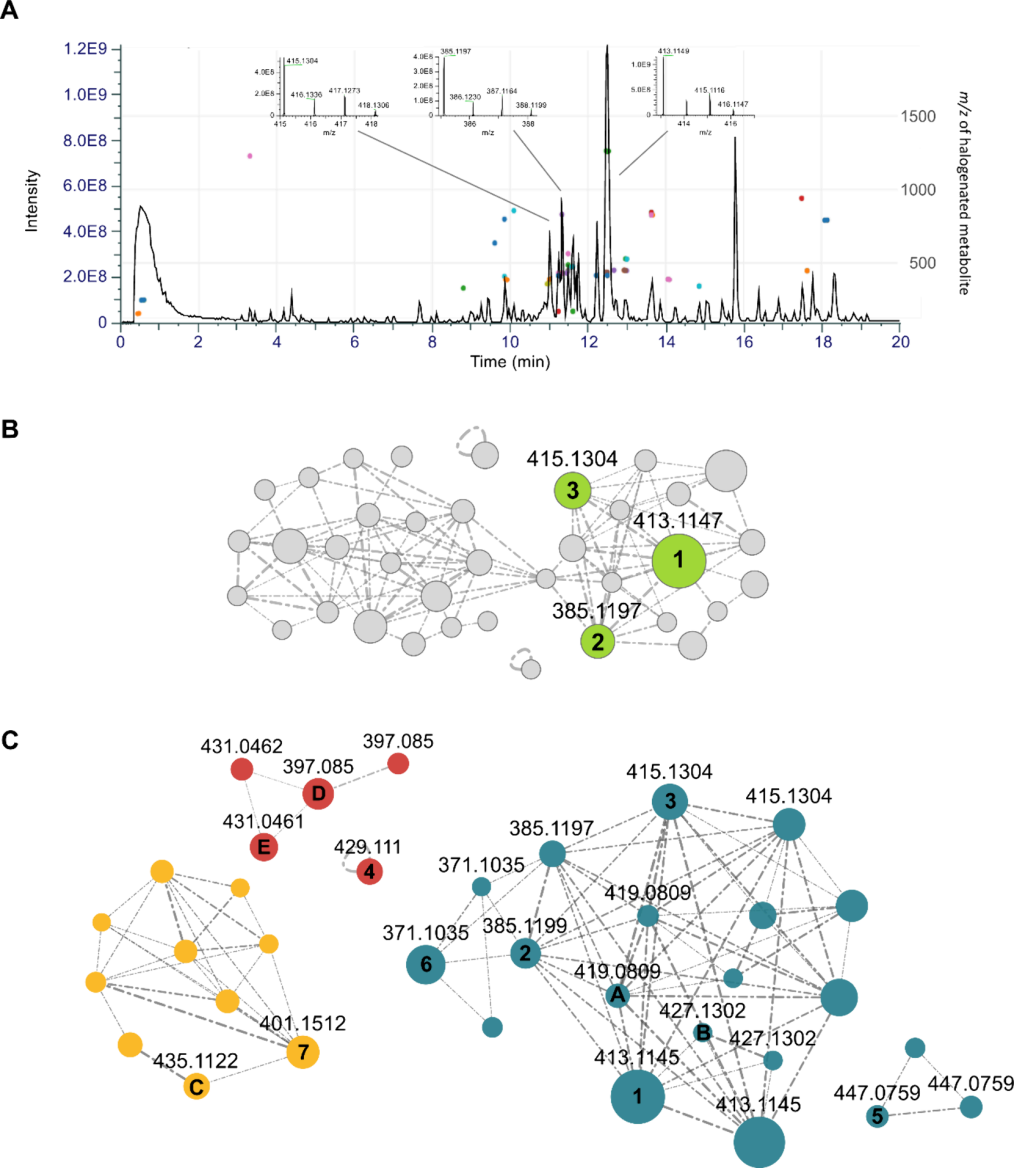
**A** Base-peak chromatogram of a *Tolypothrix* sp. PCC9009 biomass extract, overlaid over the respective HaloSeeker plot. Ionization in pos. mode. In the HaloSeeker plots, each HSM cluster is represented by a colored dot at the given retention time and *m*/*z* value (right *y*-axis). Isotope patterns of the molecular ion of representative compounds are shown. **B** Classical Molecular Networking analysis of the biomass extract of *Tolypothrix* sp., highlighting chlorinated HSMs clustering around the identified anhydrocyanobacterin. Nodes connected for cosine scores ≥0.7, edge width correlating to cosine score, node size correlating to precursor ion intensity. **1**, (*E*/*Z*)-**2**, and (*E*/*Z*)-**3** highlighted in green. **C** Feature Based Molecular Networking analysis for structure prediction and chemical space visualization of **1** analogues. Nodes connected for cosine scores ≥0.8, edge width correlating to cosine scores. HSMs only detectable in neg. mode are colored red ((*E*/*Z*)-**D**, (*E*/*Z*)-**E**, **4**). Analogues of **1** colored blue ((*E*/*Z*)-**2** to (*E*/*Z*)-**6**, **(** *E*/*Z*)-**A**, (*E*/*Z*)-**B**), analogues lacking the double bond linked to the γ-carbon of the lactone core structure colored yellow (**7**, **C**).

**Figure 2:**
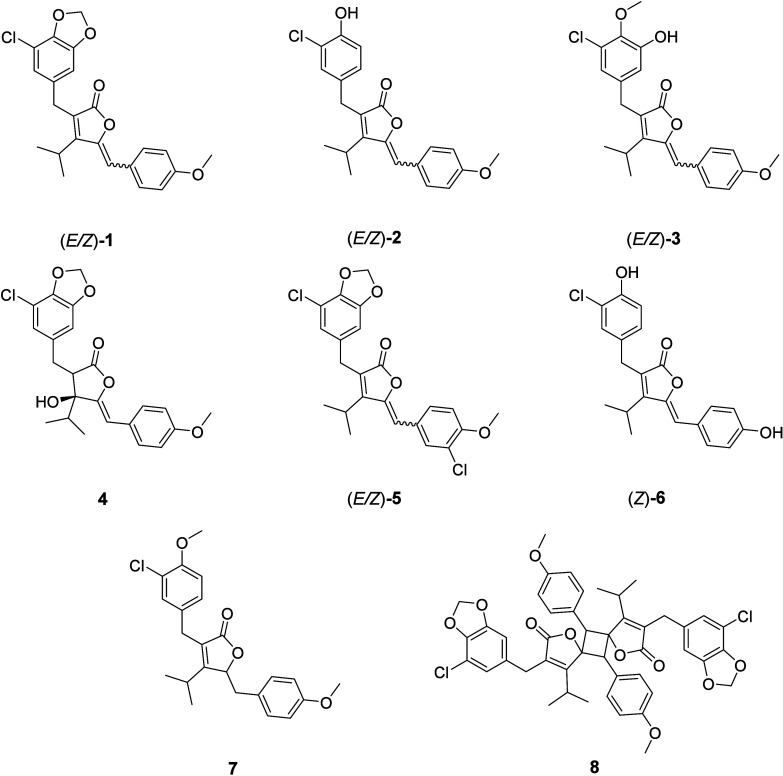
Structures of cyanobacterin/anhydrocyanobacterin analogues confirmed by NMR spectroscopy.

**Figure 3:**
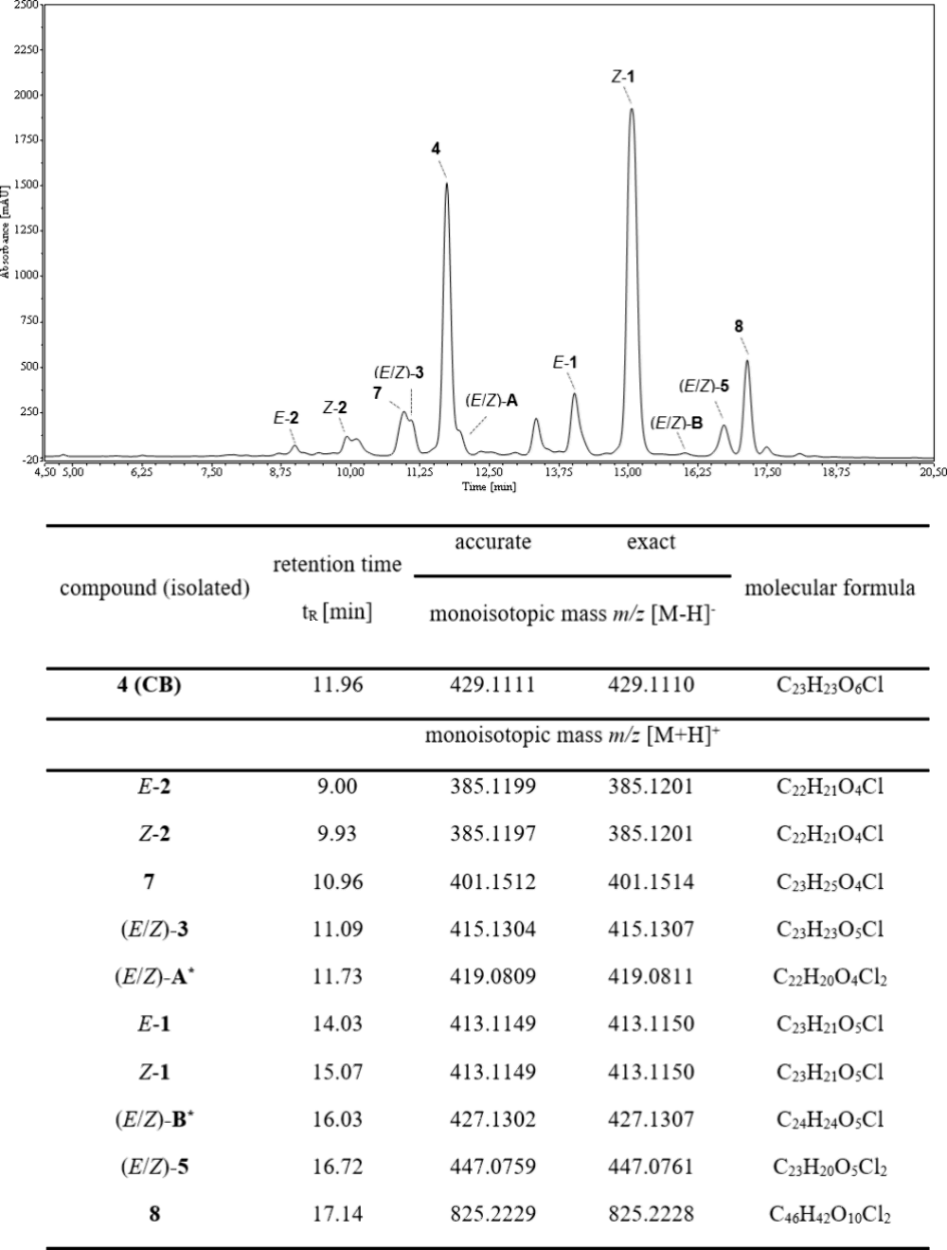
HPLC-UV chromatogram (λ = 210 nm, t_R_ 4.5–20.5 min) of the flash chromatography fraction of the biomass extract of *Tolypothrix* sp. PCC9009 containing chlorinated SMs. (*) Structures proposed based on HRMS^2^ and UV/vis spectroscopy data. Compounds **6** and **C**–**E** are not shown here, as they were either detected only by FBMN analysis or isolated from a different biomass batch.

**Figure 4:**
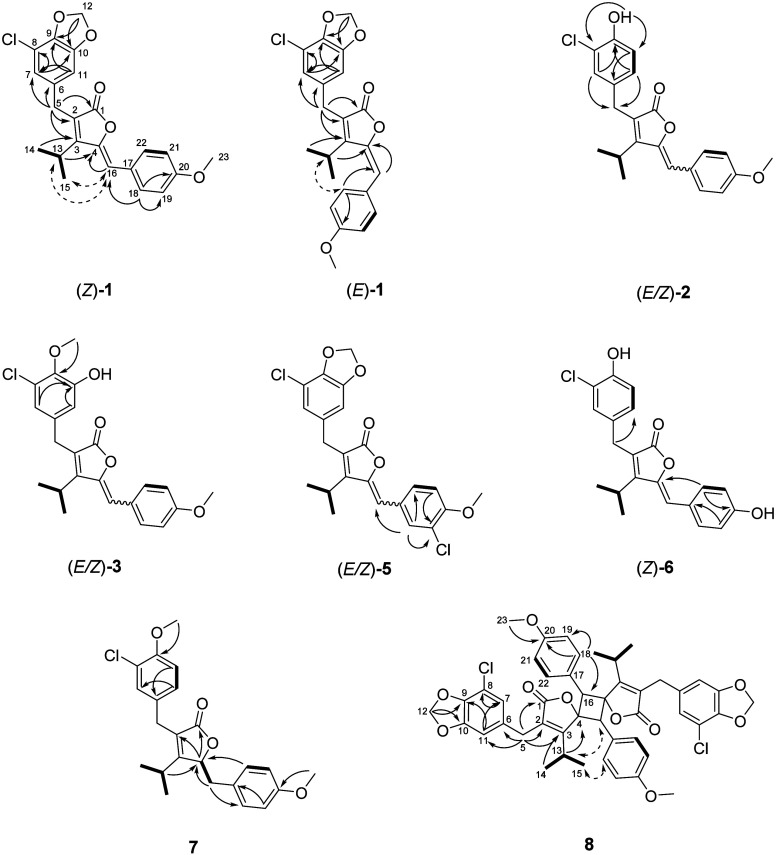
2D NMR correlations of **1**–**3** and **5**–**8**. COSY correlations bold, HMBC correlations solid arrows, NOESY/ROESY correlations dashed arrows; most correlations shown for **1**, key correlations shown for all other compounds.

**Table 1:** ^1^H (600 MHz) and ^13^C (600 MHz) NMR Spectroscopic Data of Compounds (*E*/*Z*)-**1**, (*E*/*Z*)-**2**, and (*E*/*Z*)-**3** in DMSO-*d*_6_

	*E* **-1**		*Z* **-1**		*E* **-2**		*Z* **-2**		*E* **-3**		*Z* **-3**	
position	δ_C_, type	δ_H_ (*J* in Hz)	δ_C_, type	δ_H_ (*J* in Hz)	δ_C_, type	δ_H_ (*J* in Hz)	δ_C_, type	δ_H_ (*J* in Hz)	δ_C_, type	δ_H_ (*J* in Hz)	δ_C_, type	δ_H_ (*J* in Hz)
1	169.5, C		169.9, C		169.6, C		170.0, C		169.5, C		169.9, C	
2	128.1, C		123.0, C		128.7, C		123.5, C		128.3, C		123.0, C	
3	157.0, C		158.6, C		156.5, C		158.2, C		156.8, C		158.6, C	
4	147.7, C		144.9, C		147.7, C		145.0, C		147.7, C		144.9, C	
5	29.1, CH_2_	3.75, s	28.2, CH_2_	3.69, s	28.5, CH_2_	3.71, s	27.6, CH_2_	3.66, s	28.9, CH_2_	3.71, s	28.0, CH_2_	3.65, s
6	133.3, C		133.7, C		129.6, C		130.1, C		134.8, C		135.1, C	
7	121.1, CH	6.73, d (1.3)	121.1, CH	6.73, d (1.3)	129.2, CH	7.15, d (2.2)	129.3, CH	7.17, d (2.1)	119.3, CH	6.72, d (1.7)	119.3, CH	6.73, d (1.7)
8	112.2, C		112.2, C		119.4, C		119.4, C		126.7, C		126.7, C	
9	142.4, C		142.4, C		151.5, C		151.5, C		151.4, C		151.4, C	
10	148.5, C		148.5, C		116.6, CH	6.88, d (8.3)	116.7, CH	6.89, d (8.4)	142.1, C		142.1, C	
11	107.5, CH	6.76, d (1.3)	107.5, CH	6.76, d (1.3)	127.6, CH	6.95, dd (8.3, 2.2)	127.6, CH	6.96, dd (8.4, 2.1)	115.3, CH	6.65, d (1.7)	115.3, CH	6.65, d (1.7)
12	102.0, CH_2_	6.09, s	102.0, CH_2_	6.10, s					59.9, CH_3_	3.71, s	59.9, CH_3_	3.70, s
13	25.4, CH	2.78, hept (7.1)	25.5, CH	3.25, hept (7.1)	25.4, CH	2.71, hept (7.1)	25.5, CH	3.24, hept (7.1)	25.4, CH	2.77, hept (7.0)	25.5, CH	3.24, hept (7.0)
14/15	20.8, CH_3_	1.06, d (7.1)	21.3, CH_3_	1.27, d (7.1)	20.8, CH_3_	1.05 (7.1)	21.2, CH_3_	1.27, d (7.1)	20.8, CH_3_	1.05, d (7.0)	21.3, CH_3_	1.28, d (7.0)
16	113.8, CH	6.97, s	110.4, CH	6.55, s	113.8, CH	6.98, s	110.2, CH	6.54, s	113.8, CH	7.00, s	110.4, CH	6.57, s
17	125.0, C		125.8, C		125.0, C		125.9, C		125.0, C		125.8, C	
18/22	130.7, CH	7.34, d (8.7)	132.1, CH	7.79, d (8.9)	130.7, CH	7.33, d (8.3)	132.1, CH	7.78, d (8.9)	130.7, CH	7.33, d (8.5)	132.1, CH	7.78, d (8.5)
19/21	115.5, CH	6.98, d (8.7)	114.4, CH	7.02, d (8.9)	115.3, CH	6.97, d (8.3)	114.4, CH	7.02, d (8.9)	115.5, CH	6.98, d (8.5)	114.4, CH	7.02, d (8.5)
20	159.4, C		159.7, C		159.4, C		159.7, C		159.4, C		159.7, C	
23	55.2, CH_3_	3.78, s	55.2, CH_3_	3.80, s	55.2, CH_3_	3.78, s	55.2, CH_3_	3.80, s	55.3, CH_3_	3.79, s	55.3, CH_3_	3.80, s

With **1** identified, we used CMN^48^ to obtain a first overview over the chemical space of structurally related SMs produced by the strain. Using the MS^2^ feature corresponding to the [M + H]^+^ ion of **1** as the molecular networking reference node, we identified its cluster comprising 34 additional nodes (Figure 1B). The first evaluation indicated that most of these nodes represented structural analogues and not adducts or fragment ions. Surprisingly, we could not identify CB in the cluster. Among the other chlorinated metabolites, two molecular ions [M + H]^+^ at *m*/*z* 385.1197 (**2**, calc. molecular formula: C_22_H_21_O_4_Cl) and *m*/*z* 415.1304 (**3**, calc. molecular formula: C_23_H_23_O_5_Cl) stood out (Figure 1B). Based on the calculated sum formulas, we hypothesized that **2** might be an anhydro congener of the previously described hydroxycyanobacterin.^57^ Lee et al. outlined its instability based on the activity loss after 2–3 days, assuming dehydration. They demonstrated that hydroxycyanobacterin is an effective inhibitor of photosynthetic electron transport in isolated thylakoid membranes, with potency comparable to the parent compound. However, no NMR data have been published for **2** to date. Although we did not detect a nonchlorinated version of **2**, such an analogue was identified during the heterologous reconstitution of cyanobacterin biosynthesis in *E. coli*.^58^ In that study, the flavin-dependent halogenase CybI was identified within the cyb biosynthetic gene cluster, suggesting that chlorination is a late-stage modification in the biosynthetic pathway.^58^ In contrast, **3** appeared to be a previously unknown analogue of **1** and **4**.

To further explore the chemical space surrounding the observed compounds and facilitate their structural identification, we performed an in-depth FBMN analysis (Figure 1C). A previous study has revealed the existence of *E*/*Z* isomers of **1**,^38^ prompting us to investigate whether similar isomerism is exhibited by other analogues of **1** or if alternative core structures can be identified. We identified two clusters from the positive ionization mode data. The first cluster contained all **1**-related compounds. Within this cluster, besides **1** itself, six distinct pairs of nodes were observed, each pair sharing identical *m*/*z* values but differing in retention times (Figure 1C). This suggested the presence of constitutional or configurational isomers. In contrast, the second cluster did not contain nodes with the same *m*/*z* values, suggesting the presence of other structurally related compounds (Figure 1C). Both clusters were observed in positive and negative ionization mode. A third cluster was observed in negative ionization mode data only. These findings motivated us to conduct a microfractionation study aimed at identifying potentially herbicidal CB analogues, followed by targeted isolation and structure elucidation.

### Bioactivity-Guided Microfractionation

As CB as well as the previously reported hydroxycyanobacterin have herbicidal activity,^57^ we microfractionated the biomass extract of *Tolypothrix* sp. PCC9009 to identify further related compounds with this activity. Only one microfraction showed growth inhibition of the CB-sensitive cyanobacterial strain *Synechocystis* sp. PCC6803 (Figure S5). Subsequent isolation and NMR-based structure elucidation showed that this microfraction contained CB (**4**). We found that the reason why we could not identify **4** during the initial manual dereplication was quantitative loss of water during ESI ionization (Figure S10A). By reducing the transfer capillary temperature and using freshly isolated **4**, we later obtained mass spectra of intact **4** in both positive and, more efficiently, in negative ionization mode (Figure S10B). As to our surprise none of the other fractions showed herbicidal activity, we wondered what structural differences would cause these analogues lack activity. Studies by Gleason et al. demonstrated that a hydroxy group in the lactone core structure is essential and that chlorination in ortho position to the methylenedioxy group (Figure 2) is essential for biological activity,^37,38,40^ suggesting that most of the compounds in the CMN and FBMN analysis might represent anhydro congeners or analogues with chlorination at a different position.

### Isolation and Structure Elucidation

We isolated eight compounds (**1**–**8**, Figure 2A) from the HSM-containing flash chromatography fraction using semipreparative HPLC. Their structures were elucidated by 1D and 2D NMR experiments (Tables 1 and 2; Figures S16–S69). In addition to the CMN and FBMN analyses, comparison of their UV/vis spectra further supported their structural relatedness and revealed three distinct UV/vis spectra types matching the FBMN clusters (Figure S6). Compounds structurally closely related to **1** were assembled in the first FBMN cluster (Figure 1C). The compounds assembled in the second FBMN cluster, comprising **7**, **8**, and **C**, lacked a characteristic absorption maximum around 359 nm observed in the first group, suggesting differences in their conjugated π-systems. Compound **4** displayed a distinct UV/vis spectrum (Figure S6), setting it apart from **1** and related compounds. It appeared as a single, isolated node in the network. The UV/vis spectra observed for **1**–**8** are in excellent agreement with the structures that were later elucidated by NMR spectroscopy, confirming the consistency of the observed absorption features with the assigned molecular structures.

**Table 2:** ^1^H (600 MHz) and ^13^C (600 MHz) NMR Spectroscopic Data of Compounds (*E*/*Z*)-**5** and *Z*-**6** in DMSO-*d*_6_

	*E* **-5**		*Z* **-5**		*Z* **-6**	
position	δ_C_, type	δ_H_ (*J* in Hz)	δ_C_, type	δ_H_ (*J* in Hz)	δ_C_, type	δ_H_ (*J* in Hz)
1	169.4, C		169.7, C		170.0, C	
2	128.6, C		123.7, C		123.0, C	
3	156.9, C		158.6, C		158.2, C	
4	148.3, C		145.7, C		144.3, C	
5	29.1, CH_2_	3.76, s	28.2, CH_2_	3.70, s	27.6, CH_2_	3.65, s
6	133.2, C		133.6, C		130.2, C	
7	121.1, CH	6.75, d (1.6)	121.1, CH	6.75, d (1.6)	129.2, CH	7.16, d (2.1)
8	112.2, C		112.2, C		119.4, C	
9	142.3, C		142.5, C		151.4, C	
10	148.5, C		148.6, C		116.7, CH	6.88, d (8.4)
11	107.6, CH	6.77 d (1.6)	107.6, CH	6.77, d (1.6)	127.6, CH	6.96, dd (8.4, 2.1)
12	102.0, CH_2_	6.10, s	102.0, CH_2_	6.10, s		
13	25.5, CH	2.71, hept (7.1)	25.6, CH	3.25, hept (7.1)	25.5, CH	3.23, hept (7.1)
14/15	20.8, CH_3_	1.08 (7.1)	21.2, CH_3_	1.28, d (7.1)	21.3, CH_3_	1.26, d (7.1)
16	113.9, CH	6.96, s	109.0, CH	6.58, s	110.7, CH	6.47, s
17	126.1, C		126.9, C		124.3, C	
18	130.7, CH	7.53, d (2.2)	131.4, CH	7.93, d (2.2)		
19	120.8, C		121.3, C			
21	112.5, CH	7.19, d (8.5)	113.1, CH	7.25, d (8.8)	115.8, CH	6.83, d (8.8)
22	129.5, CH	7.37, dd (8.5, 2.2)	130.9, CH	7.79, dd (8.8, 2.2)	132.3, CH	7.67, d (8.8)
23	56.2, CH_3_	3.82, s	56.3, CH_3_	3.90, s		

The HRMS data and the FBMN analysis (Figure 1C) indicated that **1**, **2**, **3**, **5**, and **6** exist as pairs of isomers, as evidenced by distinct molecular features with identical exact masses but differing retention times. Although the data initially indicated possible isomerism, the precise nature could not be determined at that stage. Subsequent isolation and NMR analysis confirmed the presence of *E* and *Z* isomers, as evidenced by characteristic differences in chemical shifts and unambiguous NOE correlations (Figure 4, Tables 1 and 2). In the following, this is illustrated in detail for **1**, which serves as a representative case for the *E*/*Z* isomerism observed among the aforementioned compounds. For the *Z* isomer, NOE correlations were observed between the olefinic proton H-16 (δ_H_ 6.55 ppm) and both the methine proton at H-13 (δ_H_ 3.25 ppm) and the methyl protons (H-14, H-15; δ_H_ 1.27 ppm) of the isopropyl moiety. Conversely, for the *E* isomer, a NOE correlation was observed between the methine proton at H-13 (δ_H_ 2.78 ppm) and the aromatic proton H-18 (δ_H_ 7.34 ppm). Moreover, ^1^H and ^13^C chemical shifts supported the *E*/*Z* configuration assignments: For the *E* isomer, the olefinic carbon C-16 and its proton resonate at δ_C_ 113.8 ppm and δ_H_ 6.98 ppm, respectively, appearing more deshielded compared to the *Z* isomer (δ_C_ 110.2 ppm, δ_H_ 6.54 ppm). Likewise, the C-4 at the γ-position was found at δ_C_ 147.7 ppm in *E*-**1**, in contrast to δ_C_ 145.0 ppm in *Z*-**1**. The methyl protons of the isopropyl group (H-14, H-15) are slightly more shielded in *E*-**1** (δ_H_ 1.06 ppm) compared to *Z*-**1** (δ_H_ 1.27 ppm). These characteristic differences in chemical shifts reflect the distinct spatial environments in the two isomers and are consistent with the assigned configurations. The use of NOE correlations and the characteristic chemical shift differences were also used to distinguish *E*/*Z* isomers in the structure elucidation of the structurally related enhygrolides.^59^

Upon isolation of **1**, **2**, **3**, and **5**, a rapid *E*/*Z* equilibrium was established (Figures S7A, S8A, S9A, and S11A), resulting in the coexistence of both isomers in the final NMR samples (Figures S16, S22, S28, S34, and S45). In the case of **2**, **3**, and **5**, the chromatographic conditions used during a first isolation were insufficient to separate the *E* and *Z* isomers. Given the swift isomerization observed after separating *E*-**2** and *Z*-**2**, we expected similar behavior for **3** and **5** and therefore did not pursue further separation attempts (Figures S9A and S11A). Nevertheless, both isomers remained distinguishable in the ^1^H and ^13^C NMR spectra (Tables 1 and 2) and were confidently assigned as *E* or *Z* based on different chemical shifts of the olefinic proton H-16 and the corresponding C-16, the resonance signals of the isopropyl moiety, and key NOE correlations, as exemplified above (Figure 4).

Compound *Z*-**1** is the major compound present in the biomass and medium extracts, isolated as a pale-yellow amorphous powder. Shortly after isolation, both isomers were detected in the sample due to isomerization (Figure S7). The ion intensities for both isomers of **1** in the biomass extract suggest that the *Z* isomer is more stable. This observation aligns with previously reported findings, where equilibrium was reached after 7 days, with the *Z* isomer predominating.^38^ D’Agostino et al. made similar observations,^58^ but we did not detect complete interconversion to the *Z* isomer. HRMS analysis resulted in an [M + H]^+^ ion at *m*/*z* 413.1149 (C_23_H_22_O_5_Cl, calc. 413.1150, Δ 0.2 ppm). The presence of one chlorine substituent in the molecule was evident from the isotope pattern. The NMR data of **1** matched the published data of anhydrocyanobacterin (Table 1, Figures S16–S21), which has been isolated from the same strain before.^38^

Compounds *E*-**2** and *Z*-**2** were isolated separately as pale-yellow amorphous powders. HRMS analysis resulted in both cases in an [M + H]^+^ ion at *m*/*z* 385.1199 (C_22_H_22_O_4_Cl, calc. 385.1201, Δ 0.5 ppm). The characteristic isotope pattern indicated the presence of a single chlorine atom. UV/vis absorption data, in combination with the molecular formula, suggested that **2** is an analogue of **1** featuring a single hydroxy group in place of the methylenedioxy moiety present in **1**. This structural modification was corroborated by the ^13^C NMR spectrum (Table 1 and Figure S23), which exhibits a signal at δ_C_ 151.5 ppm, assigned to C-9, consistent with a phenolic carbon. In contrast to **1**, the ^1^H-spectrum (Table 1 and Figure S22) featured a doublet of doublets at δ_H_ 6.95 ppm (H-11, *J* = 8.3 Hz and *J* = 2.2 Hz), a doublet at δ_H_ 7.15 ppm (H-7, *J* = 2.2 Hz), and an additional doublet at δ_H_ 6.88 ppm (H-10, *J* = 8.3 Hz). These coupling patterns, revealing both ortho and meta interactions, are absent in **1** and align with the proposed replacement of the methylenedioxy group with a hydroxy group at C-9 in **2**. ^1^H–^13^C-HMBC correlations between H-10 and C-8/C-8 (δ_C_ 129.6/119.4 ppm) as well as between H-7/H-11 and C-9 further supported the structure and confirmed the position of the hydroxy group (Figure 4). The remaining proton and carbon signals are in good accordance with the data for **1**. As we hypothesized in the first CMN analysis, **2** is the anhydro congener of the previously reported hydroxycyanobacterin.^57^ We named the compound anhydrocyanobacterin C.

Compounds *E*/*Z*-**3** were coisolated as a pale-yellow amorphous powder. HRMS analysis revealed [M + H]^+^ ions at *m*/*z* 415.1304 (C_23_H_24_O_5_Cl, calc. 415.1307, Δ 0.7 ppm), consistent with an analogue of **1** featuring one additional methyl and one additional hydroxy group compared to *E*/*Z*-**2**. This structural assignment was further supported by UV/vis absorption data and similarities in HMBC correlations and ^13^C chemical shifts compared to *E*/*Z*-**2** (Figure 4 and Table 1). Among these, a notable difference was observed for C-10, which appeared deshielded at δ_C_ 142.1 ppm, suggesting an additional oxygen at this position (Table 1 and Figure S35). Both H-7 and H-11 showed HMBC correlations with C-10, which further supported the presence of a hydroxy group at this carbon. Moreover, the ^1^H NMR spectrum featured doublets at δ_H_ 6.73 ppm (H-7, *J* = 1.7 Hz), and at δ_H_ 6.65 ppm (H-11, *J* = 1.7 Hz), indicative of meta coupling, which is further supported by COSY correlations. The ^13^C NMR spectrum displayed an additional methoxy carbon signal (C-12, δ_C_ 59.9 ppm, δ_H_ 3.71 ppm) which showed an HMBC correlation to C-9. Taken together, these data support the presence of a hydroxy group at C-10 and a methoxy substituent at C-9, in line with the proposed structure. **3** was named anhydrocyanobacterin E.

NMR and HRMS data of **4** matched the published data of cyanobacterin (Tables 3, S4, Figures S40–S44), which has been isolated from the same strain before (*S. hofmanni* UTEX 1581).^33,36,38,39^ Compounds *E*/*Z*-**5** were coisolated as a pale-yellow amorphous powder. HRMS analysis resulted in [M + H]^+^ ions at *m*/*z* 447.0759 (C_23_H_21_O_5_Cl_2_, calc. 447.0761, Δ 0.4 ppm). The presence of two chlorine substituents in the molecule was evident from the isotope pattern. The combined evidence from the UV/vis spectrum and the molecular formula supported the assignment of *E*/*Z*-**5** as analogues of **1** with an additional chlorination, a conclusion further reinforced by highly similar ^13^C NMR and HMBC data, with the exception of a deshielded C-19 signal (δ_C_ 120.8 ppm) attributed to chlorination in this position (Table 2 and Figures S46 and S49). Key ^1^H–^13^C-HMBC correlations supported the supposed position of the chlorine (H-22/H-21 to C-19, Figure 4). *E*/*Z*-**5** was named anhydrocyanbobacterin F.

Compound *Z*-**6** was isolated as a pale-yellow amorphous powder. Interestingly, no isomerization of the isolated compound was observed within 3 days. Nevertheless, the *E*-isomer was detectable in the HRMS data (Figure 1C), indicating that isomerization occurs, albeit at a slower rate compared to the other compounds. HRMS analysis resulted in an [M + H]^+^ ion at *m*/*z* 371.1042 (C_21_H_20_O_4_Cl, calc. 371.1045, Δ 0.8 ppm), which, in conjunction with the UV/vis spectrum, indicated that *Z*-**6** was an analogue **1** of lacking a methyl group in comparison to **2**. Indeed, the methoxy signal was missing in the ^13^C NMR spectrum (Figure S52). The slower isomerization might be explained by the fact that the missing methyl group allows mesomeric stabilization involving the distant lactone carbonyl group (vinylogous/phenylogous carboxylic acid). We named the compound anhydrocyanobacterin B.

Compound **7** was isolated as a white amorphous powder. Interestingly, manual inspection of the HRMS data and the FBMN analysis showed that **7** did not show *E*/*Z* isomerism (Figure 1C). Moreover, the UV/vis spectrum differed from those of compounds related to **1**. HRMS analysis resulted in an [M + H]^+^ ion at *m*/*z* 401.1512 (C_23_H_26_O_4_Cl, calc. 401.1514, Δ 0.5 ppm). The calculated molecular formula suggested that **7** had an additional methyl group compared to **2** and lacked a double bond. Indeed, the δ_C_ values (Table 3 and Figures S59 and S60), assigned based on HSQC and HMBC correlations, revealed an additional methoxy group (C-12, δ_H_ 3.80 ppm, δ_C_ 55.5 ppm) with HMBC correlations to C-9, analogous to the methoxy substitution observed in **3** (Figure 4). The absence of the double bond at the γ-position of the furanolid core structure was obvious due to the increased shielding and multiplicity of C-4 (δ_C_ 81.8 ppm, δ_H_ 5.31 ppm) and C-16 (δ_C_ 37.0 ppm, δ_H_ 2.76/3.29 ppm). The absolute configuration at C-4 has not yet been determined. **7**, dihydroanhydrocyanobacterin D, is the first representative of a dihydro-analogue of **1**.

**Table 3:** ^1^H (600 MHz) and ^13^C (600 MHz) NMR Spectroscopic Data of **4**, **7**, and **8** in DMSO-*d*_6_

	**4**		**7**		**8**	
position	δ_C_, type	δ_H_ (*J* in Hz)	δ_C_, type	δ_H_ (*J* in Hz)	δ_C_, type	δ_H_ (*J* in Hz)
1	173.5, C		173.6, C		172.1, C	
2	50.7, CH	3.45, t (6.8)	124.6, C		126.2, C	
3	81.1, COH	5.77, s OH	169.7, C		168.0, C	
4	149.9, C		81.8, CH	5.31, dd (7.2, 3.8)	92.7, C	
5	27.9, CH_2_	2.93, d (6.8)	27.4, CH_2_	3.44, d (3.5 Hz)/3.51, d (3.5 Hz)	28.3, CH_2_	3.60, s
6	134.5, C		131.5, C		133.1, C	
7	122.0, CH	6.94, s	129.3, CH	7.14, d (2.1)	120.3, CH	6.25, s
8	111.9, C		120.6, C		112.0, C	
9	142.2, C		152.7, C		142.3, C	
10	148.3, C		112.4, CH	6.89, d (8.6)	148.4, C	
11	108.3, CH	6.94, s	127.3, CH	6.64, dd (8.6, 2.1)	107.2, CH	6.45, s
12	101.9, CH_2_	6.10, s	55.5, CH_2_	3.80, s	102.0, CH_2_	6.06, s
13	32.6, CH	2.07, hept (6.6)	26.9, CH	2.98, hept (7.1)	25.8, CH	3.26, hept (7.0)
14/15	17.7/15.8, CH_3_	0.76/0.98, d/d (6.6)	19.7/21.3, CH_3_	1.16, d (7.1)	21.2, CH_3_	1.23, d (7.0)
16	104.2, CH	5.72, s	37.0, CH_2_	2.75, dd (14.7, 7.2)/3.29, dd (7.2)	49.5, CH	4.59, s
17	126.1, C		127.9, C		123.0, C	
18/22	129.7, CH	7.49, d (8.8)	130.5, CH	7.16, d (8.6 Hz)	130.6, CH	7.23, d (8.7 Hz)
19/21	114.0, CH	6.93, d (8.8)	113.5, CH	6.85, d (8.6 Hz)	113.9, CH	6.87, d (8.7 Hz)
20	158.1, C		158.0, C		159.0, C	
23	55.1, CH_3_	3.75, s	54.6, CH_3_	3.74, s	54.9, CH_3_	3.71, s

Compound **8** was isolated as a white amorphous powder. HRMS analysis ([M + H]^+^ at *m*/*z* 825.2229, C_46_H_43_O_10_Cl_2_, calc. 825.2228, Δ 0.1 ppm) suggested **8** to be a dimer based on two monomers of **1**. HMBC and ^13^C NMR data showed that the compound is a symmetric homodimer (only 23 carbons observed in the ^13^C NMR spectrum, Table 3 and Figure S63) similar to compound **1**, except for the chemical shifts for the furanolide core structure. These were better comparable to the more saturated **7** (C-4 δ_C_ 92.7 ppm, C-16 δ_C_ 49.5 ppm), indicating the lack of the double bond. No proton was found to be attached to C-4, however, and H-16 (δ_H_ 4.59 ppm) is a singlet with an integral of 2, corresponding to one proton per monomer unit, consistent with a symmetric homodimer. These data support a linkage between C-4 of one monomer and C-16 of the other. HPLC-HRMS analysis revealed two distinct species with identical *m*/*z* values ([M + H]^+^ at *m*/*z* 825.2224), suggesting the presence of isomeric forms of **8** (Figure S14). These may represent either constitutional isomers, arising from different dimerization patterns (e.g., “head-to-head” vs “head-to-tail” linkage of the monomer units), or configurational isomers. Such covalently linked dimers have also been reported in cyanobacterial SMs, for example, nostotrebin 6-related compounds described by Kossack et al., which arise via dimerization of monomeric units.^60^ Given the planar structure of **8**, the presence of diastereomers is conceivable. However, this has not been studied yet. Further experimental evidence is required to clarify the configuration of the observed compounds. Compound **8** was named bisanhydrocyanobacterin, and is the first reported cyanobacterial cyclobutane-linked dimer. SM cyclobutane-linked dimers, formed via an intermolecular [2 + 2] cycloaddition that results in homodimers or heterodimers, are well-known in plant-derived SMs, including alkaloids, flavonoids, terpenoids, and phenylpropanoids.^61–63^ Marine representatives comprise SMs from sponges, e.g. screptins,^64,65^ macroalgae, e.g. pulchralides A-C,^66^ or marine fungi, e.g. diasteltoxin A-C^67^ or dipleosporalones A and B.^68^ The reported cyclobutane-containing SMs are frequently related to co-occurring monomeric precursors in their natural sources, featuring conjugated alkenes as chromophores.^63–66^ Photoinduced intermolecular [2 + 2] cycloaddition has been tentatively proposed and partially demonstrated for plant-derived cyclobutane-containing dimers.^63,69–73^ However, this mechanism could not be shown for diasteltoxins A–C or dipleosporalones A and B. Instead, enzymatic dimerization has been suggested for these compounds, although this has yet to be experimentally confirmed.^63,67,68^ To investigate whether the formation of **8** is a nonenzymatic photochemical process, we irradiated *E*/*Z*-**1** with UV light (254 nm). At predetermined time intervals, samples were collected for analysis using HPLC-HRMS (Figure S15). Indeed, we observed the formation of **8** under UV irradiation, supporting a nonenzymatic [2 + 2] photochemical cycloaddition as a plausible pathway. As **8** was also detectable in fresh cyanobacterial cultures, it is unlikely to be an artifact formed solely during storage or isolation. Whether **8** is produced enzymatically or via the described [2 + 2] photocycloaddition in the photoautotrophic cyanobacterium under natural light conditions remains unresolved.

Targeted re-examination of the *Tolypothrix* sp. PCC9009 biomass extract for other dimers led to the identification of a possible dimer derived from two monomers of **3** ([M + H]^+^ *m*/*z* 829.2552, C_46_H_47_O_10_Cl_1_, calc. 829.2541, Δ 1.3 ppm), as well as a dimer based on **5** ([M + H]^+^ at *m*/*z* 893.1443, C_46_H_41_O_10_Cl_4,_ calc. 893.1448, Δ 0.6 ppm). HRMS analysis revealed two additional dimers, for which the corresponding monomers could not be assigned ([M + H]^+^ *m*/*z* 831.1898, C_45_H_42_O_9_Cl_3_, calc. 831.1889, Δ 1.1 ppm; [M + H]^+^ *m*/*z* 827.2392, C_46_H_45_O_10_Cl_2_, calc. 827.2384, Δ 1.0 ppm). In contrast to the other two characterized dimers, no fragment ions corresponding to the monomers could be detected in the HRMS^2^ spectra (Figure S70).

Compounds *E*/*Z*-**A**, *E*/*Z*-**B**, **C**, *E*/*Z*-**D**, and *E*/*Z*-**E** (Figure S71) could not be isolated in sufficient amounts for unambiguous structure elucidation. **C** ([M + H]^+^ *m*/*z* 435.1122, C_23_H_25_O_4_Cl_2_, calc. 435.1124, Δ 0.5 ppm), *E*/*Z*-**D** ([M – H]^−^ *m*/*z* 397.0851, C_22_H_18_O_5_Cl, calc. 397.0848, Δ 0.8 ppm), and *E*/*Z*-**E** ([M – H]^−^ *m*/*z* 431.0462, C_22_H_17_O_5_Cl_2_, calc. 431.0459, Δ 0.7 ppm) were only identified during a detailed FBMN analysis of the HRMS data in both positive and negative ionization mode. The structural proposals are based on the FBMN analysis, the HRMS^2^ data (Figures S72–S76, Tables S8–S12), and the previously described CB biosynthesis pathway.^58^

Given that the isolated compounds **1**–**3** and **5**–**7** were all anhydro congeners of cyanobacterin analogues, we hypothesized that their corresponding 3-hydroxy analogues might also be present in the extract of *Tolypothrix* sp. PCC9009. Indeed, upon closer inspection of the HRMS data, we detected signals consistent with putative 3-hydroxy congeners for **2** ([M + H]^+^ *m*/*z* 403.1309, C_22_H_24_O_5_Cl, calc. 403.1307, Δ 0.5 ppm), **3** ([M + H]^+^ *m*/*z* 433.1414, C_23_H_26_O_6_Cl, calc. 433.1412, Δ 0.5 ppm), and **B** ([M + H]^+^ *m*/*z* 445.1415, C_24_H_26_O_6_Cl, calc. 445.1412, Δ 0.7 ppm) (Figure S77), although only in very low abundance. These findings align with our observation that purified **4** gradually converts into **1**, as evidenced by the appearance of the latter in LC-MS profiles of stored samples. Since the 3-hydroxy group within the γ-lactone core is essential for bioactivity, these putative 3-hydroxy congeners may also show herbicidal activity.^37,38^ Attempts to isolate larger quantities or to detect other 3-hydroxy congeners directly from fresh cultures were unsuccessful.

Besides compounds **1**–**7**, several other known naturally occurring SMs contain a central α,β- unsaturated γ-lactone. The nostoclides, isolated from a marine *Nostoc* sp.,^74^ resemble **1**–**7** by featuring 2,4-benzyl/benzylidene substituents and an isopropyl group at C3. Synthetic nostoclide analogues have also been shown to inhibit plant growth and photosynthesis, and nostoclide I and II have been reported to exhibit moderate cytotoxicity against Neuro-2a CCL and KB CCL-17 cell lines. Like **1**–**8**, nostoclides are readily excreted into the surrounding medium, which, together with their herbicidal activity, suggests a potential allelopathic role.^74–76^ Enhygrolides A and B from the marine myxobacterium *Enhygromyxa salina* differ from nostoclides and **1**–**7** by featuring an isobutyl group at C3 and varying aromatic substitutions. Similar to the *Z*/*E* isomerism observed in **1**–**3** and **5**–**6**, the enhygrolides occur as *Z* and *E* isomers, whereas cyanobacterin, nostoclides, and the majority of their synthetic analogues appear exclusively as *Z* isomers.^33,38,39,59,74–78^ Angiolacton from the terrestrial myxobacterium *Angiococcus* sp. carries an isopentenyl group at C2 and hydroxy-/hydroxybenzyl groups at C3.^79^ Rubrolides A–H, first described from the tunicate *Ritterella rubra*, and rubrolides I–N, isolated from *Synoicum blochmanni*, are inhibitors of photosynthetic electron transport. Some natural and synthetic congeners also exhibit cytotoxicity. Although differing from **1**–**7** in α-/β-substitution of the γ-lactone ring, rubrolides share a γ-benzylidene moiety with a hydroxy group positioned as in **6**. Several congeners are ortho-brominated, similar to the chlorination in **5**, and rubrolide F contains a methoxy group, as found in **1**–**4** and **6**–**7**.^80–83^

### Bioactivity Characterization

In addition to the herbicidal activity discussed above, we assessed the cytotoxicity of **1**, **4**, and **7** against HCT116 human colon carcinoma cells *in vitro* using the sulforhodamin B (SRB) assay in concentrations from 0.1 μM to 50 μM. Although some dose-dependency was discernible, the compounds showed only slight cytotoxicity even at the highest concentration tested (Table S13).

## Conclusion

We evaluated two approaches, HaloSeeker and MassQL, for the detection of HSMs in a library of cyanobacterial extracts, and established an effective and flexible HRMS-based screening workflow. Accounting for limitations of isotope pattern recognition, particularly in high-molecular-weight compounds, reduced false positives. The workflow was extended by CMN/FBMN analysis and supported the discovery of cyanobacterin analogues in a selected cyanobacterium extract. Structures of nonisolated analogues could rapidly be suggested after NMR-based structure elucidation of compounds **1**–**8**. The isolated compounds, which were also found in higher amounts in the cultivation medium, comprised mostly anhydro congeners of cyanobacterin analogues, including the first described dimeric analogue.

## Experimental Section

### General Experimental Procedures

NMR spectra were recorded in DMSO-*d*_6_ on a Bruker Avance III (Bruker BioSpin GmbH) equipped with a QCI cryoprobe with one axis self-shielding gradient, operating at 600 MHz (^1^H) or 150 MHz (^13^C) at 300 K. Chemical shifts are reported in ppm, spectra were calibrated related to solvent’s residual proton chemical shift (δ_H_ 2.50, δ_C_ 39.5). NMR data were analyzed with MestReNova (version 14.3.0-30573, Mestrelab Research S.L.) after processing using Auto Phase Correction and Auto Baseline Correction. HPLC separations were performed with an Agilent 1260 Infinity II with a diode array detector (1260 DAD) and a Dionex UltiMate 3000 (Thermo Scientific). LC-MS/MS data were acquired as described below.

### Cyanobacterial Material: Strain, Media and Growth Conditions

The strain *Tolypothrix sp.* is part of the Pasteur Culture Collection, Institut Pasteur, France (accession number PCC9009). The strain was cultivated in BG-11 medium^84^ at 20 °C, illuminated continuously by Sylvania GROLUX fluorescent lamps (50–200 μmol photons m^–2^ s^–1^), and aerated with 0.5–5% CO_2_ in filtered filtrated air in 20 L polycarbonate carboys. To minimize cell death and lysis, the cultures were harvested weekly and diluted with fresh medium (semicontinuous cultivation to avoid entry into the stationary phase). The biomass was subsequently lyophilized and stored at room temperature until further analysis.

### Extraction, Microfractionation and Isolation of Cyanobacterin and Its Analogues

A total of 43.0 g of lyophilized biomass was suspended in 50% MeOH (v/v) at a solvent-to-biomass ratio of 20 mL/g of dry biomass, homogenized by vortexing, treated with an ultrasonication rod (Bandelin, 2 min, amplitude 100%, power 50 W, no pulsation) and extracted on a shaker for 20 min. After centrifugation (4700 rpm at 20 °C, 10 min, Centrifuge 5415 C, Eppendorf), the biomass was subsequently extracted again with 50% MeOH (v/v) and in the same manner twice with 80% MeOH (v/v). The supernatants were combined and dried *in vacuo* using a centrifugal evaporator, resulting in 10.3 g of extract. Divided into seven portions of 1.5 g each, the extract was prepared as a dry load (support material Celite) and fractionated using flash chromatography on a C_18_ cartridge (CHROMABOND Flash RS 80 C_18_ec, 15–40 μm, 30.9 × 249 mm, Macherey Nagel). A step gradient of 20% (F1), 40% (F2), 60% (F3), 81% (F4) and 100% (F5) MeOH in water (v/v), each 600 mL, was used. Instead of the 80% step, 81% was chosen, as this ensured that all chlorinated SMs were collected in one fraction, which was subsequently dried *in vacuo* resulting in 337 mg.

Microfractionation and bioactivity testing were combined to identify potential PSII inhibitors. After reconstitution in 80% MeOH (v/v), 83 μg of F4 were used for microfractionation using the following parameters: Luna C18 column (250 × 4.6 mm, 5 μm, 100 Å, Phenomenex), binary gradient from 60 to 91% MeCN in H_2_O (0.1% formic acid each) at 1.3 mL/min in 20.1 min, stepping to 100% MeCN in 0.1 min, eluting at 100% MeCN for 3.8 min. Thirty microfractions were collected into a 96-deep-well-plate (every 0.5 min between 5 and 20 min).

The following chromatographic parameters were used for the isolation: Reconstituted F4 (50 mg/mL in 80% MeOH (v/v)) was subjected to semipreparative HPLC (Dionex UltiMate 3000, Thermo Scientific) using a Kinetex F5 column (250 × 10 mm, 5 μm, 100 Å, Phenomenex) and a binary gradient of 68–93% MeOH in water (0.1% formic acid each) for 31.5 min at 6.1 mL/min. The resulting compounds were (*Z*)-**1** (14.9 mg, t_R_ 24.5 min), (*E*/*Z*)-**2** (t_R_ 16.3 min), (*E*/*Z*)-**3** (0.7 mg, t_R_ 18.0 min), **4** (CB, 3.1 mg, t_R_ 19.3 min), (*E*/*Z*)-**5** (0.6 mg, t_R_ 28.1 min), **7** (0.9 mg, t_R_ 14.8 min), **8** (0.8 mg, t_R_ 26.3 min). The elution order of **2** and **7** as well as **5** and **8** varied depending on the use of MeOH or MeCN as the organic component of the gradient. For the separation of (*E*)-**2** and (*Z*)-**2**, the reconstituted *E*/*Z* mixture (50 mg/mL in 80% MeOH (v/v)) was further purified on a Kinetex C18 column (150 × 10 mm, 5 μm, 100 Å) using a binary gradient of 46–54% MeCN in water (0.1% formic acid each) over 25.0 min at 6.1 mL/min, affording (*E*)-**2** (0.7 mg, t_R_ 15.0 min) and (*Z*)-**2** (1.7 mg, t_R_ 17.4 min). Due to insufficient amounts of (*Z*)-6 in the previously mentioned batch, an additional 13.1 g of lyophilized biomass, harvested from an independent cultivation batch, was extracted to allow for complete structure elucidation. Fractionation was also performed using flash chromatography, albeit under the following conditions: a binary gradient of 5–100% MeCN in water (0.1% formic acid each) over 25.0 min, followed by isocratic conditions for an additional 5.0 min at 20.0 mL/min. A total of 20 fractions were collected, each spanning 1.5 min. F13, containing (*Z*)-**6** as the main compound, was further processed under the following chromatographic conditions: the reconstituted F13 (50 mg/mL in 80% MeOH (v/v)) was further purified on a Kinetex C18 column (150 × 10 mm, 5 μm, 100 Å) under nearly isocratic conditions (43–45% MeCN in water (0.1% formic acid each)) over 25.0 min at 6.1 mL/min, affording (*Z*)-**6** (1.3 mg, t_R_ 10.2 min).

Anhydrocyanobacterin (*E*/*Z*-**1**): pale yellow amorphous powder, UV (MeCN/H_2_O) λ_max_ 207 nm, 239 nm, 359 nm; ^1^H and ^13^C NMR see Table 1; HRESIMS *m*/*z* 413.1149 [M + H]^+^ (C_23_H_22_O_5_Cl, calc. 413.1150, Δ 0.2 ppm).

Anhydrocyanobacterin C ((*E*/*Z)*-**2**): pale yellow amorphous powder, UV (MeCN/H_2_O) λ_max_ 207 nm, 226 nm, 359 nm; ^1^H and ^13^C NMR see Table 1; HRESIMS *m*/*z* 385.1199 [M + H]^+^ (C_22_H_22_O_4_Cl, calc. 385.1201, Δ 0.5 ppm).

Anhydrocyanobacterin E ((*E*/*Z)*-**3**): pale yellow amorphous powder, UV (MeCN/H_2_O) λ_max_ 202 nm, 226 nm, 359 nm; ^1^H and ^13^C NMR see Table 1; HRESIMS *m*/*z* 415.1304 [M + H]^+^ (C_23_H_24_O_5_Cl, calc. 415.1307, Δ 0.7 ppm).

Cyanobacterin (**4**): white amorphous powder, UV (MeCN/H_2_O) λ_max_ 207 nm, 267 nm; ^1^H and ^13^C NMR see Table 3; HRESIMS *m*/*z* 429.1111 [M – H]^−^ (C_23_H_24_O_6_Cl, calc. 429.1110, Δ 0.2 ppm).

Anhydrocyanobacterin F ((*E*/*Z)*-**5**): pale yellow amorphous powder, UV (MeCN/H_2_O) λ_max_ 203 nm, 354 nm; ^1^H and ^13^C NMR see Table 2; HRESIMS *m*/*z* 447.0759 [M + H]^+^ (C_23_H_21_O_5_Cl_2_, calc. 447.0761, Δ 0.4 ppm).

Anhydrocyanobacterin B ((*Z)*-**6**): pale yellow amorphous powder, UV (MeCN/H_2_O) λ_max_ 203 nm, 232 nm, 359 nm; ^1^H and ^13^C NMR see Table 3; HRESIMS *m*/*z* 371.1042 [M + H]^+^ (C_21_H_20_O_4_Cl, calc. 371.1045, Δ 2.7 ppm).

Dihydroanhydrocyanobacterin D (**7**): white amorphous powder, UV (MeCN/H_2_O) λ_max_ 216 nm, 283 nm; ^1^H and ^13^C NMR see Table 3; HRESIMS *m*/*z* 401.1512 [M + H]^+^ (C_23_H_26_O_4_Cl, calc. 401.1514, Δ 0.5 ppm).

Bisanhydrocyanobacterin (**8**): white amorphous powder, UV (MeCN/H_2_O) λ_max_ 208 nm, 283 nm; ^1^H and ^13^C NMR see Table 3; HRESIMS *m*/*z* 825.2229 [M + H]^+^ (C_46_H_43_O_10_Cl_2_, calc. 825.2228, Δ 0.1 ppm).

### LC-MS/MS Data Acquisition

HRMS data acquisition was performed either on a Q Exactive Plus mass spectrometer (large scale cultures, purification) equipped with a heated ESI interface coupled to an UltiMate 3000 HPLC system or on an Orbitrap Exploris 240 mass spectrometer (small scale cultures, FBMN) equipped with a heated ESI interface coupled to a Vanquish Flex HPLC system (all Thermo Fisher Scientific). The following chromatographic parameters were used: Kinetex C18 column (50 × 2.1 mm, 2.6 μm, 100 Å, Phenomenex), binary gradient from 5 to 100% MeCN in H_2_O (0.1% formic acid each) at 0.4 mL/min in 16 min, 100% MeCN for 4 min. HRMS data acquisition: pos. and neg. ionization mode, ESI spray voltage 3.5 kV and −2.5 kV, capillary temperature 350 °C, sheath gas flow rate 50 L/min (A) or 40 L/min (B), auxiliary gas flow rate 12.5 L/min (Q Exactive Plus) or 5 L/min (Exploris 240). Full scan spectra were acquired from *m*/*z* 133.4 to 2000 with a resolution of 35000 at *m*/*z* 200, automated gain control (AGC) 5 × 10^5^, maximal injection time 120 ms. MS/MS spectra were acquired in data-dependent acquisition mode (dd-MS^2^), stepped collision energy of 30, 60, and 75 eV (resulting at 55 eV), a resolution of 17500 at *m*/*z* 200, an AGC of 2 × 10^5^, and a maximal injection time of 75 ms. A TopN experiment (N = 5, loop count 5) was implemented for triggering the dd-MS^2^ acquisition.

### File Conversion

Raw mass spectrometry data files were converted from .RAW to .mzML format using MSConvert from ProteoWizard (version 3.0).^85^ A scan polarity filter was used during data conversion to separate positive ion mode scans from negative ion mode scans, facilitating a more targeted analysis in the subsequent data analysis steps.

### MassQL Data Analysis

To identify chlorinated compounds, MassQL queries (Figure S3) were designed based on the distinctive isotope pattern of chlorine (^35^Cl/^37^Cl), taking into account varying degrees of chlorination. The queries targeted specific MS1 peaks at *m*/*z* values corresponding to the isotope pattern of chlorine (e.g., *m*/*z* X + 2.0, X + 4.0, etc.), with relative intensity thresholds, based on calculations performed using the enviPat isotope pattern calculator.^55^ The *m*/*z* tolerance and allowed relative intensity variability were chosen based on the requirements and characteristics of the isotope pattern for each degree of chlorination (mono- to hexa-Cl). MS^2^ spectra were also queried to confirm the presence of precursor ions. For brominated SMs, MassQL queries (Figure S4) were used as previously reported for the polybrominated analogue of aetokthonotoxin.^47,56^ The query results were filtered for a minimum intensity of 1E6 for further analysis.

### Classical and Feature Based Molecular Networking

To facilitate FBMN analysis, the converted mass spectrometry data were processed using MZmine (versions 3 and 4.2) with a workflow designed and executed through the MZWizard tool to automate the feature extraction and alignment.^86^ For mass detection, the following parameters were used: noise level thresholds of 5.00 (MS) and 0.00 (MS^2^), which discarded low-intensity signals. Chromatogram building was performed with the following parameters: minimum consecutive scans 4, minimum absolute height 1.0E5, *m*/*z* tolerance 10 ppm.^87^ Chromatographic peaks were smoothed using a Savitzky-Golay filter (window size 5 points) to reduce noise and refine the peak shape. The Join Aligner module for peak alignment was used with the following parameters: retention time tolerance 0.4 min, *m*/*z* tolerance 5 ppm. The molecular networks for CMN and FBMN analysis were generated using GNPS (http://gnps.ucsd.edu).^48^ All HRMS^2^ fragment ions within a 17 Da window of the precursor *m*/*z* were excluded from the data set. HRMS^2^ spectra were further filtered to select the six most prominent fragment ions within a 50 Da window. Precursor ion mass tolerance was set to 0.02 Da, and the same tolerance was applied to HRMS^2^ fragment ions. Networks were constructed by retaining edges with a cosine score >0.7 and a minimum of four matched peaks. Edges were retained only if both nodes appeared in each other’s top ten most similar nodes list. The maximum size of any molecular family was limited to 100, and the lowest scoring edges were removed until each molecular family was below this threshold.^48,49^ The resulting molecular networks were visualized and analyzed using Cytoscape (version 3.10.2).^88,89^ GNPS output, including network data (in GraphML format), was imported into Cytoscape for interactive visualization.

### Light-Induced Dimerization of **1**

**1** was dissolved in DMSO (4 mg/mL). DMSO was chosen to avoid evaporation during the experimental period. The resulting solution was exposed to light at a wavelength of 254 nm in an open Petri dish. Samples were taken at t = 0, 30, 60 min and then every 60 min for a total of 420 min and analyzed by HPLC-HRMS.

### Assay for Herbicidal Effect

Liquid cultures of *Synechocystis* sp. PCC6803 were grown in BG-11 medium in 250 mL Erlenmeyer flasks at 25 °C under constant white light of 50 μmol photons m^–2^ s^–1^, with orbital shaking at 40 rpm until an OD_750_ of 1.0 was reached. Immediately before the start of the bioactivity assay, cultures were diluted with fresh BG-11 medium to an OD_750_ of 0.15 and split into multiple aliquots of 2 mL. Vacuum-dried microfractions were resupended in 1 mL DMSO of which 2 μL were added to individual aliquots in duplicates and distributed to 12-well plates (Greiner Cellstar, Cat.No. 665 102). Plates were incubated under the same conditions as before, with light intensities of 12 μmol photons m^–2^ s^–1^ and grown for 14 days.

### Quantification by Evaporative Light Scattering Detection (ELSD)

To avoid weighing inaccuracies, the concentrations of test compound solutions for bioactivity testing were quantified using HPLC coupled with an evaporative light scattering detector (1290 Infinity II, Agilent) as described previously.^90^ Synthetic aetokthonotoxin^51^ was used as standard substance to establish a calibration curve (triplicate injection of 1 to 10 μL of a 23,7 ng/μL solution in 90% MeCN) on a Kinetex C18 column (100 × 3 mm, 2.6 μm, 100 Å, Phenomenex), eluted with a gradient from 10 to 100% MeCN in H_2_O (0.1% FA each) over 10 min at 0.65 mL/min. Settings of the ELSD were as follows: evaporator temperature 45 °C, nebulizer temperature 45 °C, gas flow rate 1.3 SLM, N_2_ 3.5 bar. The calibration curve was generated as described by Young et al.^91^ In brief, the response areas were averaged, and log­(ELSD response area) was plotted against log­(amount in ng) to generate a linear calibration curve. Compounds **1**, **4**, and **7** were dissolved in 1 mL MeCN 90% (v/v), diluted 1:3 in the same solvent and injected in triplicate under the same conditions.

### Cell Culture and Cytotoxicity Testing

HCT116 cells were maintained in a humidified atmosphere at 37 °C with 5% CO_2_. HCT116 cells were kept in McCoy’s 5A medium (Thermo Fisher scientific) supplied with 10% fetal bovine serum (Sigma-Aldrich) and penicillin (10000 U/L)/streptomycin (100 mg/L) (Roth). The sulforhodamine B (SRB) colorimetric assay was conducted as previously described by Vichai et al.^92^ Briefly, 2 × 10^4^ cells per well were seeded in a clear, cell culture treated 96 well plate with flat bottom (Greiner). The following day, cells were incubated for 24 h with different concentrations of compounds **1**, **4**, and **7**, or solvent control: 0.1% DMSO. After the incubation time, cells were directly fixed with cold 10% (w/v) trichloroacetic acid (Roth) for 1 h. Then, cells were carefully rinsed four times with slow-running deionized tap water and blow dried. When cells were completely dry, 0.057% (w/v) of SRB solution (Sigma-Aldrich) in 1% (v/v) acetic acid (ITW Reagents) was added to the wells. After 30 min incubation at room temperature, cells were quickly washed four times with 1% (v/v) acetic acid and blow dried. Finally, 10 mM Tris base solution (pH 10.5) was added to the completely dry wells. The plate was placed in a TECAN Infinite M Plex plate reader, orbitally shaken for 300 s, and absorbance was recorded at 510 nm. The experiment was performed in three independent biological replicates. Results are presented as treatment over control in percentage. Statistical analysis of the data was conducted with Origin 2021b (OriginLab Corporation). After assessing normal distribution with Lillifors test, Mann–Whitney test was used to evaluate the differences between treatment and control.

## Supplementary Material



## Data Availability

NMR raw data have been archived at 10.57992/nmrxiv.p112.

## Notes

Additional information about the MassQL queries, FBMN, structures, HRMS, MS/MS, UV/vis spectra of all described compounds; ^1^H, ^13^C, HSQC, HMBC, and COSY spectra of compounds **1**–**8** (PDF)

## References

[1] Dittmann E., Gugger M., Sivonen K., Fewer D. P. (2015). Natural Product
Biosynthetic Diversity and Comparative Genomics of the Cyanobacteria. Trends Microbiol..

[2] Demay J., Bernard C., Reinhardt A., Marie B. (2019). Natural Products from
Cyanobacteria: Focus on Beneficial Activities. Mar. Drugs.

[3] Niedermeyer T. H. J. (2015). Anti-infective
Natural Products from Cyanobacteria. Planta
Med..

[4] Gribble G. W. (2024). A Survey
of Recently Discovered Naturally Occurring Organohalogen Compounds. J. Nat. Prod..

[5] Jones M. R., Pinto E., Torres M. A., Dorr F., Mazur-Marzec H., Szubert K., Tartaglione L., Dell'Aversano C., Miles C. O., Beach D. G., McCarron P., Sivonen K., Fewer D. P., Jokela J., Janssen E. M.-L. (2021). CyanoMetDB,
a
comprehensive public database of secondary metabolites from cyanobacteria. Water Res..

[6] Janssen, E. M.-L. ; Jones, M. R. ; Pinto, E. ; Dörr, F. ; Torres, M. A. ; Rios Jacinavicius, F. ; Mazur-Marzec, H. ; Szubert, K. ; Konkel, R. ; Tartaglione, L. ; . S75 | CyanoMetDB | Comprehensive database of secondary metabolites from cyanobacteria. NORMAN-SLE-S75.0.3.0 ed.; Zenodo: 2024.

[7] Murakami M., Ishida K., Okino T., Okita Y., Matsuda H., Yamaguchi K. (1995). Aeruginosins 98-A and B, trypsin inhibitors from the
blue-green alga *Microcystis aeruginosa* (NIES-98). Tetrahedron Lett..

[8] Matern U., Oberer L., Falchetto R. A., Erhard M., König W. A., Herdman M., Weckesser J. (2001). Scyptolin
A and B, cyclic depsipeptides
from axenic cultures of *Scytonema hofmanni* PCC 7110. Phytochemistry.

[9] Ishida K., Matsuda H., Murakami M. (1998). Four new microginins,
linear peptides
from the cyanobacterium *Microcystis aeruginosa*. Tetrahedron.

[10] Golakoti T., Ogino J., Heltzel C. E., Le Husebo T., Jensen C. M., Larsen L. K., Patterson G. M. L., Moore R. E., Mooberry S. L., Corbett T. H. (1995). Structure
determination, conformational analysis, chemical stability studies,
and antitumor evaluation of the cryptophycins. Isolation of 18 new
analogs from *Nostoc* sp. strain GSV 224. J. Am. Chem. Soc..

[11] Luesch H., Yoshida W. Y., Moore R. E., Paul V. J., Mooberry S. L. (2000). Isolation,
Structure Determination, and Biological Activity of Lyngbyabellin
A from the Marine Cyanobacterium *Lyngbya majuscula*. J. Nat. Prod..

[12] Milligan K. E., Marquez B. L., Williamson R. T., Gerwick W. H. (2000). Lyngbyabellin B,
a Toxic and Antifungal Secondary Metabolite from the Marine Cyanobacterium *Lyngbya majuscula*. J. Nat. Prod..

[13] Han B., McPhail K. L., Gross H., Goeger D. E., Mooberry S. L., Gerwick W. H. (2005). Isolation and structure
of five lyngbyabellin derivatives
from a Papua New Guinea collection of the marine cyanobacterium *Lyngbya majuscula*. Tetrahedron.

[14] Chilczuk T., Steinborn C., Breinlinger S., Zimmermann-Klemd A. M., Huber R., Enke H., Enke D., Niedermeyer T. H. J., Grundemann C. (2020). Hapalindoles from the Cyanobacterium *Hapalosiphon* sp. Inhibit T Cell Proliferation. Planta Med..

[15] Moore R. E., Cheuk C., Patterson G. M. L. (1984). Hapalindoles: new alkaloids from
the blue-green alga *Hapalosiphon fontinalis*. J. Am. Chem. Soc..

[16] Klein D., Daloze D., Braekman J. C., Hoffmann L., Demoulin V. (1995). New Hapalindoles
from the Cyanophyte *Hapalosiphon laingii*. J. Nat. Prod..

[17] Becher P. G., Keller S., Jung G., Süssmuth R. D., Jüttner F. (2007). Insecticidal activity of 12-epi-hapalindole
J isonitrile. Phytochemistry.

[18] Stratmann K., Moore R. E., Bonjouklian R., Deeter J. B., Patterson G. M. L., Shaffer S., Smith C. D., Smitka T. A. (1994). Welwitindolinones,
Unusual Alkaloids from the Blue-Green Algae *Hapalosiphon welwitschii* and *Westiella intricata*. Relationship to Fischerindoles
and Hapalinodoles. J. Am. Chem. Soc..

[19] Park A., Moore R. E., Patterson G. M. L. (1992). Fischerindole L, a new isonitrile
from the terrestrial blue-green alga *Fischerella muscicola*. Tetrahedron Lett..

[20] Smitka T. A., Bonjouklian R., Doolin L., Jones N. D., Deeter J. B., Yoshida W. Y., Prinsep M. R., Moore R. E., Patterson G. M. L. (1992). Ambiguine
isonitriles, fungicidal hapalindole-type alkaloids from three genera
of blue-green algae belonging to the *Stigonemataceae*. J. Org. Chem..

[21] Chilczuk T., Monson R., Schmieder P., Christov V., Enke H., Salmond G., Niedermeyer T. H. J. (2020). Ambigols
from the Cyanobacterium
Fischerella ambigua Increase Prodigiosin Production in *Serratia* spp. ACS Chem. Biol..

[22] Falch B. S., Koenig G. M., Wright A. D., Sticher O., Ruegger H., Bernardinelli G. (1993). Ambigol A
and B: new biologically active polychlorinated
aromatic compounds from the terrestrial blue-green alga *Fischerella
ambigua*. J. Org. Chem..

[23] Chilczuk T., Schaberle T. F., Vahdati S., Mettal U., El Omari M., Enke H., Wiese M., Konig G. M., Niedermeyer T. H. J. (2020). Halogenation-Guided
Chemical Screening Provides Insight into Tjipanazole Biosynthesis
by the Cyanobacterium *Fischerella ambigua*. ChemBioChem..

[24] Bonjouklian R., Smitka T. A., Doolin L. E., Molloy R. M., Debono M., Shaffer S. A., Moore R. E., Stewart J. B., Patterson G. M. L. (1991). Tjipanazoles,
new antifungal agents from the blue-green alga *Tolypothrix
tjipanasensis*. Tetrahedron.

[25] Cardellina J. H., Marner F. J., Moore R. E. (1979). Malyngamide A, a
novel chlorinated metabolite of the marine cyanophyte *Lyngbya
majuscula*. J. Am. Chem. Soc..

[26] Bui H. T. N., Jansen R., Pham H. T. L., Mundt S. (2007). Carbamidocyclophanes
A–E, Chlorinated Paracyclophanes with Cytotoxic and Antibiotic
Activity from the Vietnamese Cyanobacterium *Nostoc* sp. J. Nat. Prod..

[27] Dai J., Philbin C. S., Wakano C., Yoshida W. Y., Williams P. G. (2023). New Nostocyclophanes
from *Nostoc linckia*. Mar. Drugs.

[28] Preisitsch M., Harmrolfs K., Pham H. T. L., Heiden S. E., Füssel A., Wiesner C., Pretsch A., Swiatecka-Hagenbruch M., Niedermeyer T. H. J., Müller R. (2015). Anti-MRSA-acting carbamidocyclophanes
H–L from the Vietnamese cyanobacterium *Nostoc* sp. CAVN2. J. Antibiot..

[29] Leao P. N., Nakamura H., Costa M., Pereira A. R., Martins R., Vasconcelos V., Gerwick W. H., Balskus E. P. (2015). Biosynthesis-assisted
structural elucidation of the bartolosides, chlorinated aromatic glycolipids
from cyanobacteria. Angew. Chem., Int. Ed. Engl..

[30] Kim G. J., Mascuch S. J., Mevers E., Boudreau P. D., Gerwick W. H., Choi H. (2022). Luquilloamides,
Cytotoxic Lipopeptides from a Puerto Rican Collection
of the Filamentous Marine Cyanobacterium *Oscillatoria* sp. J. Org. Chem..

[31] Taguchi R., Iwasaki A., Ebihara A., Jeelani G., Nozaki T., Suenaga K. (2022). Isolation and Total
Synthesis of Beru’amide,
an Antitrypanosomal Polyketide from a Marine Cyanobacterium *Okeania* sp. Org. Lett..

[32] Brumley D. A., Gunasekera S. P., Chen Q.-Y., Paul V. J., Luesch H. (2020). Discovery,
Total Synthesis, and SAR of Anaenamides A and B: Anticancer Cyanobacterial
Depsipeptides with a Chlorinated Pharmacophore. Org. Lett..

[33] Mason C. P., Edwards K. R., Carlson R. E., Pignatello J., Gleason F. K., Wood J. M. (1982). Isolation of Chlorine-Containing
Antibiotic From the Freshwater Cyanobacterium *Scytonema hofmanni*. Science.

[34] Gleason F. K., Baxa C. A. (1986). Activity of the
natural algicide, cyanobacterin, on
eukaryotic microorganisms. FEMS Microbiol. Lett..

[35] Gleason F. K., Paulson J. L. (1984). Site of action of the natural algicide, cyanobacterin,
in the blue-green alga, *Synechococcus* sp. Arch. Microbiol..

[36] Jong T. T., Williard P. G., Porwoll J. P. (1984). Total synthesis
and x-ray structure
determination of cyanobacterin. J. Org. Chem..

[37] Gleason, F. K. ; Thoma, W. J. ; Carlson, J. L. Cyanobacterin and Analogs: Structure and Activity Relationships of a Natural Herbicide. In Progress in Photosynthesis Research: Volume 3 Proceedings of the VIIth International Congress on Photosynthesis Providence, Rhode Island, USA, August 10–15, 1986; Biggins, J. , Ed.; Springer: Dordrecht, The Netherlands, 1987; pp 763–766.

[38] Pignatello J. J., Porwoll J., Carlson R. E., Xavier A., Gleason F. K., Wood J. M. (1983). Structure of the antibiotic cyanobacterin,
a chlorine-containing.gamma.-lactone
from the freshwater cyanobacterium *Scytonema hofmanni*. J. Org. Chem..

[39] Gleason F. K., Porwoll J., Flippen-Anderson J. L., George C. (1986). X-ray structure determination
of the naturally occurring isomer of cyanobacterin. Journal of Organic Chemistry.

[40] Carlson, J. L. ; Leaf, T. A. ; Gleason, F. K. Synthesis and Activity of Analogs of the Natural Herbicide Cyanobacterin. In Synthesis and Chemistry of Agrochemicals; ACS Symposium Series; American Chemical Society: 1987; 355; pp 141–150.

[41] Gleason F. K., Case D. E. (1986). Activity of the Natural Algicide, Cyanobacterin, on
Angiosperms. Plant Physiol..

[42] Gleason F. K., Case D. E., Sipprell K. D., Magnuson T. S. (1986). Effect of the natural
algicide, cyanobacterin, on a herbicide-resistant mutant of *anacystis nidulans* R2. Plant Sci..

[43] Gleason F. K. (1990). The natural
herbicide, cyanobacterin, specifically disrupts thylakoid membrane
structure in *Euglena gracilis* strain Z. FEMS Microbiol. Lett..

[44] Thoma W. J., Gleason F. K. (1987). In vivo effects
of photosynthesis inhibitors in Synechococcus
as determined by phosphorus-31 NMR spectroscopy. Biochemistry.

[45] Jarmusch S. A., van der Hooft J. J. J., Dorrestein P. C., Jarmusch A. K. (2021). Advancements in
capturing and mining mass spectrometry data are transforming natural
products research. Nat. Prod. Rep..

[46] Léon A., Cariou R., Hutinet S., Hurel J., Guitton Y., Tixier C., Munschy C., Antignac J.-P., Dervilly-Pinel G., Le Bizec B. (2019). HaloSeeker 1.0: A User-Friendly Software to Highlight
Halogenated Chemicals in Nontargeted High-Resolution Mass Spectrometry
Data Sets. Anal. Chem..

[47] Damiani T., Jarmusch A. K., Aron A. T., Petras D., Phelan V. V., Zhao H. N., Bittremieux W., Acharya D. D., Ahmed M. M. A., Bauermeister A. (2025). A universal language for finding mass
spectrometry data patterns. Nat. Methods.

[48] Wang M., Carver J. J., Phelan V. V., Sanchez L. M., Garg N., Peng Y., Nguyen D. D., Watrous J., Kapono C. A., Luzzatto-Knaan T. (2016). Sharing and community curation of mass spectrometry
data with Global Natural Products Social Molecular Networking. Nat. Biotechnol..

[49] Nothias L.-F., Petras D., Schmid R., Dührkop K., Rainer J., Sarvepalli A., Protsyuk I., Ernst M., Tsugawa H., Fleischauer M. (2020). Feature-based molecular
networking in the GNPS analysis environment. Nat. Methods.

[50] Preisitsch M., Heiden S. E., Beerbaum M., Niedermeyer T. H. J., Schneefeld M., Herrmann J., Kumpfmüller J., Thürmer A., Neidhardt I., Wiesner C. (2016). Effects
of Halide Ions on the Carbamidocyclophane Biosynthesis in Nostoc sp.
CAVN2. Mar. Drugs.

[51] Ricardo M. G., Schwark M., Llanes D., Niedermeyer T. H. J., Westermann B. (2021). Total Synthesis of Aetokthonotoxin,
the Cyanobacterial
Neurotoxin Causing Vacuolar Myelinopathy. Chemistry–A
European Journal.

[52] Breinlinger S., Phillips T. J., Haram B. N., Mareš J., Martínez Yerena J. A., Hrouzek P., Sobotka R., Henderson W. M., Schmieder P., Williams S. M. (2021). Hunting
the eagle killer: A cyanobacterial neurotoxin causes vacuolar myelinopathy. Science.

[53] Leon A., Cariou R., Hutinet S., Hurel J., Guitton Y., Tixier C., Munschy C., Antignac J. P., Dervilly-Pinel G., Le Bizec B. (2019). HaloSeeker 1.0: A User-Friendly
Software to Highlight
Halogenated Chemicals in Nontargeted High-Resolution Mass Spectrometry
Data Sets. Anal. Chem..

[54] Flon V., Pavesi C., Oger S., Leleu S., Retailleau P., Jennings L. K., Prado S., Franck X. (2025). Mass-tagged aminated
probes for rapid discovery of azaphilic natural products in fungal
crude extracts. Chem. Commun..

[55] Loos M., Gerber C., Corona F., Hollender J., Singer H. (2015). Accelerated Isotope Fine Structure
Calculation Using
Pruned Transition Trees. Anal. Chem..

[56] Schanbacher F., Rebhahn V. I. C., Schwark M., Breinlinger S., Štenclová L., Röhrborn K., Schmieder P., Enke H., Wilde S. B., Niedermeyer T. H. J. (2025). Mining
for Halogenated Metabolites of Aetokthonos hydrillicola, the “Eagle
Killer” Cyanobacterium. J. Nat. Prod..

[57] Lee E.-S. J., Gleason F. K. (1994). A second
algicidal natural product from the cyanobacterium. Scytonema hofmanni. Plant Sci..

[58] D’Agostino P.
M., Seel C. J., Ji X., Gulder T., Gulder T. A. M. (2022). Biosynthesis
of cyanobacterin, a paradigm for furanolide core structure assembly. Nat. Chem. Biol..

[59] Felder S., Kehraus S., Neu E., Bierbaum G., Schäberle T. F., König G. M. (2013). Salimyxins
and Enhygrolides: Antibiotic, Sponge-Related
Metabolites from the Obligate Marine *Myxobacterium Enhygromyxa* salina. ChemBioChem..

[60] Kossack R., Breinlinger S., Nguyen T., Moschny J., Straetener J., Berscheid A., Brotz-Oesterhelt H., Enke H., Schirmeister T., Niedermeyer T. H. J. (2020). Nostotrebin 6 Related Cyclopentenediones and delta-Lactones
with Broad Activity Spectrum Isolated from the Cultivation Medium
of the Cyanobacterium Nostoc sp. CBT1153. J.
Nat. Prod..

[61] Davis R. A., Carroll A. R., Duffy S., Avery V. M., Guymer G. P., Forster P. I., Quinn R. J. (2007). Endiandrin A, a
Potent Glucocorticoid
Receptor Binder Isolated from the Australian Plant *Endiandra
anthropophagorum*. J. Nat. Prod..

[62] Davis R. A., Barnes E. C., Longden J., Avery V. M., Healy P. C. (2009). Isolation,
structure elucidation and cytotoxic evaluation of endiandrin B from
the Australian rainforest plant *Endiandra anthropophagorum*. Biorg. Med. Chem..

[63] Yang P., Jia Q., Song S., Huang X. (2023). [2 + 2]-Cycloaddition-derived cyclobutane
natural products: structural diversity, sources, bioactivities, and
biomimetic syntheses. Nat. Prod. Rep..

[64] Assmann M., Köck M. (2002). Bromosceptrin,
an Alkaloid from the Marine Sponge *Agelas conifera*. Zeitschrift für
Naturforschung C.

[65] Sun Y.-T., Lin B., Li S.-G., Liu M., Zhou Y.-J., Xu Y., Hua H.-M., Lin H.-W. (2017). New bromopyrrole
alkaloids from the
marine sponge *Agelas* sp. Tetrahedron.

[66] Ankisetty S., Nandiraju S., Win H., Park Y. C., Amsler C. D., McClintock J. B., Baker J. A., Diyabalanage T. K., Pasaribu A., Singh M. P. (2004). Chemical Investigation
of Predator-Deterred Macroalgae from the Antarctic Peninsula. J. Nat. Prod..

[67] Long H., Cheng Z., Huang W., Wu Q., Li X., Cui J., Proksch P., Lin W. (2016). Diasteltoxins
A–C, Asteltoxin-Based
Dimers from a Mutant of the Sponge-Associated *Emericella variecolor* Fungus. Org. Lett..

[68] Cao F., Meng Z.-H., Wang P., Luo D.-Q., Zhu H.-J. (2020). Dipleosporalones
A and B, Dimeric Azaphilones from a Marine-Derived *Pleosporales* sp. Fungus. J. Nat. Prod..

[69] Gutekunst W. R., Baran P. S. (2011). Total Synthesis
and Structural Revision of the Piperarborenines
via Sequential Cyclobutane C–H Arylation. J. Am. Chem. Soc..

[70] Li J., Gao K., Bian M., Ding H. (2020). Recent advances in the total synthesis
of cyclobutane-containing natural products. Org. Chem. Front..

[71] Kleks G., Duffy S., Lucantoni L., Avery V. M., Carroll A. R. (2020). Orthoscuticellines
A–E, β-Carboline Alkaloids from the Bryozoan *Orthoscuticella ventricosa* Collected in Australia. J. Nat. Prod..

[72] Skiredj A., Beniddir M. A., Joseph D., Leblanc K., Bernadat G., Evanno L., Poupon E. (2014). Spontaneous
Biomimetic Formation
of (±)-Dictazole B under Irradiation with Artificial Sunlight. Angew. Chem., Int. Ed..

[73] Beniddir M. A., Evanno L., Joseph D., Skiredj A., Poupon E. (2016). Emergence
of diversity and stereochemical outcomes in the biosynthetic pathways
of cyclobutane-centered marine alkaloid dimers. Nat. Prod. Rep..

[74] Yang X., Shimizu Y., Steiner J. R., Clardy J. (1993). Nostoclide I and II,
extracellular metabolites from a symbiotic cyanobacterium, *Nostoc* sp., from the lichen *Peltigera canina*. Tetrahedron Lett..

[75] Teixeira R. R., Barbosa L. C. A., Forlani G., Piló-Veloso D., Walkimar de Mesquita Carneiro J. (2008). Synthesis
of Photosynthesis-Inhibiting
Nostoclide Analogues. J. Agric. Food Chem..

[76] Barbosa L.
C., Demuner A. J., de Alvarenga E. S., Oliveira A., King-Diaz B., Lotina-Hennsen B. (2006). Phytogrowth- and photosynthesis-inhibiting properties
of nostoclide analogues. Pest Manage. Sci..

[77] Teixeira R.
R., Barbosa L. C. A., Santana J. O., Veloso D. P., Ellena J., Doriguetto A. C., Drew M. G. B., Ismail F. M. D. (2007). Synthesis and
structural characterization of two nostoclide analogues. J. Mol. Struct..

[78] Xu H.-W., Wang J.-F., Liu G.-Z., Hong G.-F., Liu H.-M. (2007). Facile
synthesis of γ-alkylidenebutenolides. Org. Biomol. Chem..

[79] Raju R., Garcia R., Müller R. (2014). Angiolactone,
a new Butyrolactone
isolated from the terrestrial myxobacterium, *Angiococcus* sp. Journal of Antibiotics.

[80] Karak M., Acosta J. A. M., Cortez-Hernandez H. F., Cardona J. L., Forlani G., Barbosa L. C. A. (2024). Natural Rubrolides
and Their Synthetic Congeners as
Inhibitors of the Photosynthetic Electron Transport Chain. J. Nat. Prod..

[81] Pereira U. A., Barbosa L. C. A., Demuner A. J., Silva A. A., Bertazzini M., Forlani G. (2015). Rubrolides as Model
for the Development of New Lactones
and Their Aza Analogs as Potential Photosynthesis Inhibitors. Chem. Biodivers..

[82] Pearce A. N., Chia E. W., Berridge M. V., Maas E. W., Page M. J., Webb V. L., Harper J. L., Copp B. R. (2007). E/Z-Rubrolide O,
an Anti-inflammatory Halogenated Furanone from the New Zealand Ascidian *Synoicum* n. sp. J. Nat. Prod..

[83] Wu H., Lv G., Liu L., Hu R., Zhao F., Song M., Zhang S., Fan H., Dai S., Rehman S. u. (2024). Synthesis, Biological Evaluation, and
Mechanistic Insights of Rubrolide
Analogues as Antitumor Agents. J. Nat. Prod..

[84] Andersen, R. Algal Culturing Techniques; Academic Press: 2005. 10.1016/B978-012088426-1/50007-X.

[85] Chambers M. C., Maclean B., Burke R., Amodei D., Ruderman D. L., Neumann S., Gatto L., Fischer B., Pratt B., Egertson J. (2012). A cross-platform toolkit for mass spectrometry
and proteomics. Nat. Biotechnol..

[86] Schmid R., Heuckeroth S., Korf A., Smirnov A., Myers O., Dyrlund T. S., Bushuiev R., Murray K. J., Hoffmann N., Lu M. (2023). Integrative analysis of multimodal mass spectrometry
data in MZmine 3. Nat. Biotechnol..

[87] Myers O. D., Sumner S. J., Li S., Barnes S., Du X. (2017). One Step Forward
for Reducing False Positive and False Negative Compound Identifications
from Mass Spectrometry Metabolomics Data: New Algorithms for Constructing
Extracted Ion Chromatograms and Detecting Chromatographic Peaks. Anal. Chem..

[88] Shannon P., Markiel A., Ozier O., Baliga N. S., Wang J. T., Ramage D., Amin N., Schwikowski B., Ideker T. (2003). Cytoscape: a software environment
for integrated models
of biomolecular interaction networks. Genome
Res..

[89] Saito R., Smoot M. E., Ono K., Ruscheinski J., Wang P. L., Lotia S., Pico A. R., Bader G. D., Ideker T. (2012). A travel guide to Cytoscape plugins. Nat. Methods.

[90] Adnani N., Michel C. R., Bugni T. S. (2012). Universal
quantification of structurally
diverse natural products using an evaporative light scattering detector. J. Nat. Prod..

[91] Young C. S., Dolan J. W. (2003). Success with Evaporative
Light-Scattering Detection. LCGC North America.

[92] Vichai V., Kirtikara K. (2006). Sulforhodamine
B colorimetric assay for cytotoxicity
screening. Nat. Protoc..

