# Supplementary material for: Halogenation-Guided Chemical Screening Uncovers Cyanobacterin Analogues from the Cyanobacterium Tolypothrix sp. PCC9009
Source: J Nat Prod. 2025 Sep 12;88(9):2076–89. doi: 10.1021/acs.jnatprod.5c00591 (PMC12481558; doi:10.1021/acs.jnatprod.5c00591)
Supplement: Supplementary file 1 [file np5c00591_si_001.pdf]

Supporting Information

Halogenation-Guided Chemical Screening Uncovers Cyanobacterin Analogues from the Cyanobacterium *Tolypothrix* sp. PCC9009

*Franziska Schanbacher[1,†], Arthur Guljamow[2], Valerie I. C. Rebhahn[1], Peter Schmieder[3], Heike Enke[4], Elke Dittmann[2], Martin Baunach[5], Timo H. J. Niedermeyer[1,†]**

[1]Department of Pharmaceutical Biology, Institute of Pharmacy, Freie Universität Berlin, 14195 Berlin, Germany
[2] Institut for Biochemistry and Biology, University of Potsdam, 14476 Potsdam, Germany
[3]Leibniz-Forschungsinstitut für Molekulare Pharmakologie, Department of NMR-Supported Structural Biology, 13125 Berlin, Germany
[4]Simris Biologics GmbH, 12489 Berlin, Germany
[5]Institute of Pharmaceutical Biology, University of Bonn, 53115 Bonn, German

[†]Part of this work was conducted at the Department of Pharmaceutical Biology/Pharmacognosy, Institute of Pharmacy, Martin-Luther-University Halle-Wittenberg, 06120 Halle (Saale), Germany

*Corresponding author timo.niedermeyer@fu-berlin.de

List of Figures

Figure S1. First MassQL query to detect chlorinated specialized metabolites in the dataset. 5
Figure S2. Calculation **(1)** to estimate the ^{13}C X+2 peak intensity 6
Figure S3. Optimized MassQL query to detect chlorinated specialized metabolites in the dataset............ 8
Figure S4. MassQL queries to detect brominated specialized metabolites in the dataset........................... 9
Figure S5. Microfractionation and results from the assays for herbicidal activity 10
Figure S6. UV spectra 11
Figure S7. HRMS data of compound (*E*/*Z*) **1**. 12
Figure S8. HRMS data of compound **2**. 13
Figure S9. HRMS data of compound (*E*/*Z*)-**3**. 15
Figure S10. HRMS data of compound **4**. 16
Figure S11. HRMS data of compound (*E*/*Z*)-**5**. 17
Figure S12. HRMS data of compound (*Z*)-**6**. 18
Figure S13. HRMS data of compound **7**. 19
Figure S14. HRMS data of compound **8**. 20
Figure S15. Dimer formation (*E*/*Z*)-**1**. 21
Figure S16. ^{1}H NMR spectrum (600 MHz) of compound (*E*/*Z*)-**1** in DMSO-d_6. 22
Figure S17. ^{13}C NMR spectrum (150 MHz) of compound (*E*/*Z*)-**1** in DMSO-d_6. 23
Figure S18. COSY NMR spectrum (600 MHz) of compound (*E*/*Z*)-**1** in DMSO-d_6. 24
Figure S19. ^{13}C-HMQC NMR spectrum (600 MHz) of compound (*E*/*Z*)-**1** in DMSO-d_6. 25
Figure S20. ^{13}C-HMBC NMR spectrum (600 MHz) of compound (*E*/*Z*)- **1** in DMSO-d_6. 26
Figure S21. NOESY NMR spectrum (600 MHz) of compound (*E*/*Z*)- **1** in DMSO-d_6. 27
Figure S22. ^{1}H NMR spectrum (600 MHz) of compound (*Z*)-**2** in DMSO-d_6. 28
Figure S23. ^{13}C NMR spectrum (150 MHz) of compound (*Z*)-**2** in DMSO-d_6. 29
Figure S24. COSY NMR spectrum (600 MHz) of compound (*Z*)-**2** in DMSO-d_6. 30
Figure S25. ^{13}C-HMQC NMR spectrum (600 MHz) of compound (*Z*)-**2** in DMSO-d_6. 31
Figure S26. ^{13}C-HMBC NMR spectrum (600 MHz) of compound (*Z*)-**2** in DMSO-d_6. 32
Figure S27. NOESY NMR spectrum (600 MHz) of compound (*Z*)-**2** in DMSO-d_6. 33
Figure S28. ^{1}H NMR spectrum (600 MHz) of compound (*E*)-**2** in DMSO-d_6. 34
Figure S29. ^{13}C NMR spectrum (150 MHz) of compound (*E*)-**2** in DMSO-d_6. 35
Figure S30. COSY NMR spectrum (600 MHz) of compound (*E*)-**2** in DMSO-d_6. 36
Figure S31. ^{13}C-HMQC NMR spectrum (600 MHz) of compound (*E*)-**2** in DMSO-d_6. 37
Figure S32. ^{13}C-HMBC NMR spectrum (600 MHz) of compound (*E*)-**2** in DMSO-d_6. 38

Figure S33. ROESY NMR spectrum (600 MHz) of compound (*E*)-**2** in DMSO-d_6. 39
Figure S34. ^{1}H NMR spectrum (600 MHz) of compound (*Z*/*E*)-**3** in DMSO-d_6. 40
Figure S35. ^{13}C NMR spectrum (150 MHz) of compound (*E*/*Z*)-**3** in DMSO-d_6. 41
Figure S36. COSY NMR spectrum (600 MHz) of compound (*E*/*Z*)-**3** in DMSO-d_6. 42
Figure S37. ^{13}C-HMQC NMR spectrum (600 MHz) of compound (*E*/*Z*)-**3** in DMSO-d_6. 43
Figure S38. ^{13}C-HMBC NMR spectrum (600 MHz) of compound (*E*/*Z*)-**3** in DMSO-d_6. 44
Figure S39. NOESY NMR spectrum (600 MHz) of compound (*E*/*Z*)-**3** in DMSO-d_6. 45
Figure S40. ^{1}H NMR spectrum (600 MHz) of compound **4** in DMSO-d_6. 46
Figure S41. ^{13}C NMR spectrum (150 MHz) of compound **4** in DMSO-d_6. 47
Figure S42. COSY NMR spectrum (600 MHz) of compound **4** in DMSO-d_6. 48
Figure S43. ^{13}C-HSQC NMR spectrum (600 MHz) of compound **4** in DMSO-d_6. 49
Figure S44.^{13}C-HMBC NMR spectrum (600 MHz) of compound **4** in DMSO-d_6. 50
Figure S45. ^{1}H NMR spectrum (600 MHz) of compound (*Z*/*E*)-**5** in DMSO-d_6. 51
Figure S46. ^{13}C NMR spectrum (150 MHz) of compound (*E*/*Z*)-**5** in DMSO-d_6. 52
Figure S47. COSY NMR spectrum (600 MHz) of compound (*E*/*Z*)-**5** in DMSO-d_6. 53
Figure S48. ^{13}C-HSQC NMR spectrum (600 MHz) of compound (*E*/*Z*)-**5** in DMSO-d_6. 54
Figure S49. ^{13}C-HMBC NMR spectrum (600 MHz) of compound (*E*/*Z*)-**5** in DMSO-d_6. 55
Figure S50. NOESY NMR spectrum (600 MHz) of compound (*E*/*Z*)-**5** in DMSO-d_6. 56
Figure S51. ^{1}H NMR spectrum (600 MHz) of compound *Z*-**6** in DMSO-d_6. 57
Figure S52. ^{13}C NMR spectrum (150 MHz) of compound *Z*-**6** in DMSO-d_6. 58
Figure S53. COSY NMR spectrum (600 MHz) of compound *Z*-**6** in DMSO-d_6. 59
Figure S54. ^{13}C-HSQC NMR spectrum (600 MHz) of compound *Z*-**6** in DMSO-d_6. 60
Figure S55. ^{13}C-HMBC NMR spectrum (600 MHz) of compound *Z*-**6** in DMSO-d_6. 61
Figure S56. ROESY NMR spectrum (600 MHz) of compound *Z*-**6** in DMSO-d_6. 62
Figure S57. ^{1}H NMR spectrum (600 MHz) of compound **7** in DMSO-d_6. 63
Figure S58. COSY NMR spectrum (600 MHz) of compound **7** in DMSO-d_6. 64
Figure S59. ^{13}C-HMQC NMR spectrum (600 MHz) of compound 7 in DMSO-d_6. 65
Figure S60. ^{13}C-HMBC NMR spectrum (600 MHz) of compound **7** in DMSO-d_6. 66
Figure S61. NOESY NMR spectrum (600 MHz) of compound **7** in DMSO-d_6. 67
Figure S62. ^{1}H NMR spectrum (600 MHz) of compound **8** in DMSO-d_6. 68
Figure S63. ^{13}C NMR spectrum (150 MHz) of compound **8** in DMSO-d_6. 69
Figure S64. COSY NMR spectrum (600 MHz) of compound **8** in DMSO-d_6. 70
Figure S65. ^{13}C-HSQC NMR spectrum (600 MHz) of compound **8** in DMSO-d_6. 71
Figure S66. ^{13}C-HMBC NMR spectrum (600 MHz) of compound **8** in DMSO-d_6. 72

Figure S67. ^{13}C-HMBC NMR spectrum (600 MHz) of compound **8** in DMSO-d_6. 73
Figure S68. ^{13}C-HMBC NMR spectrum (600 MHz) of compound **8** in DMSO-d_6. 74
Figure S69. NOESY NMR spectrum (600 MHz) of compound **8** in DMSO-d_6. 75
Figure S70. HRMS^1 spectra of additional dimers identified during targeted re-examination of the *Tolypothrix* sp. PCC9009 biomass extract 76
Figure S71. Proposed structures of compounds **A** to **E** as postulated based on HRMS^2 data. 77
Figure S72. HRMS data of compound (*E*/*Z*)-**A**. 78
Figure S73. HRMS data of compound (*E*/*Z*)-**B**. 79
Figure S74. HRMS data of compound **C**. 81
Figure S75. HRMS data of compound (*E*/*Z*)-**D**. 82
Figure S76. HRMS data of compound (*E*/*Z*)-**E**. 83
Figure S77. HRMS^1 spectra of corresponding putatively 3-hydroxy derivatives 84

List of Tables

Table S1. Annotation of key ions observed in the HRMS^2 spectrum of compound **1**. 12
Table S2. Annotation of key ions observed in the HRMS^2 spectrum of compound (*E*/*Z*)-**2**. 14
Table S3. Annotation of key ions observed in the HRMS^2 spectrum of compound (*E*/*Z*)-**3**. 15
Table S4. Annotation of key ions observed in the HRMS^2 spectrum of compound **4**. 16
Table S5. Annotation of key ions observed in the HRMS^2 spectrum of compound (*E*/*Z*)-**5**. 17
Table S6. Annotation of key ions observed in the HRMS^2 spectrum of compound (*Z*)-**6**. 18
Table S7. Annotation of key ions observed in the HRMS^2 spectrum of compound **7**. 19
Table S8. Annotation of key ions observed in the HRMS^2 spectrum of compound (*E*/*Z*)-**A**....................78
Table S9. Annotation of key ions observed in the HRMS^2 spectrum of compound (*E*/*Z*)-**B**....................79
Table S10. Annotation of key ions observed in the HRMS^2 spectrum of compound **C** 81
Table S11. Annotation of key ions observed in the HRMS^2 spectrum of compound (*E*/*Z*)-**D**..................82
Table S12. Annotation of key ions observed in the HRMS^2 spectrum of compound (*E*/*Z*)-**E**..................83
Table S13. Sulforhodamine B Cytotoxicity Assay. 85

```
QUERY scaninfo(MS1DATA) WHERE #monoCl
MS1MZ=X:TOLERANCEMZ=0.1:INTENSITYPERCENT=25:INTENSITYMATCH=Y:INTENSITYMATCH
REFERENCE AND
MS1MZ=X+2:TOLERANCEMZ=0.1:INTENSITYMATCH=Y*0.32:INTENSITYMATCHPERCENT=30
AND
MS2PREC=X:TOLERANCEMZ=4
|||
QUERY scaninfo(MS1DATA) WHERE #diCl
MS1MZ=X:TOLERANCEMZ=0.1:INTENSITYPERCENT=25:INTENSITYMATCH=Y:INTENSITYMATCH
REFERENCE AND
MS1MZ=X+2:TOLERANCEMZ=0.1:INTENSITYMATCH=Y*0.64:INTENSITYMATCHPERCENT=20
AND
MS1MZ=X+4:TOLERANCEMZ=0.1:INTENSITYMATCH=Y*0.102:INTENSITYMATCHPERCENT=30
AND
MS2PREC=X:TOLERANCEMZ=4
|||
QUERY scaninfo(MS1DATA) WHERE #triCl
MS1MZ=X:TOLERANCEMZ=0.1:INTENSITYPERCENT=25:INTENSITYMATCH=Y:INTENSITYMATCH
REFERENCE AND
MS1MZ=X+2:TOLERANCEMZ=0.1:INTENSITYMATCH=Y*0.96:INTENSITYMATCHPERCENT=10 AND
MS1MZ=X+4:TOLERANCEMZ=0.1:INTENSITYMATCH=Y*0.31:INTENSITYMATCHPERCENT=30
AND
MS2PREC=X:TOLERANCEMZ=4)
|||
QUERY scaninfo(MS1DATA) WHERE #tetraCl
MS1MZ=X:TOLERANCEMZ=0.1:INTENSITYPERCENT=25:INTENSITYMATCH=Y:INTENSITYMATCH
REFERENCE AND
MS1MZ=X+2:TOLERANCEMZ=0.1:INTENSITYMATCH=Y*0.48:INTENSITYMATCHPERCENT=20 AND
MS1MZ=X-2:TOLERANCEMZ=0.1:INTENSITYMATCH=Y*0.78:INTENSITYMATCHPERCENT=20 AND
MS1MZ=X+4:TOLERANCEMZ=0.1:INTENSITYMATCH=Y*0.102:INTENSITYMATCHPERCENT=30
AND
MS2PREC=X:TOLERANCEMZ=4
```

Figure S1. First MassQL query to detect chlorinated specialized metabolites in the dataset. The query was designed to capture mono- to tetrachlorinated compounds.

$$\frac{P_{X+2}}{P_X} = \frac{w(w-1)c^2}{2(100-c)^2} \quad \textbf{(1)}$$

$$0.299 = \frac{70(70-1)1.1^2}{2(100-1.1)^2} \quad \textbf{(2)}$$

$$0.34 = \frac{75(75-1)c^2}{2(100-1.1)^2} \quad \textbf{(3)}$$

Figure S2. Calculation **(1)** to estimate the ^{13}C X+2 peak intensity (**P_X** probability of monoisotopic ions ^{12}C, **P_{X+2}** probability of an ion having two ^{13}C, **w** number of carbon atoms in the molecule, **c** natural abundance of ^{13}C as a percentage). For C_{70} **(2)**, the ratio P_{X+2}/P_X becomes 0.299, thus the X+2 peak will show as 29.9% intensity relative to the X peak (based on a reduced structure containing only C and H atoms as an approximation). For C_{75} **(3),** the ratio P_{X+2}/P_X becomes 0.34. Therefore, the natural isotope patterns will be similar to monochlorinated species, also considering the intensity tolerances that Haloseeker and MassQL queries are used with.

```
QUERY scaninfo(MS1DATA) WHERE #monoCl
MS1MZ=X:TOLERANCEMZ=0.1:INTENSITYPERCENT=25:INTENSITYMATCH=Y:INTENSITYMATCH
REFERENCE AND
MS1MZ=X+2:TOLERANCEMZ=0.1:INTENSITYMATCH=Y*0.32:INTENSITYMATCHPERCENT=10
AND
MS2PREC=X:TOLERANCEMZ=4 AND X=range(min=200, max=1000)
|||
QUERY scaninfo(MS1DATA) WHERE #diCl
MS1MZ=X:TOLERANCEMZ=0.1:INTENSITYPERCENT=25:INTENSITYMATCH=Y:INTENSITYMATCH
REFERENCE AND
MS1MZ=X+2:TOLERANCEMZ=0.1:INTENSITYMATCH=Y*0.64:INTENSITYMATCHPERCENT=20
AND
MS1MZ=X+4:TOLERANCEMZ=0.1:INTENSITYMATCH=Y*0.102:INTENSITYMATCHPERCENT=20
AND
MS2PREC=X:TOLERANCEMZ=4 AND X=range(min=200, max=1000)
|||
QUERY scaninfo(MS1DATA) WHERE #triCl
MS1MZ=X:TOLERANCEMZ=0.1:INTENSITYPERCENT=25:INTENSITYMATCH=Y:INTENSITYMATCH
REFERENCE AND
MS1MZ=X+2:TOLERANCEMZ=0.1:INTENSITYMATCH=Y*0.96:INTENSITYMATCHPERCENT=10 AND
MS1MZ=X+4:TOLERANCEMZ=0.1:INTENSITYMATCH=Y*0.31:INTENSITYMATCHPERCENT=20
AND
MS2PREC=X:TOLERANCEMZ=4 AND X=range(min=200, max=1000)
|||
QUERY scaninfo(MS1DATA) WHERE #tetraCl
MS1MZ=X:TOLERANCEMZ=0.1:INTENSITYPERCENT=25:INTENSITYMATCH=Y:INTENSITYMATCH
REFERENCE AND
MS1MZ=X+2:TOLERANCEMZ=0.1:INTENSITYMATCH=Y*0.48:INTENSITYMATCHPERCENT=20 AND
MS1MZ=X-2:TOLERANCEMZ=0.1:INTENSITYMATCH=Y*0.78:INTENSITYMATCHPERCENT=20 AND
MS1MZ=X+4:TOLERANCEMZ=0.1:INTENSITYMATCH=Y*0.102:INTENSITYMATCHPERCENT=30
AND
MS2PREC=X:TOLERANCEMZ=4 AND X=range(min=200, max=1000)
|||
QUERY scaninfo(MS1DATA) WHERE #pentaCl
MS1MZ=X:TOLERANCEMZ=0.1:INTENSITYPERCENT=25:INTENSITYMATCH=Y:INTENSITYMATCH
REFERENCE AND
MS1MZ=X+2:TOLERANCEMZ=0.1:INTENSITYMATCH=Y*0.64:INTENSITYMATCHPERCENT=20 AND
MS1MZ=X-2:TOLERANCEMZ=0.1:INTENSITYMATCH=Y*0.63:INTENSITYMATCHPERCENT=20 AND
MS1MZ=X+4:TOLERANCEMZ=0.1:INTENSITYMATCH=Y*0.21:INTENSITYMATCHPERCENT=30
AND
MS2PREC=X:TOLERANCEMZ=4 AND X=range(min=200, max=1000)
|||
QUERY scaninfo(MS1DATA) WHERE #hexaCl
MS1MZ=X:TOLERANCEMZ=0.1:INTENSITYPERCENT=25:INTENSITYMATCH=Y:INTENSITYMATCH
REFERENCE AND
MS1MZ=X+2:TOLERANCEMZ=0.1:INTENSITYMATCH=Y*0.8:INTENSITYMATCHPERCENT=20 AND
MS1MZ=X-2:TOLERANCEMZ=0.1:INTENSITYMATCH=Y*0.52:INTENSITYMATCHPERCENT=20 AND
MS1MZ=X+4:TOLERANCEMZ=0.1:INTENSITYMATCH=Y*0.34:INTENSITYMATCHPERCENT=20
AND
MS2PREC=X:TOLERANCEMZ=4 AND X=range(min=200, max=1000)
```

Figure S3. Optimized MassQL query to detect chlorinated specialized metabolites in the dataset. The query was designed to capture mono- to hexachlorinated species within the *m/z* range of 200-1000 Da, which was applied across the entire screening. Although ^{13}C isotope contributions specifically affect the detection of monochlorinated compounds, the range was set for all queries, including those for polychlorinated species, to ensure consistency and avoid potential bias in data selection throughout the screening process. The acceptance threshold for variability in relative intensity was also adjusted.

A

```
QUERY scaninfo(MS2DATA) WHERE
MS2PROD=78.9183:TOLERANCEPPM=10:INTENSITYPERCENT=1
and MS2PROD=80.9163:TOLERANCEPPM=10:INTENSITYPERCENT=1
```

B

```
QUERY scaninfo(MS2DATA) WHERE #monoBr
MS1MZ=X:INTENSITYMATCH=Y:INTENSITYMATCHREFERENCE:INTENSITYPERCENT=20
AND MS1MZ=X+2:INTENSITYMATCH=Y*0.97:INTENSITYMATCHPERCENT=10
AND MS2PREC=X:TOLERANCEMZ=4
|||
QUERY scaninfo(MS2DATA) WHERE #diBr
MS1MZ=X:TOLERANCEMZ=0.1:INTENSITYPERCENT=25:INTENSITYMATCH=Y:INTENSITYMATCH REFERENCE
AND MS1MZ=X+2:TOLERANCEMZ=0.1:INTENSITYMATCH=Y*0.48:INTENSITYMATCHPERCENT=30 AND
MS1MZ=X-2:TOLERANCEMZ=0.1:INTENSITYMATCH=Y*0.51:INTENSITYMATCHPERCENT=30 AND
MS2PREC=X:TOLERANCEMZ=4
|||
QUERY scaninfo(MS2DATA) WHERE #triBr
MS1MZ=X:TOLERANCEMZ=0.1:INTENSITYPERCENT=5:INTENSITYMATCH=Y:INTENSITYMATCHR EFERENCE
AND MS1MZ=X+2:TOLERANCEMZ=0.1:INTENSITYMATCH=Y*0.97:INTENSITYMATCHPERCENT=10
AND MS1MZ=X2:TOLERANCEMZ=0.1:INTENSITYMATCH=Y*0.34:INTENSITYMATCHPERCENT=30
AND MS1MZ=X+4:TOLERANCEMZ=0.2:INTENSITYMATCH=Y*0.32:INTENSITYMATCHPERCENT=40 AND
MS2PREC=X:TOLERANCEMZ=2
|||
QUERY scaninfo(MS2DATA) WHERE #tetraBr
MS1MZ=X:TOLERANCEMZ=0.1:INTENSITYPERCENT=25:INTENSITYMATCH=Y:INTENSITYMATCH REFERENCE
AND MS1MZ=X+2:TOLERANCEMZ=0.1:INTENSITYMATCH=Y*0.66:INTENSITYMATCHPERCENT=30
AND MS1MZ=X2:TOLERANCEMZ=0.1:INTENSITYMATCH=Y*0.66:INTENSITYMATCHPERCENT=30
AND MS1MZ=X+4:TOLERANCEMZ=0.2:INTENSITYMATCH=Y*0.17:INTENSITYMATCHPERCENT=40
AND MS1MZ=X4:TOLERANCEMZ=0.2:INTENSITYMATCH=Y*0.17:INTENSITYMATCHPERCENT=40
AND MS2PREC=X:TOLERANCEMZ=4
|||
QUERY scaninfo(MS2DATA) WHERE #pentaBr
MS1MZ=X:TOLERANCEMZ=0.1:INTENSITYPERCENT=25:INTENSITYMATCH=Y:INTENSITYMATCH REFERENCE
AND MS1MZ=X+2:TOLERANCEMZ=0.1:INTENSITYMATCH=Y*0.97:INTENSITYMATCHPERCENT=10
AND MS1MZ=X-2:TOLERANCEMZ=0.1:INTENSITYMATCH=Y*0.51:INTENSITYMATCHPERCENT=20
AND MS1MZ=X+4:TOLERANCEMZ=0.2:INTENSITYMATCH=Y*0.47:INTENSITYMATCHPERCENT=20
AND MS1MZ=X-4:TOLERANCEMZ=0.2:INTENSITYMATCH=Y*0.11:INTENSITYMATCHPERCENT=40
AND MS1MZ=X+6:TOLERANCEMZ=0.2:INTENSITYMATCH=Y*0.09:INTENSITYMATCHPERCENT=40
AND MS2PREC=X:TOLERANCEMZ=2
```

Figure S4. MassQL queries to detect brominated specialized metabolites in the dataset. **A** MassQL query to mine the datasets for bromine compounds, if bromide (*m/z* 78.918 and 80.916) fragment ions are present in the high-resolution tandem mass spectra. **B** MassQL query to detect mono- to pentabrominated specialized metabolites in the dataset due to characteristic MS^1 isotope patterns.

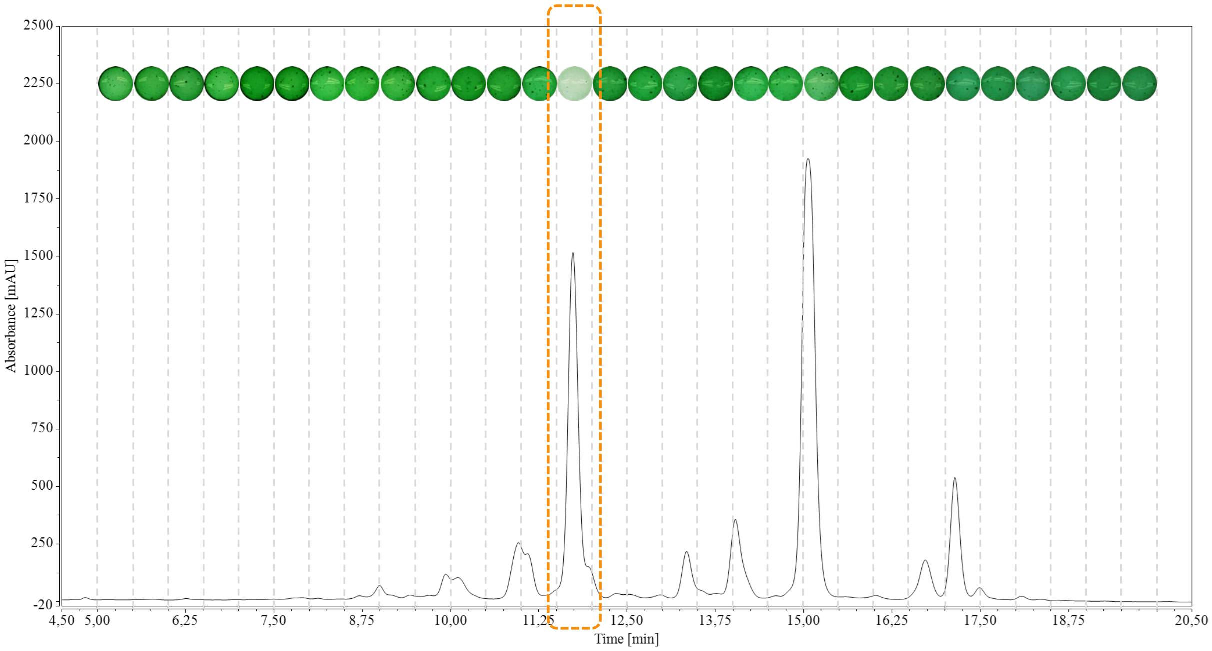

Figure S5. Microfractionation and results from the assays for herbicidal activity of the *Tolypothrix* sp. PCC9009 biomass extract. 30 microfractions were collected. Each microfraction was added to diluted, growing *Synechocystis* sp. PCC6803 liquid cultures. Culture images illustrate state of growth after 14 days after fraction addition. The only active microfraction, containing CB, is highlighted. The liquid culture treated with the CB containing fraction exhibited poor growth as indicated by the noticeably paler coloration.

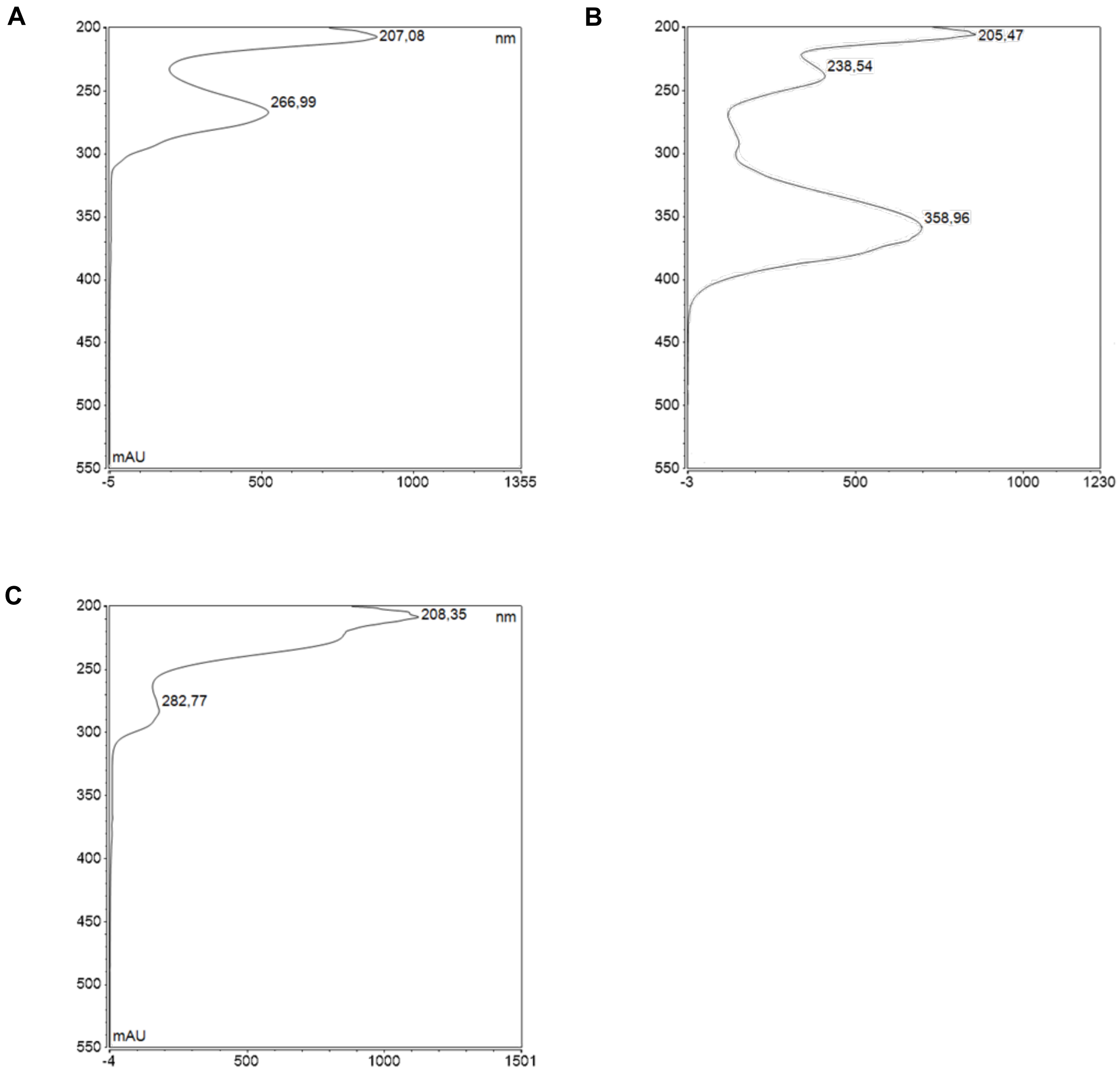

Figure S6. UV spectra of **A** cyanobacterin (**4**), **B** anhydrocyanobacterin (**1**) as representative of all analogues of **1**, and **C** Bishanydrocyanobacterin as representative of dimers, **2** exhibiting a similar UV spectrum. Spectra shown have been recorded by HPLC-DAD (Dionex UltiMate 3000, Thermo Scientific, undefined H_2O/MeCN mixture + 0.1% formic acid).

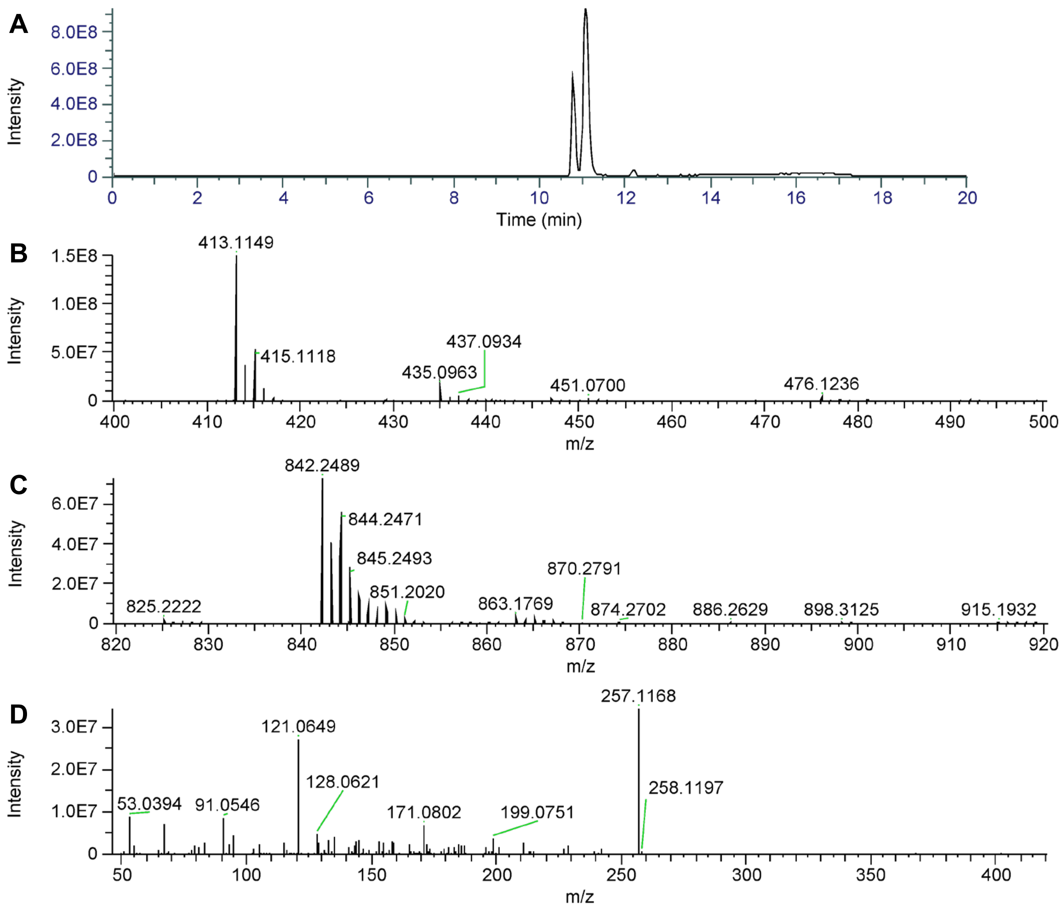

Figure S7. HRMS data of (*E*/*Z*)-**1** (ESI pos. mode). **A** BPC of *Z*-**1** two weeks after isolation, showing establishment of *E*/*Z* equilibrium and dimerization. **B** $HRMS^1$ spectrum (*m/z* 413.1149) showing the characteristic isotope pattern. **C** $HRMS^1$ spectrum of **8** (*m/z* 842.2222), formed from initially pure *Z*-isomer. **D** $HRMS^2$ spectrum (precursor ion *m/z* 413.1149), CE 55 eV.

Table S1. Annotation of key ions observed in the $HRMS^2$ spectrum of (*E*/*Z*)-**1**.

m/z	mol. formula	proposed structure
257.1168	$C_{16}H_{17}O_3^+$ (Δ 0.8 ppm)	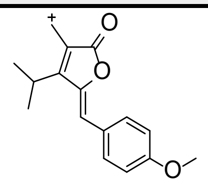
121.0649	$C_8H_9O^+$ (Δ 0.8 ppm)	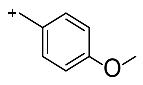

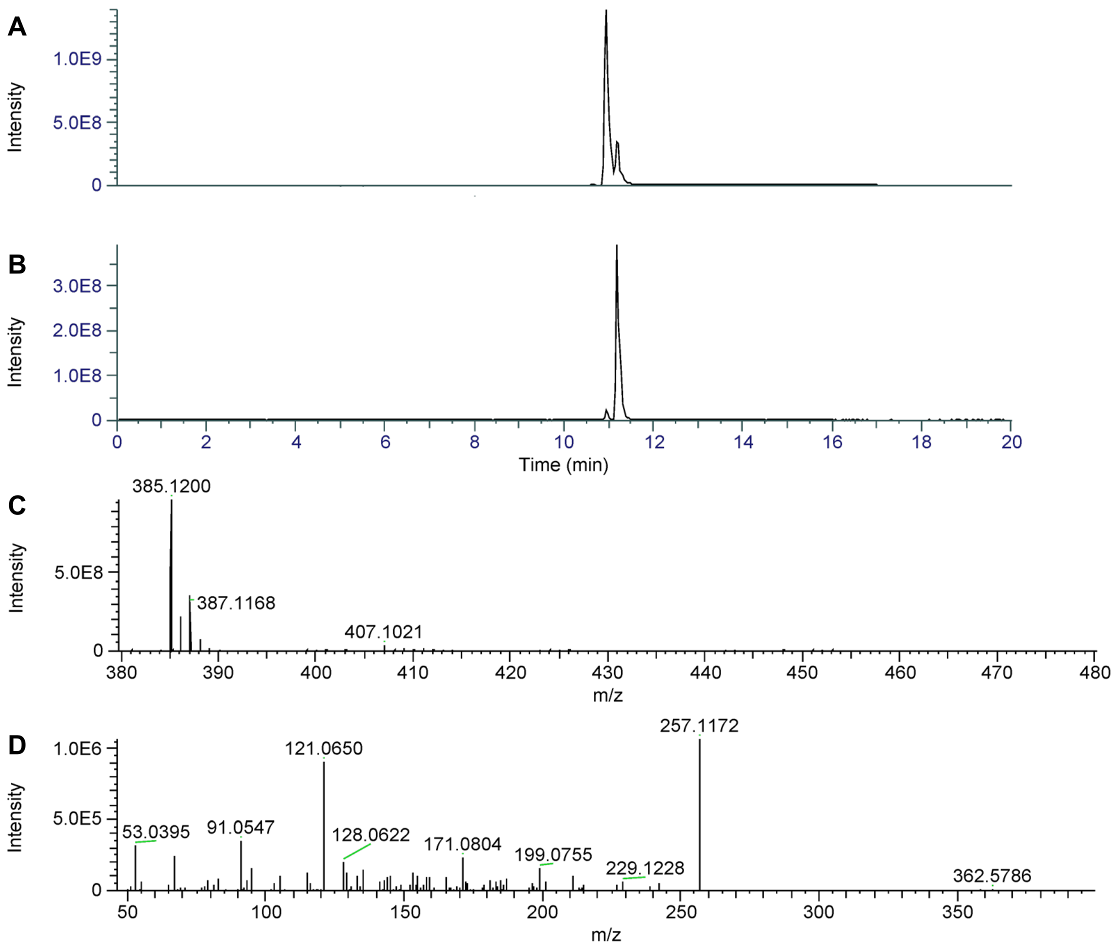

Figure S8. HRMS data of **2**. **A** Extracted Ion Chromatogram of *E*-**2**, both isomers were present due to swift isomerization. **B** Extracted Ion Chromatogram of *Z*-**2**, both isomers were present due to swift isomerization. **C** HRMS[1] spectrum (ESI pos. mode, *m/z* 385.1200) showing the characteristic isotope pattern. **D** HRMS[2] spectrum (precursor ion *m/z* 385.1200), ESI pos. mode, collision energy 55 eV.

Table S2. Annotation of key ions observed in the HRMS^2 spectrum of (*E*/*Z*)-**2**.

m/z	mol. formula	proposed structure
257.1172	$C_{16}H_{17}O_3^+$ (Δ 0.8 ppm)	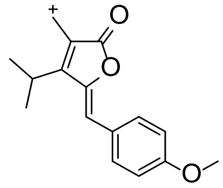
141.0104	$C_7H_6O^+$ (Δ 1.4 ppm)	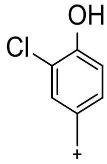
121.0649	$C_8H_9O^+$ (Δ 0.8 ppm)	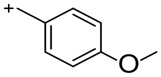

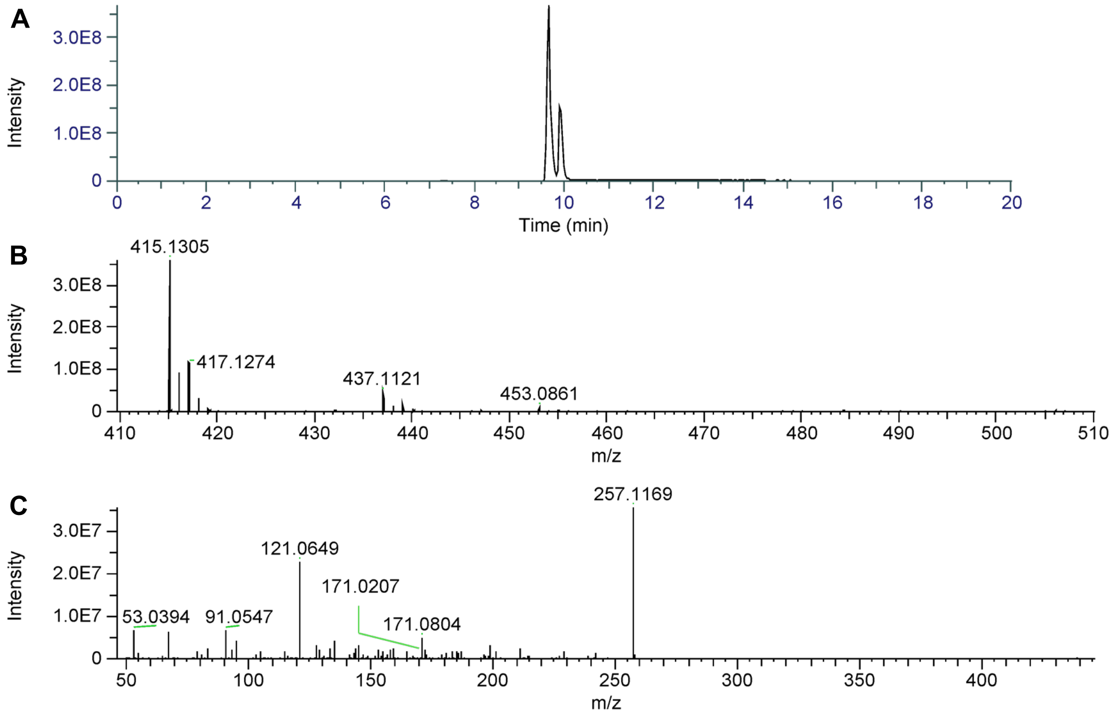

Figure S9. HRMS data of (*E*/*Z*)-**3** (ESI pos. mode). **A** EIC of (*E*/*Z*)-**3**, both isomers were present due to non-separation under the chromatographic conditions used during isolation. **B** HRMS[1] spectrum (*m/z* 415.1305) showing the characteristic isotope pattern. **C** HRMS[2] spectrum (precursor ion *m/z* 415.1305), CE 55 eV.

Table S3. Annotation of key ions observed in the HRMS[2] spectrum of (*E*/*Z*)-**3**.

m/z	mol. formula	proposed structure
257.1169	$C_{16}H_{17}O_3^+$ (Δ 0.4 ppm)	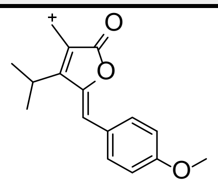
171.0207	$C_8H_8ClO_2^+$ (Δ 0.0 ppm)	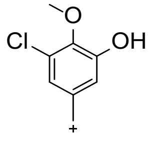
121.0649	$C_8H_9O^+$ (Δ 0.8 ppm)	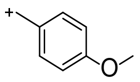

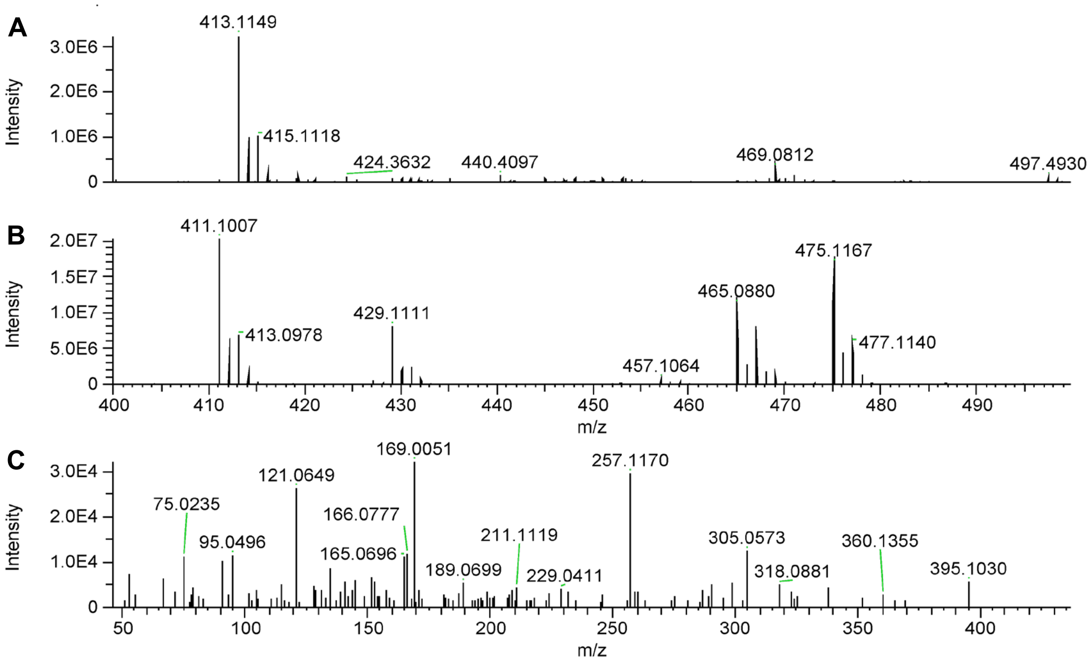

Figure S10. HRMS data of **4**. **A** HRMS[1] spectrum (ESI pos. mode, *m/z* 413.1149), characteristic ion peak with neutral loss of water and chlorine isotope pattern. **B** HRMS[1] spectrum (neg. mode) shows parent ion peak (*m/z* 429.1111) and peak with water loss (*m/z* 411.1007), each with the chlorine isotope pattern. **C** HRMS[2] spectrum (precursor ion *m/z* 413.1149), pos. mode, CE 55 eV.

Table S4. Annotation of key ions observed in the HRMS[2] spectrum of **4**.

m/z	mol. formula	proposed structure
305.0573	$C_{16}H_{14}ClO_4^+$ (Δ 0.7 ppm)	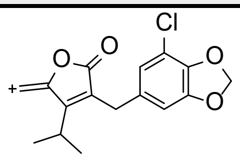
257.1172	$C_{16}H_{17}O_3^+$ (Δ 0.8 ppm)	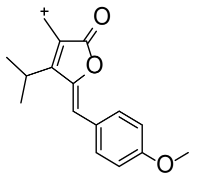
169.0051	$C_8H_6ClO_2^+$ (Δ 0.0 ppm)	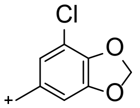
121.0649	$C_8H_9O^+$ (Δ 0.8 ppm)	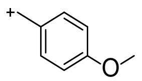

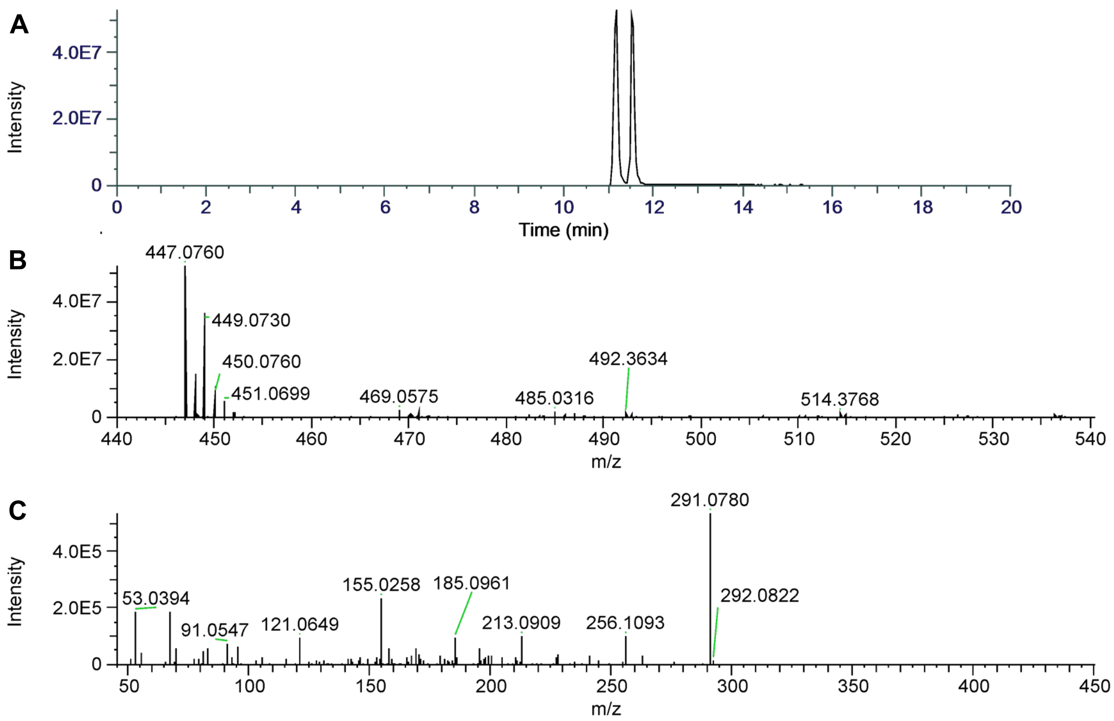

Figure S11. HRMS data of (*E*/*Z*)-**5**. **A** Extracted Ion Chromatogram of (*E*/*Z*)-**5**, both isomers were present due to non-separation under the chromatographic conditions used during isolation. **B** $HRMS^1$ spectrum (ESI pos. mode, *m/z* 447.0760) showing the characteristic isotope pattern. **C** $HRMS^2$ spectrum (precursor ion *m/z* 447.0760), ESI pos. mode, collision energy 55 eV.

Table S5. Annotation of key ions observed in the $HRMS^2$ spectrum of (*E*/*Z*)-**5**.

m/z	mol. formula	proposed structure
291.0780	$C_{16}H_{16}ClO_3^+$ (Δ 0.7 ppm)	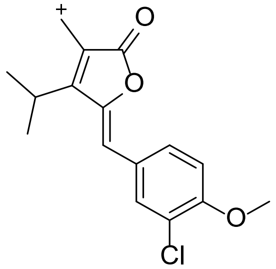
155.0258	$C_8H_8ClO^+$ (Δ 0 ppm)	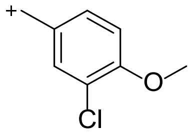

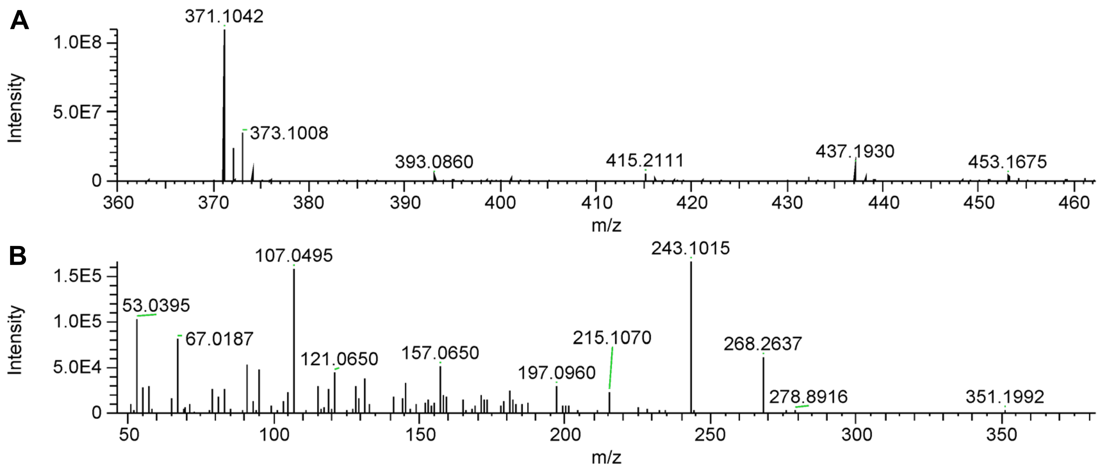

Figure S12. HRMS data of (*Z*)-**6**. **A** $HRMS^1$ spectrum (ESI pos. mode, *m/z* 371.1042) showing the characteristic isotope pattern. **B** $HRMS^2$ spectrum (precursor ion *m/z* 371.1042), ESI pos. mode, collision energy 55 eV.

Table S6. Annotation of key ions observed in the $HRMS^2$ spectrum of (*Z*)-**6**.

m/z	mol. formula	proposed structure
243.1015	$C_{16}H_{16}ClO_3^+$ (Δ 0.4 ppm)	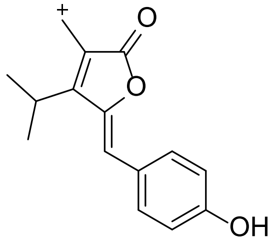
187.1112	$C_8H_8ClO^+$ (Δ 2.7 ppm)	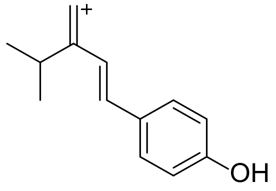
107.0495	$C_7H_7O^+$ (Δ 3.7 ppm)	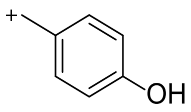

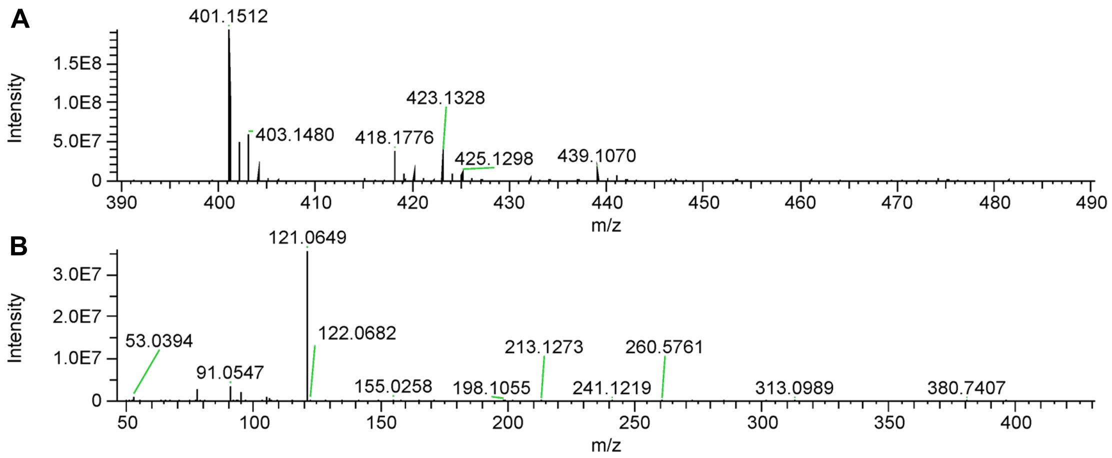

Figure S13. HRMS data of **7**. **A** HRMS[1] spectrum (ESI pos. mode, *m/z* 401.1512) showing the characteristic isotope pattern. **B** HRMS[2] spectrum (precursor ion *m/z* 401.1512), ESI pos. mode, collision energy 55 eV.

Table S7. Annotation of key ions observed in the HRMS[2] spectrum of **7**.

m/z	mol. formula	proposed structure
155.0258	$C_8H_8ClO_3^+$ (Δ 0.0 ppm)	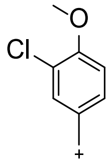
121.0649	$C_8H_9O^+$ (Δ 0.8 ppm)	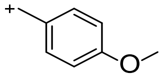

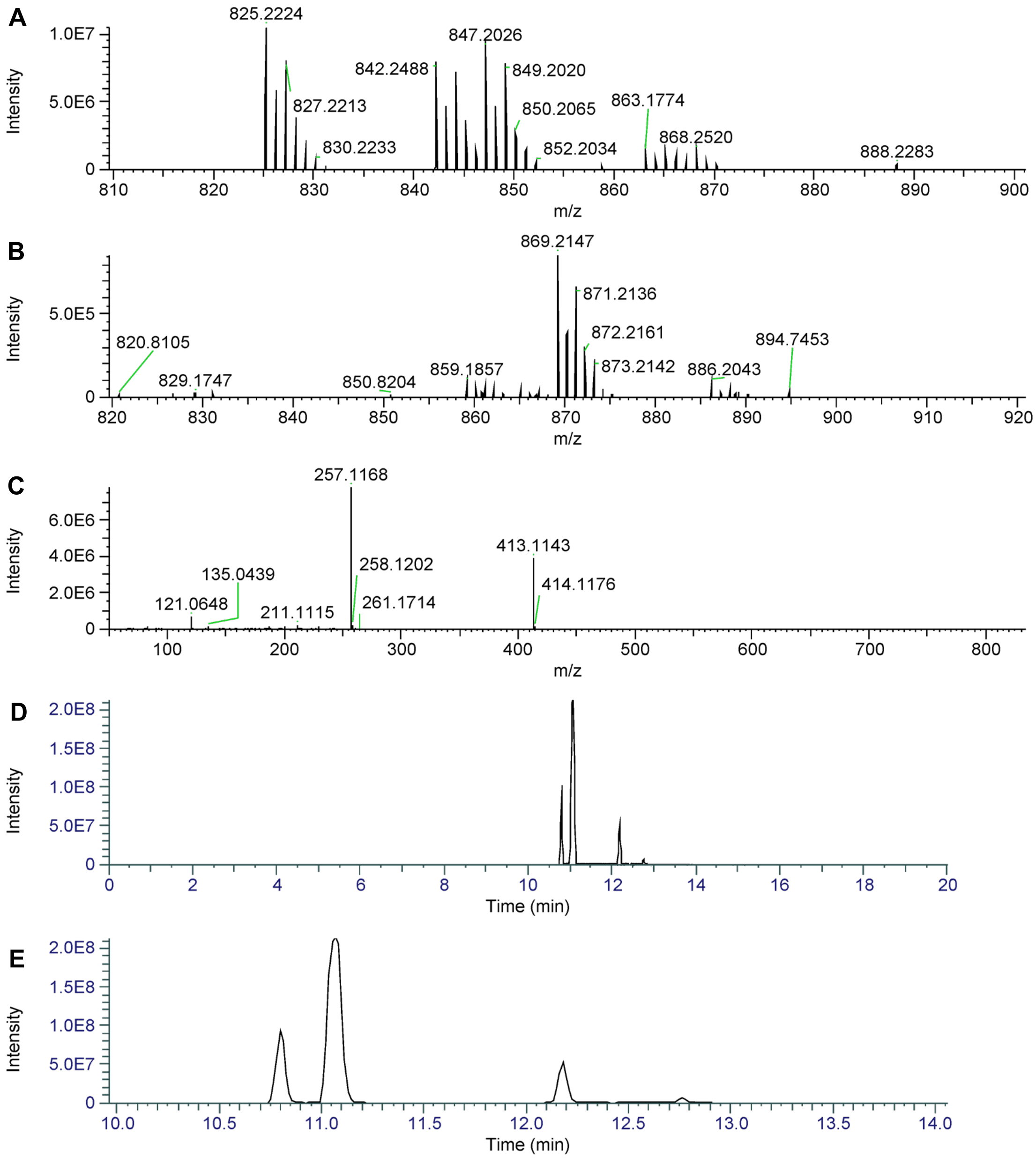

Figure S14. HRMS data of **8**. **A** HRMS[1] spectrum (ESI pos. mode, $[M+H]^+$ *m/z* 825.2224) showing the characteristic isotope pattern. **B** HRMS[1] spectrum (ESI neg. mode, $[M+CHO_2]^-$ *m/z* 869.2147). **C** HRMS[2] spectrum (precursor ion *m/z* 825.2224), ESI pos. mode, CE 55 eV, showing fragment ion of corresponding monomer and characteristic fragment ions shown previously. **D** EIC of **8** from biomass extracts at *m/z* 825.2224, showing four distinct signals: peaks at t_R~ 11 min correspond to **1** dimers formed during the ionization process, peak at t_R=12.18 min corresponds to **8,** and t_R=12.78 min may correspond to either a constitutional isomer or a configurational isomer of **8**. **E** Zoomed-in view of the EIC at t_R =10-14 min.

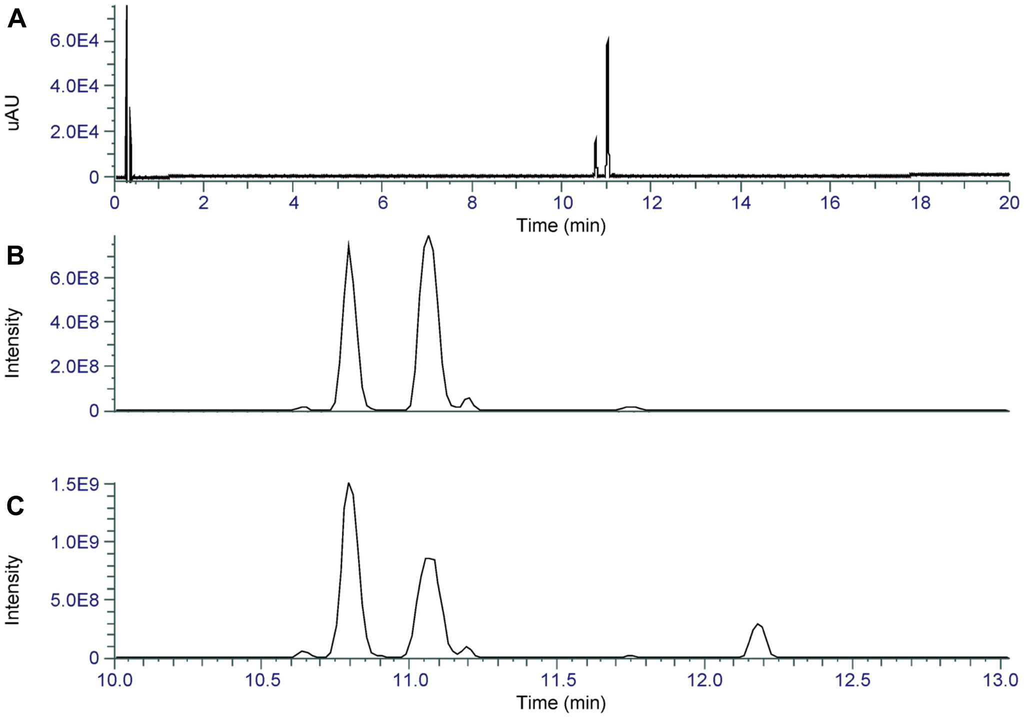

Figure S15. Dimer formation (*E*/*Z*)-**1**. **A** UV/Vis chromatogram of **1** prior to UV exposure **B** Base peak chromatogram of **1** prior to UV exposure, zoomed-in view at t_R=10-13 min. **C** Base peak chromatogram of **1** after UV exposure (t=180 min), revealing the formation of **8**.

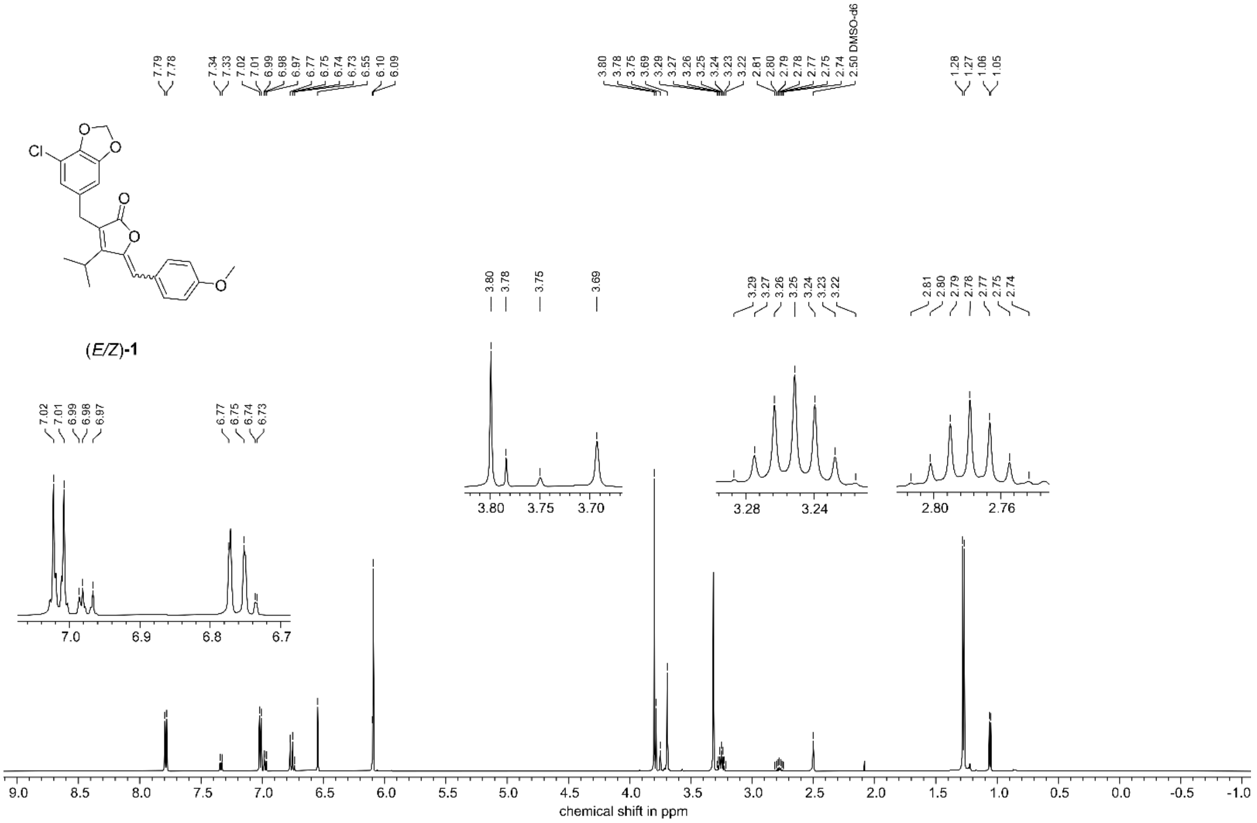

Figure S16. ^{1}H NMR spectrum (600 MHz) of (*E*/*Z*)-**1** in DMSO-d_6.

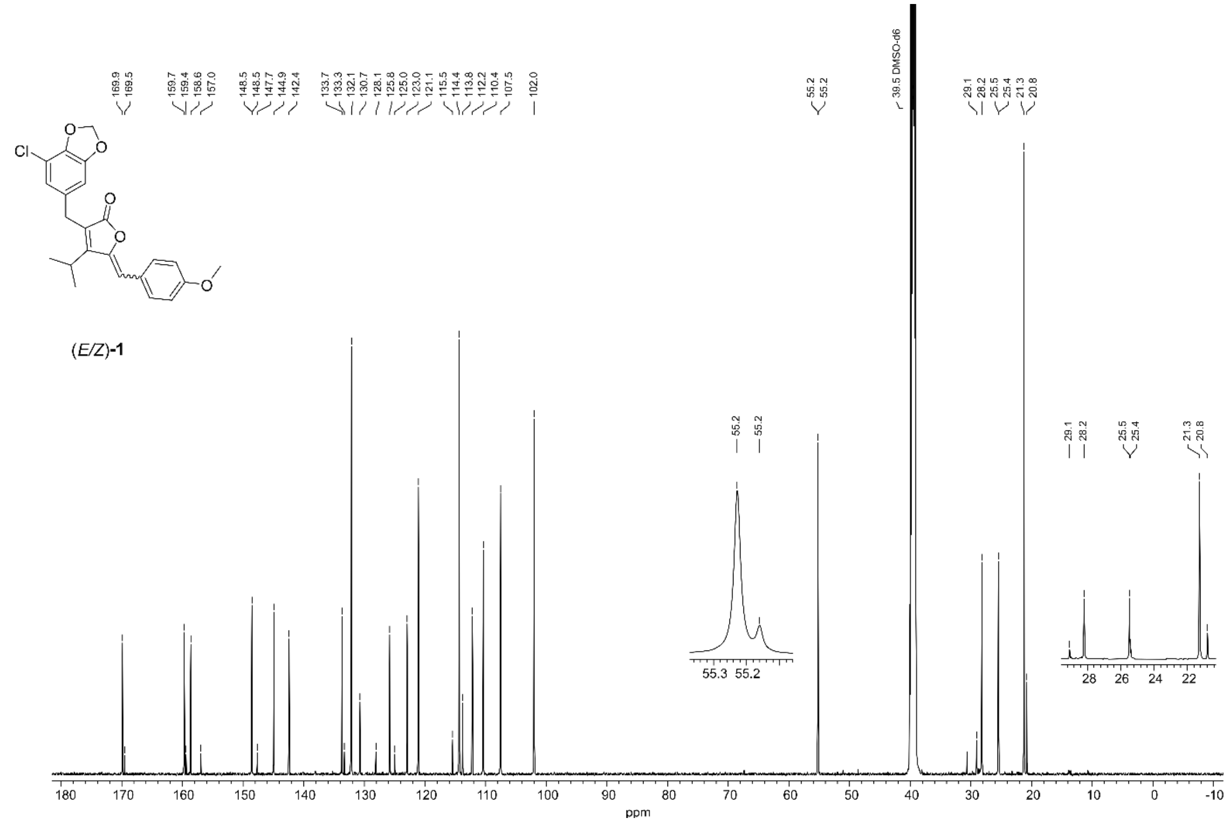

Figure S17. ^{13}C NMR spectrum (150 MHz) of (*E*/*Z*)-**1** in DMSO-d_6.

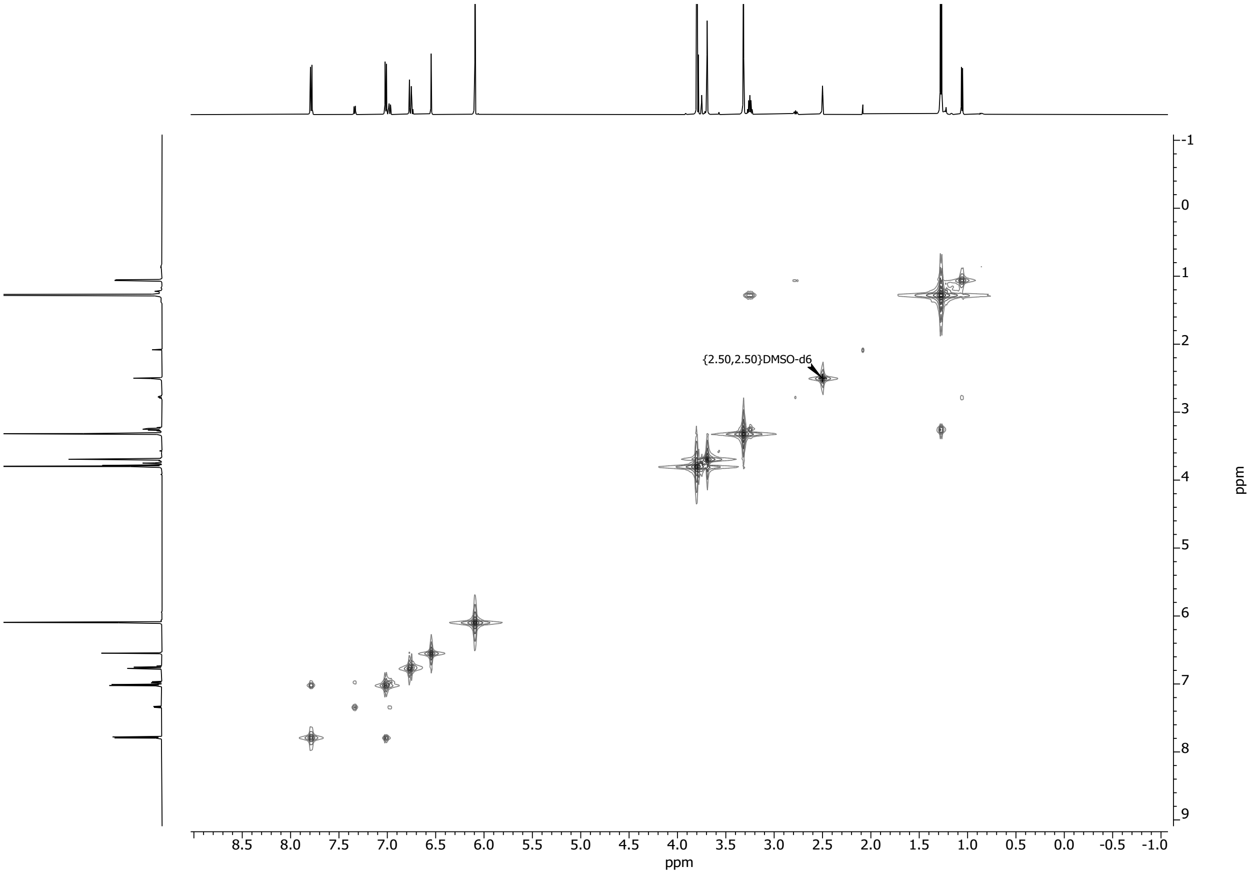

Figure S18. COSY NMR spectrum (600 MHz) of (*E*/*Z*)-**1** in DMSO-d_6.

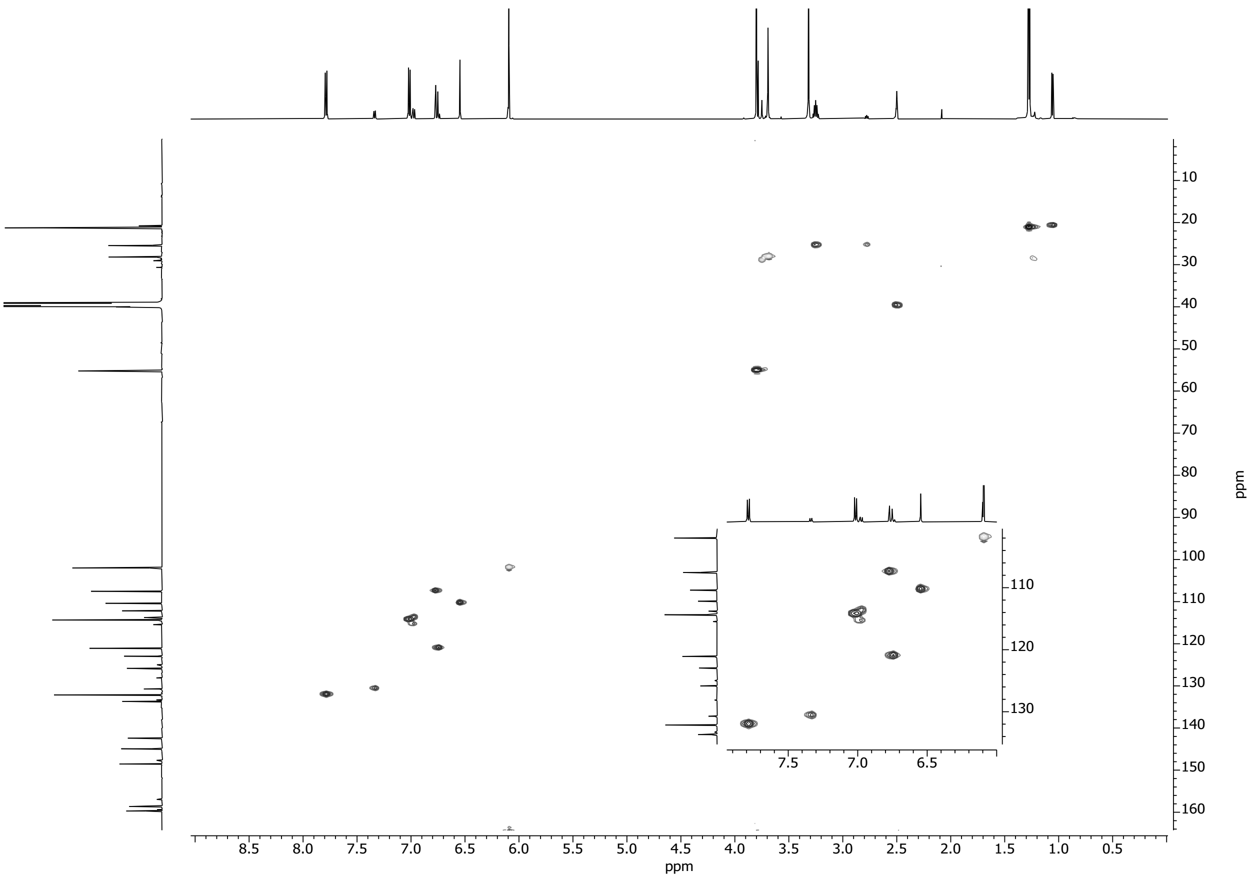

Figure S19. ^{13}C-HMQC NMR spectrum (600 MHz) of (*E*/*Z*)-**1** in DMSO-*d*$_6$.

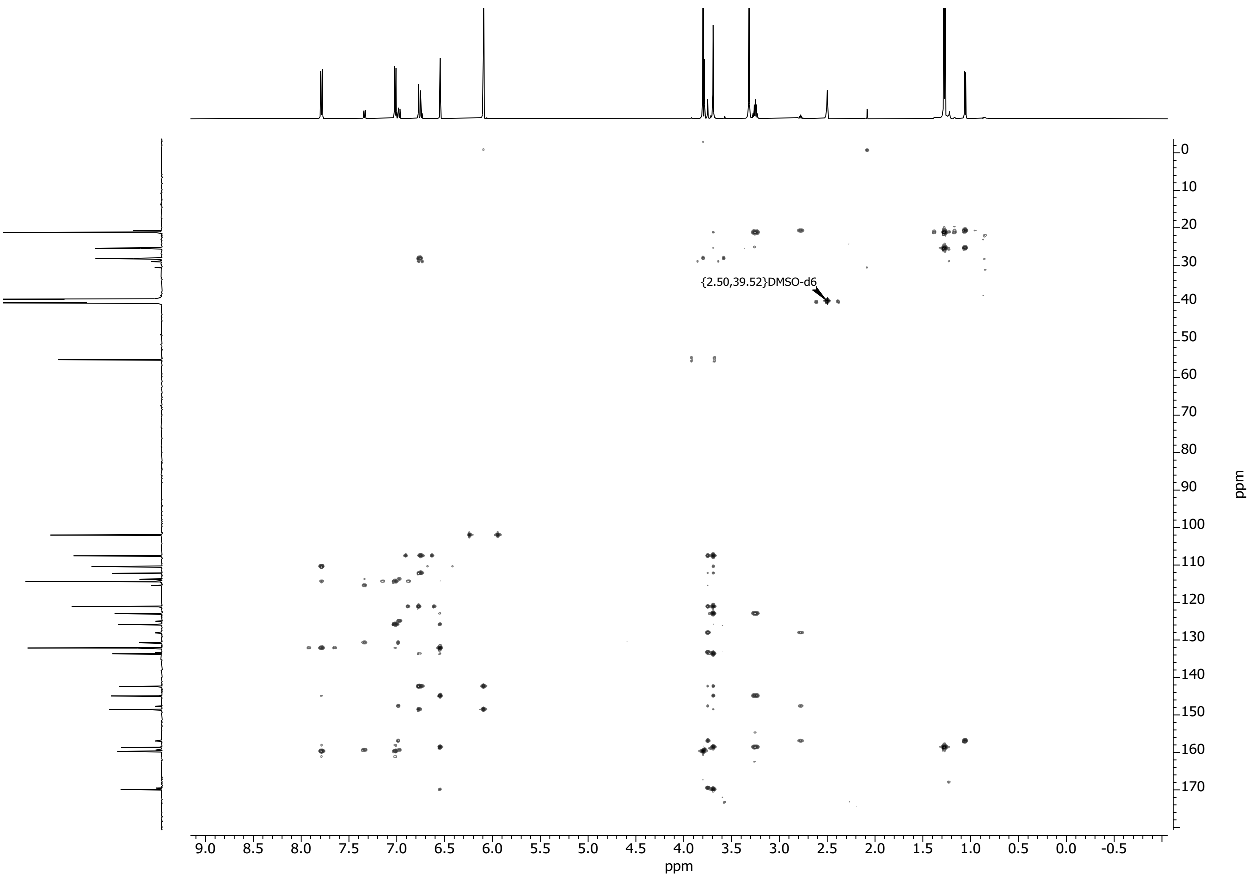

Figure S20. ^{13}C-HMBC NMR spectrum (600 MHz) of (*E*/*Z*)-**1** in DMSO-d_6.

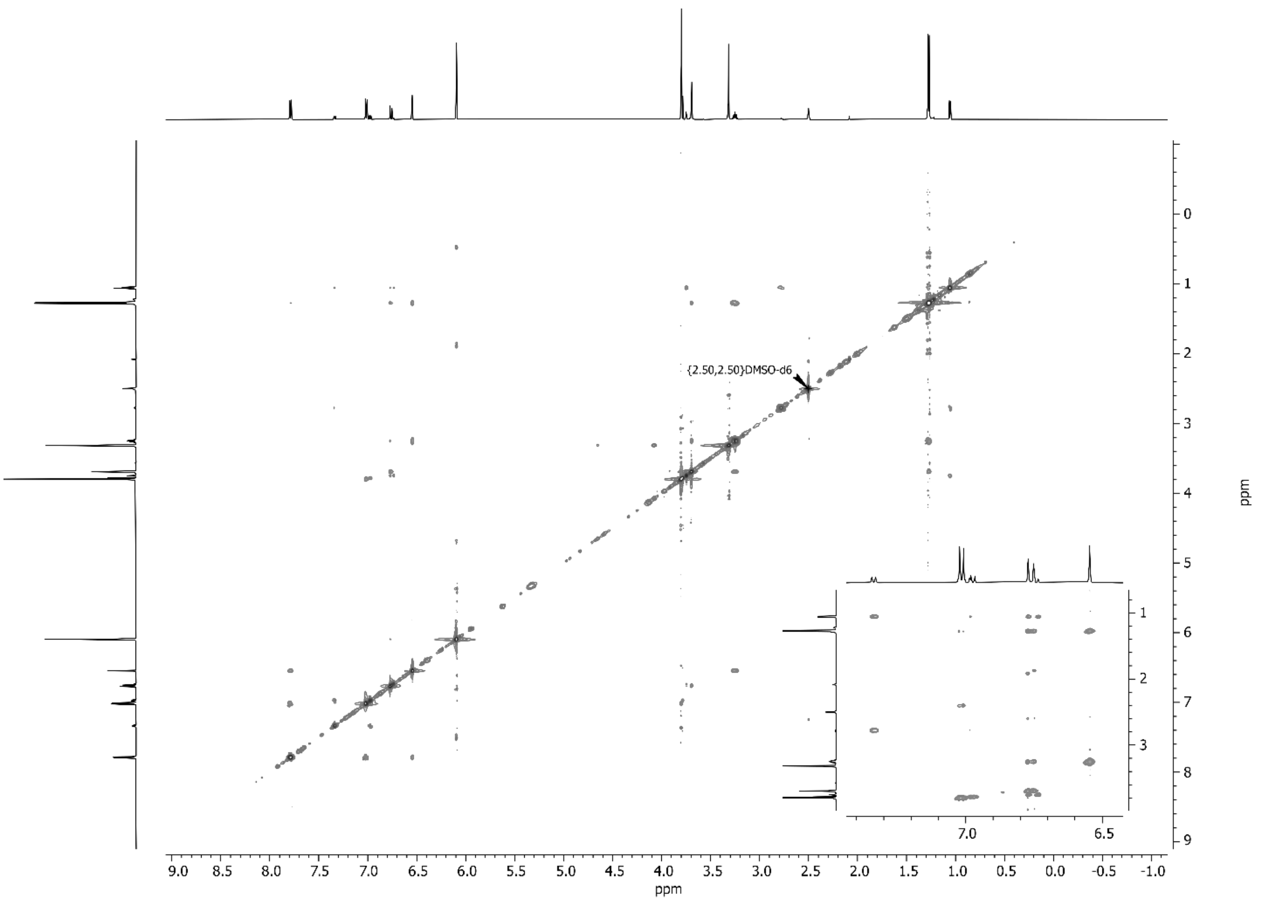

Figure S21. NOESY NMR spectrum (600 MHz) of (E/Z)-**1** in DMSO-d_6. Insets show expanded regions of the spectrum from δ_H 6.0-8.0 ppm and δ_H 1.0-4.0 ppm, highlighting representative NOE correlations used to distinguish between the E and Z isomers.

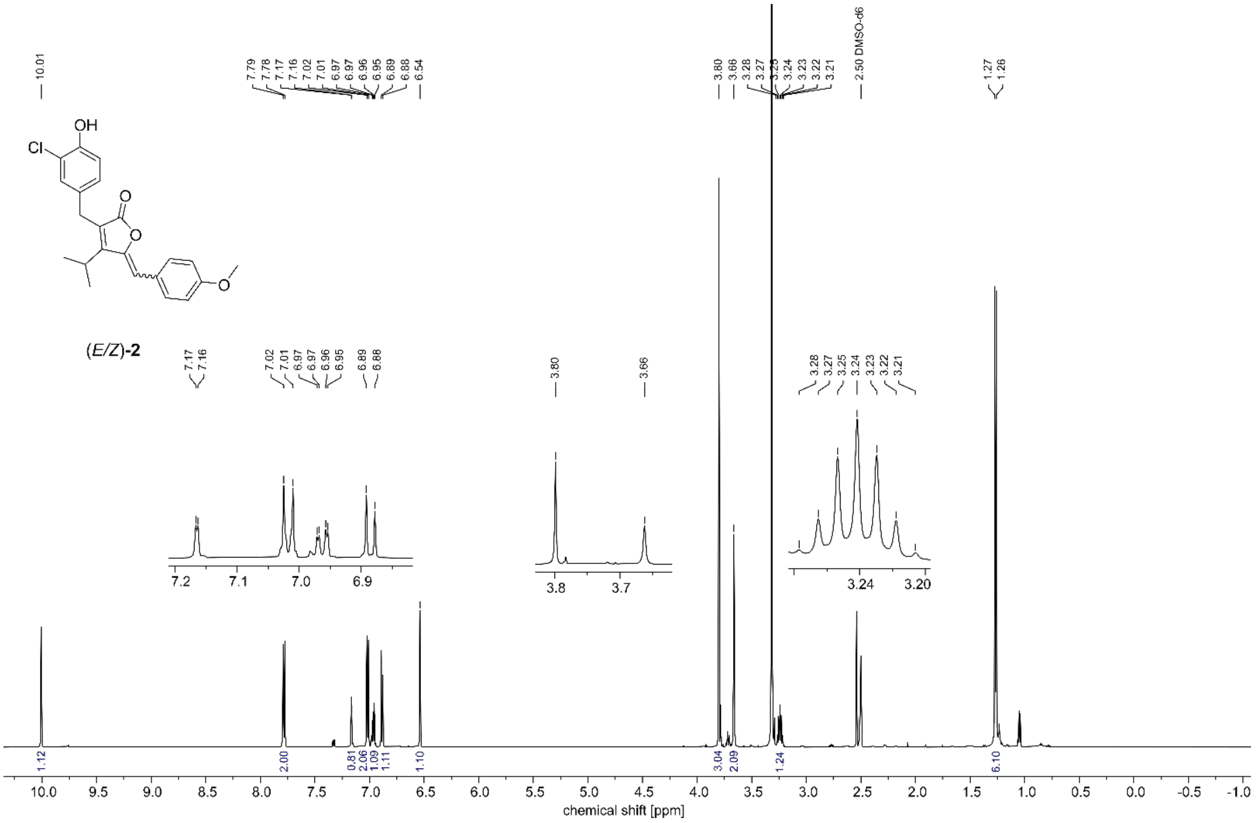

Figure S22. ^{1}H NMR spectrum (600 MHz) of (*Z*)-**2** in DMSO-d_6. Due to swift isomerization, signals of (*E*)-**2** are visible too. Integral values are reported only for (*Z*)-**2**, as it is the only candidate where unambiguous signal assignment and reliable integration are possible. In the ^{1}H NMR spectra of the other compounds (except **4** and (*Z*)-**6**), overlapping signals from both isomers prevent accurate integration.

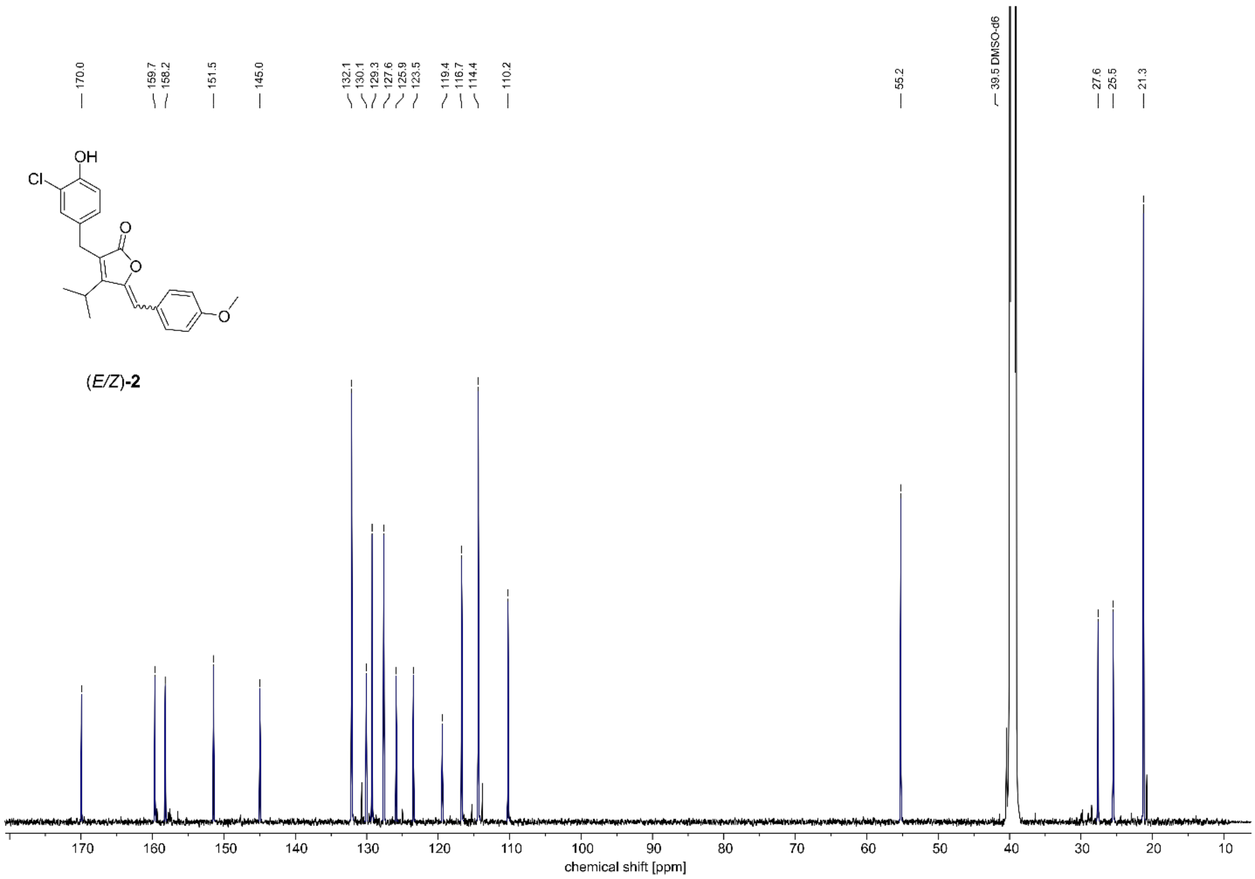

Figure S23. ^{13}C NMR spectrum (150 MHz) of (*Z*)-**2** in DMSO-d_6.

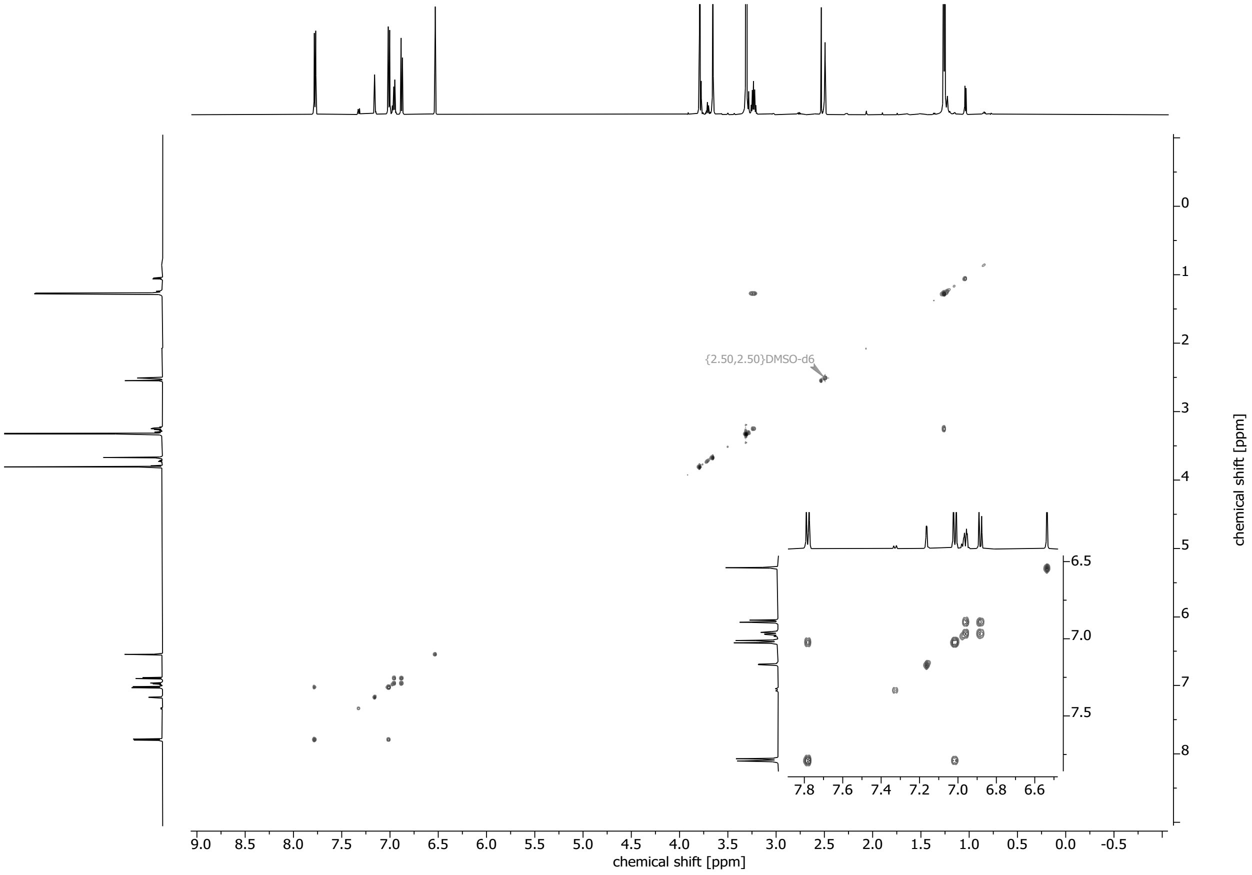

Figure S24. COSY NMR spectrum (600 MHz) of (*Z*)-**2** in DMSO-d_6.

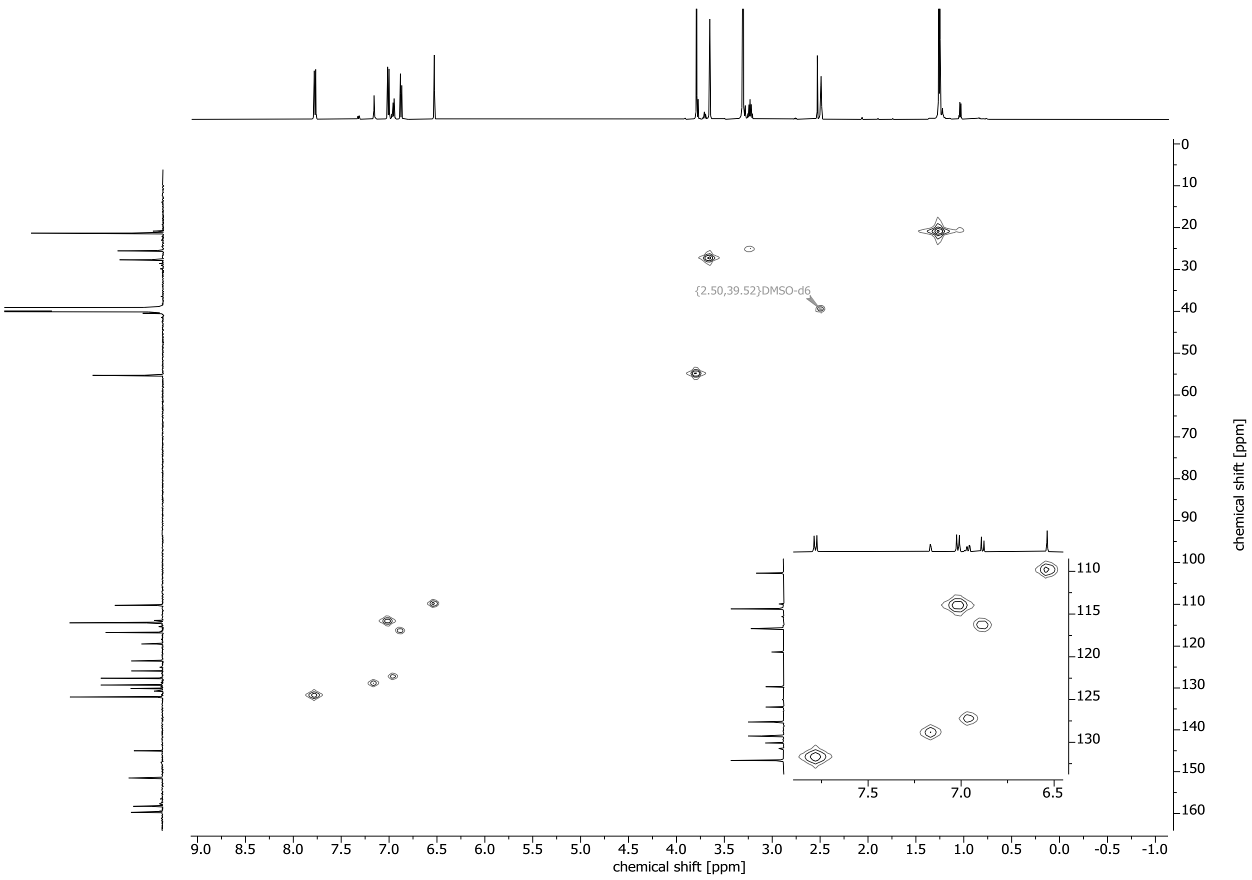

Figure S25. ^{13}C-HMQC NMR spectrum (600 MHz) of (*Z*)-**2** in DMSO-d_6.

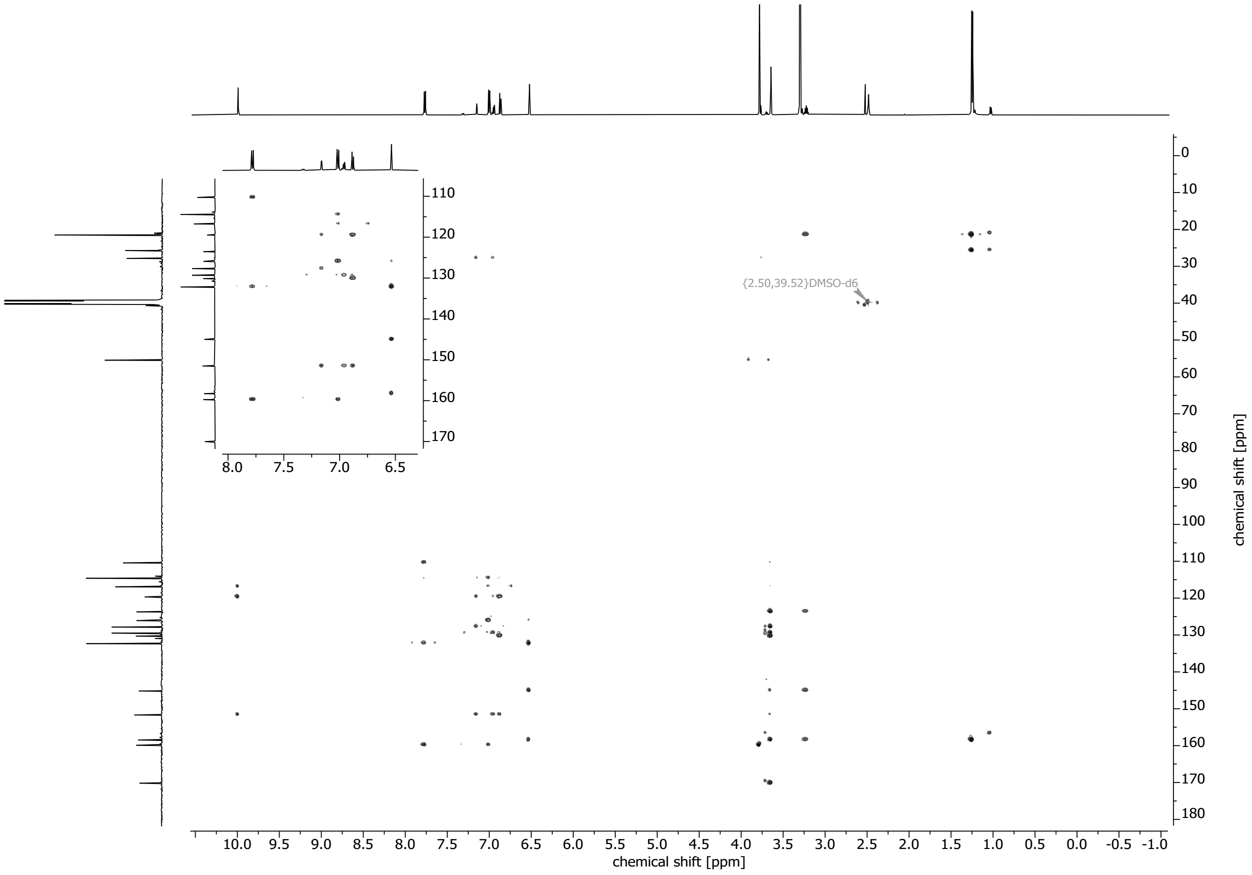

Figure S26. ^{13}C-HMBC NMR spectrum (600 MHz) of (*Z*)-**2** in DMSO-d_6.

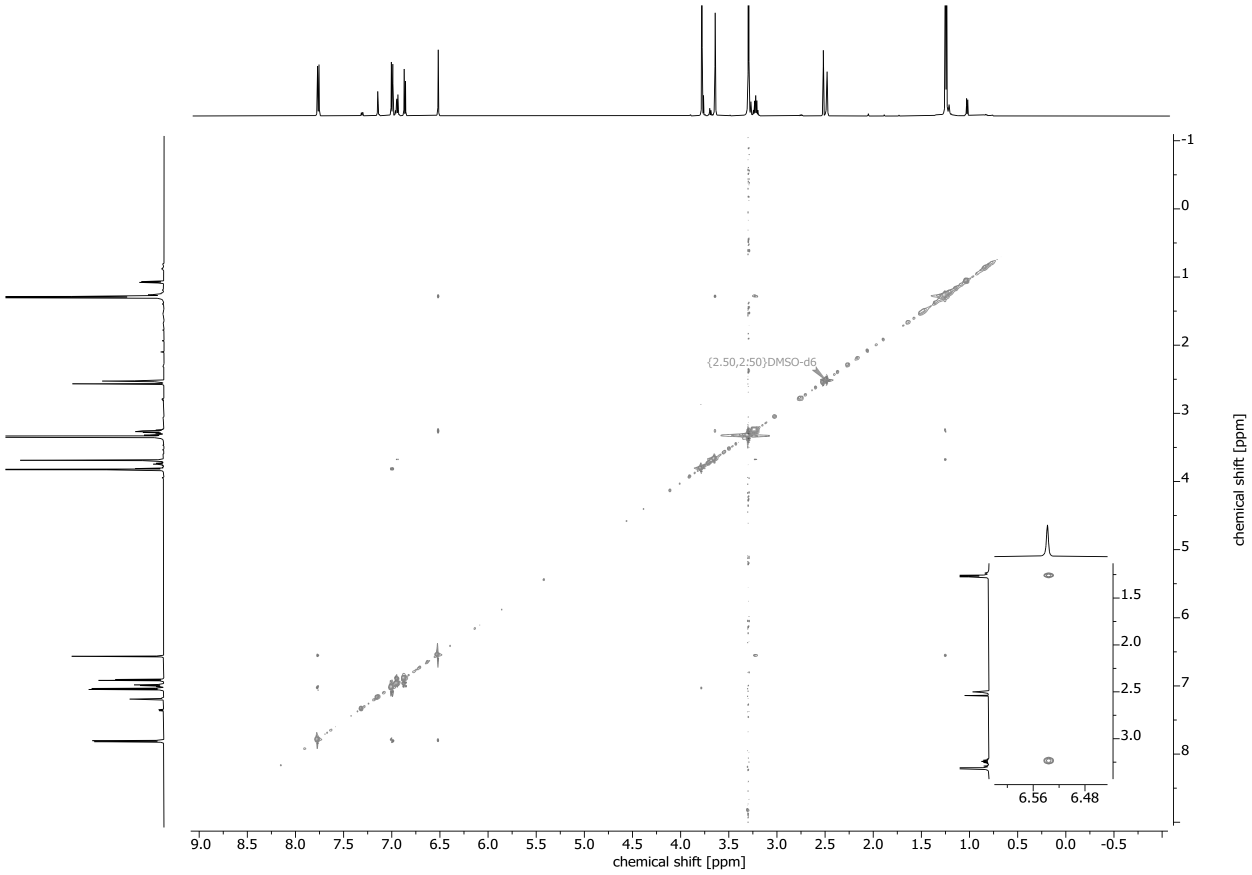

Figure S27. NOESY NMR spectrum (600 MHz) of (*Z*)-**2** in DMSO-d_6. Inset shows expanded region of the spectrum from δ_H 6.43-6.56 ppm and δ_H 1.0-3.5 ppm, highlighting representative NOE correlations used to distinguish between the *E* and *Z* isomers.

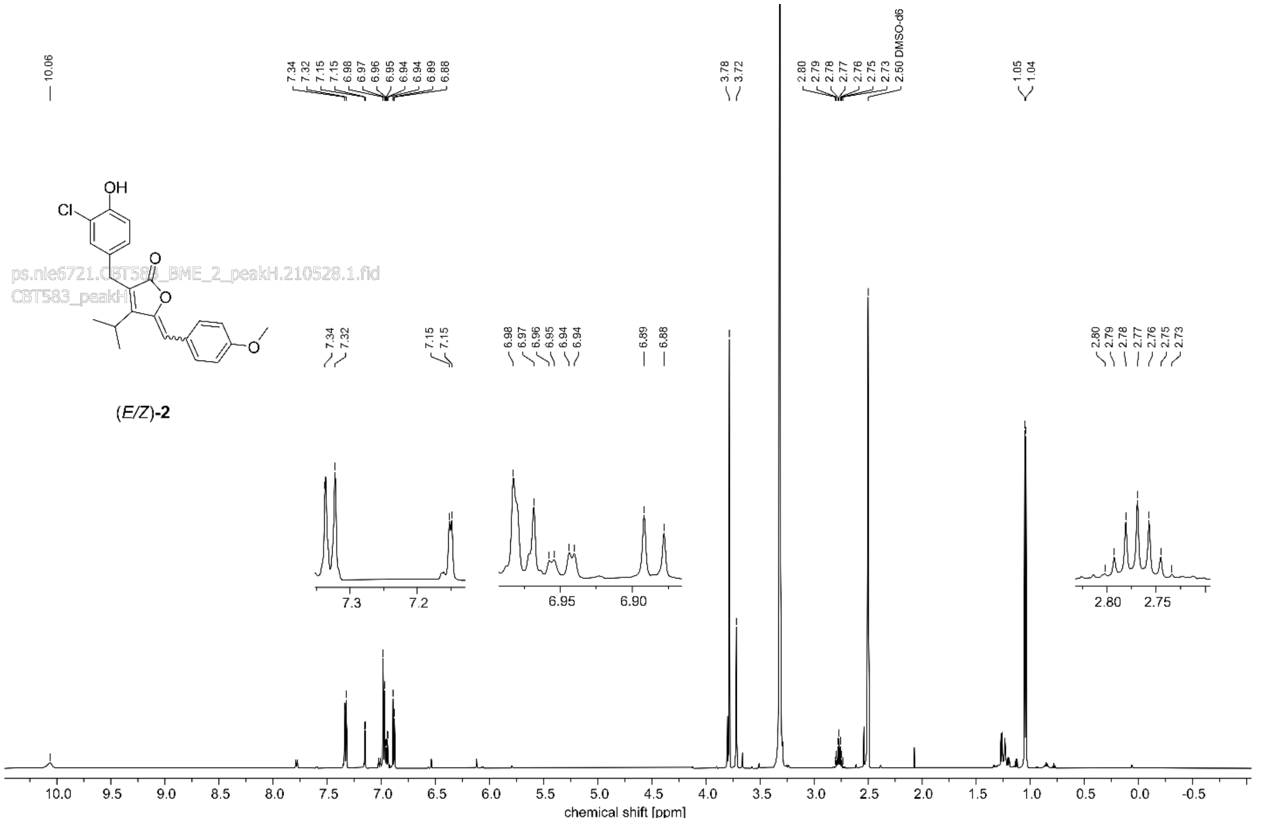

Figure S28. ^{1}H NMR spectrum (600 MHz) of (*E*)-**2** in DMSO-d_6. Due to swift isomerization, signals of (*Z*)-**2** are visible too. Insets show expanded regions of the spectrum from δ_H 7.1-7.4 ppm, δ_H 6.87-7.05 ppm, and δ_H 2.5-2.8 ppm.

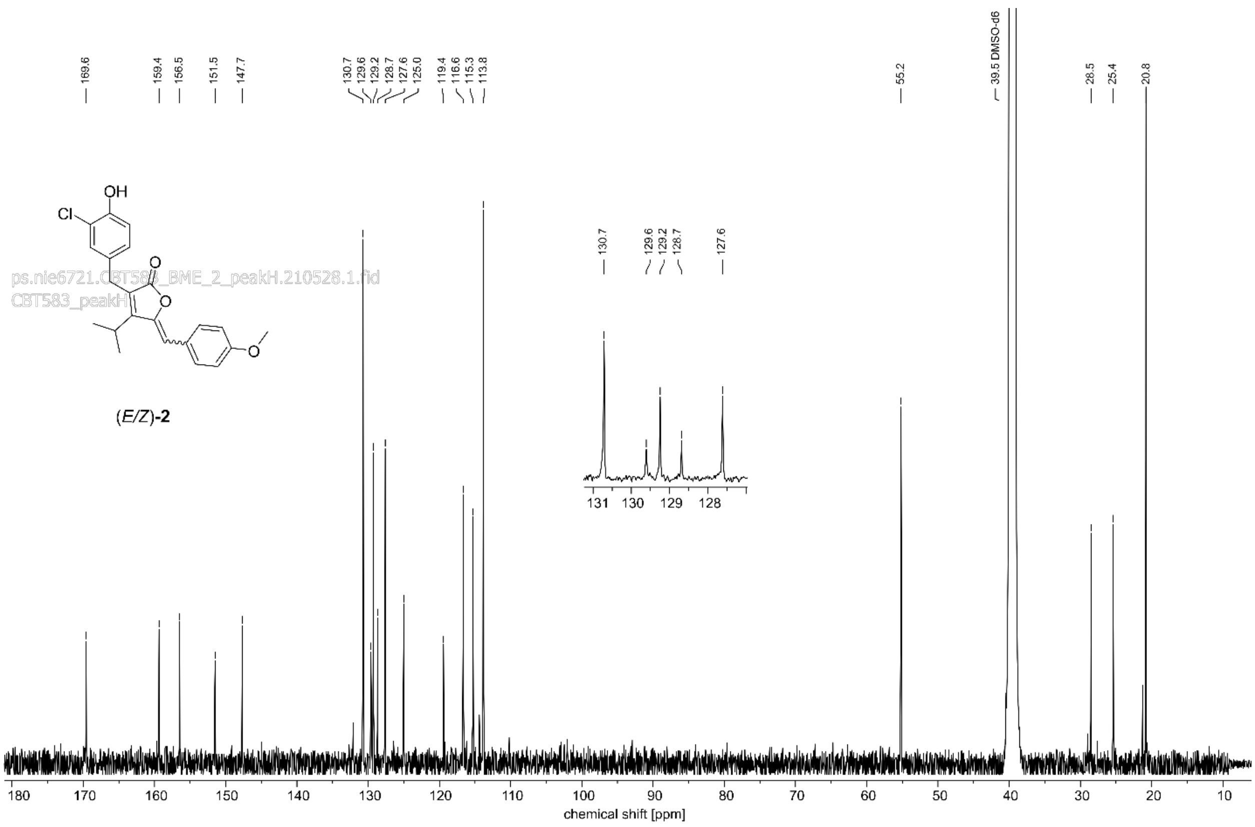

Figure S29. ^{13}C NMR spectrum (150 MHz) of (*E*)-**2** in DMSO-d_6. Inset schows expanded region of the spectrum from δ_C 127-131 ppm.

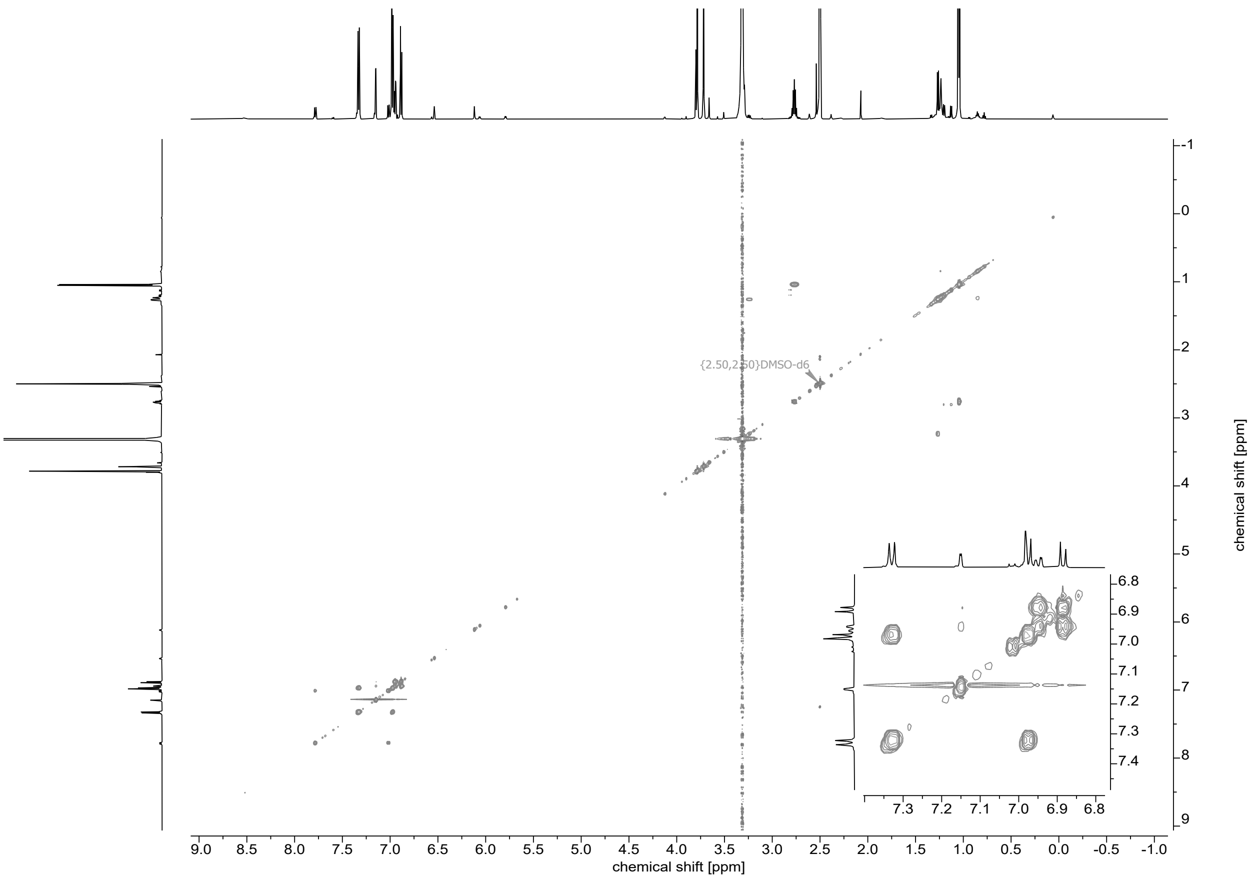

Figure S30. COSY NMR spectrum (600 MHz) of (*E*)-**2** in DMSO-d_6. Inset shows expanded region of the spectrum from δ_H 6.8-7.4 ppm.

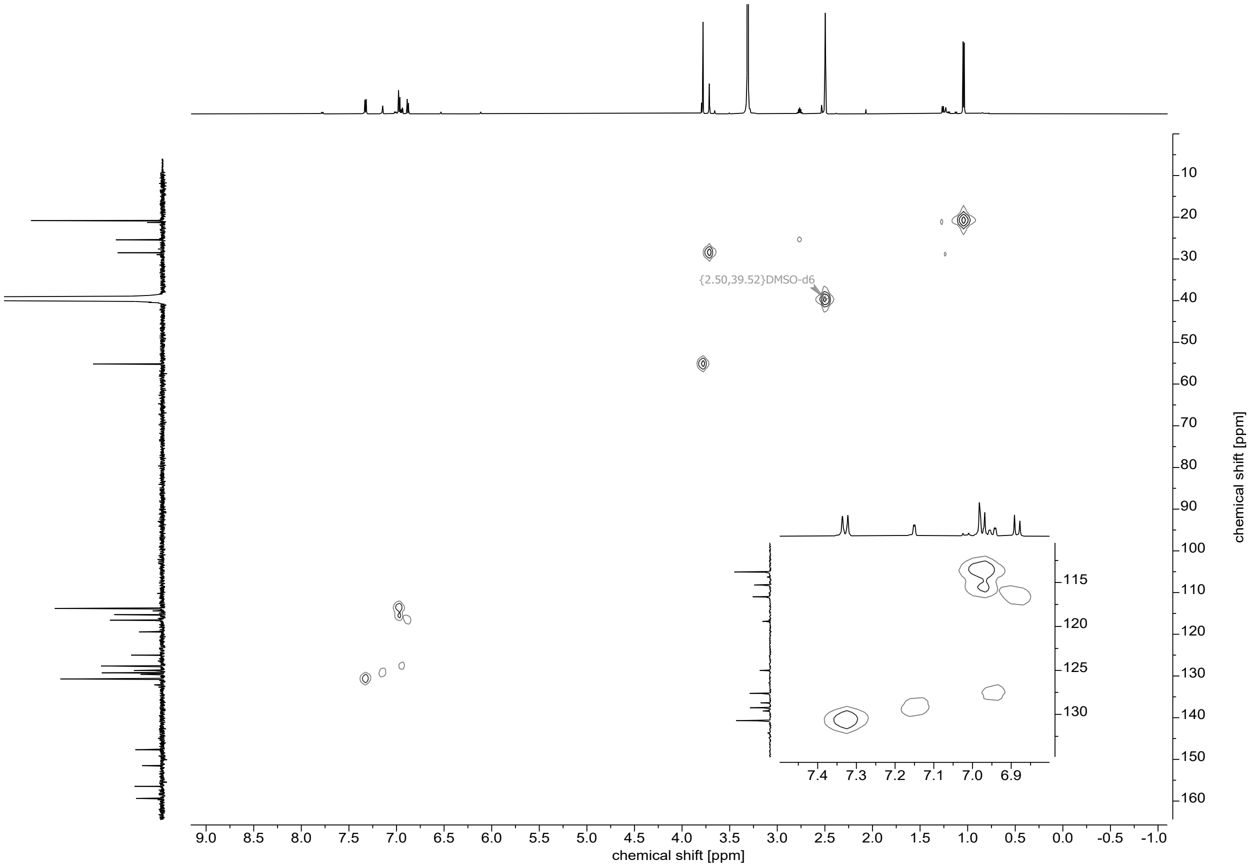

Figure S31. ^{13}C-HMQC NMR spectrum (600 MHz) of (*E*)-**2** in DMSO-*d*$_6$. Inset shows expanded region of the spectrum from δ_H 6.8-7.5 ppm and δ_C 112.5-132.5 ppm.

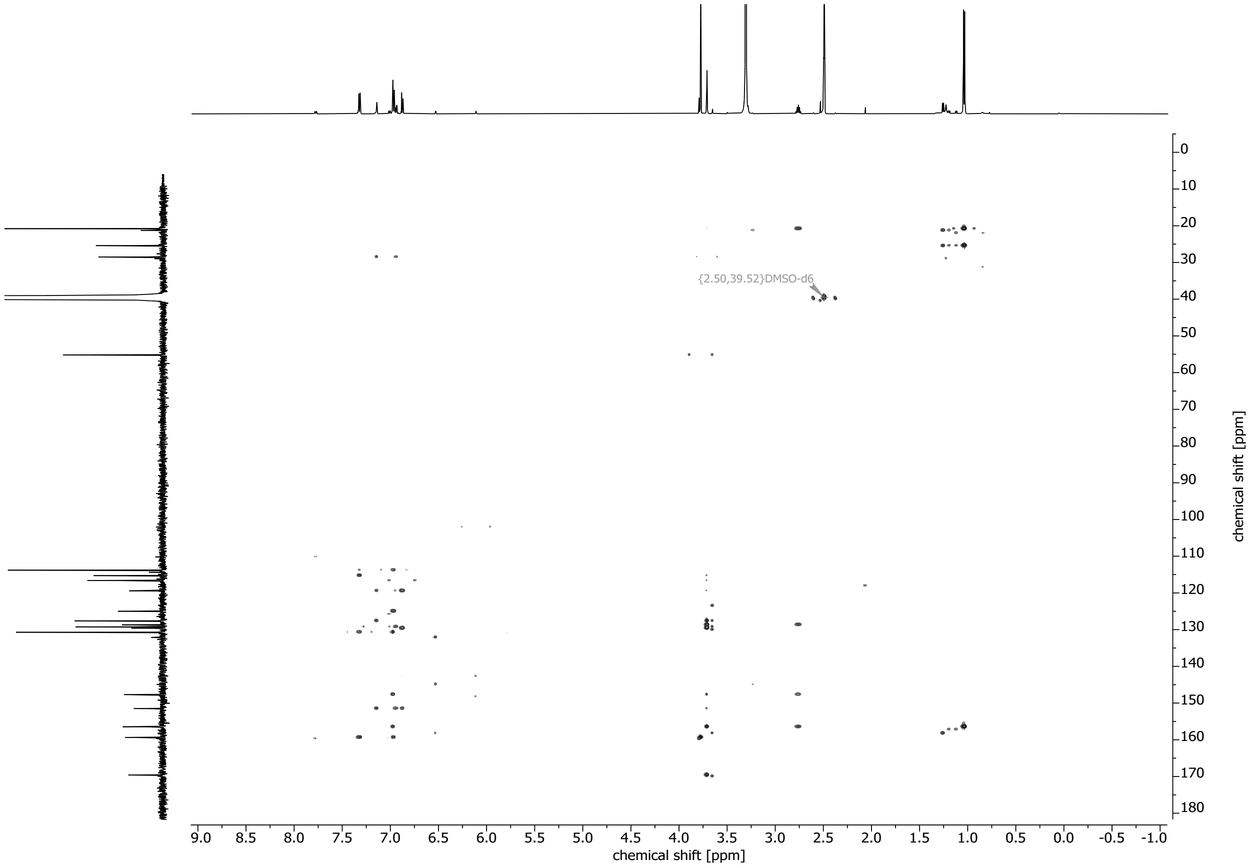

Figure S32. ^{13}C-HMBC NMR spectrum (600 MHz) of (*E*)-**2** in DMSO-*d*$_6$. Due to swift isomerization, signals of (*Z*)-**2** are visible too.

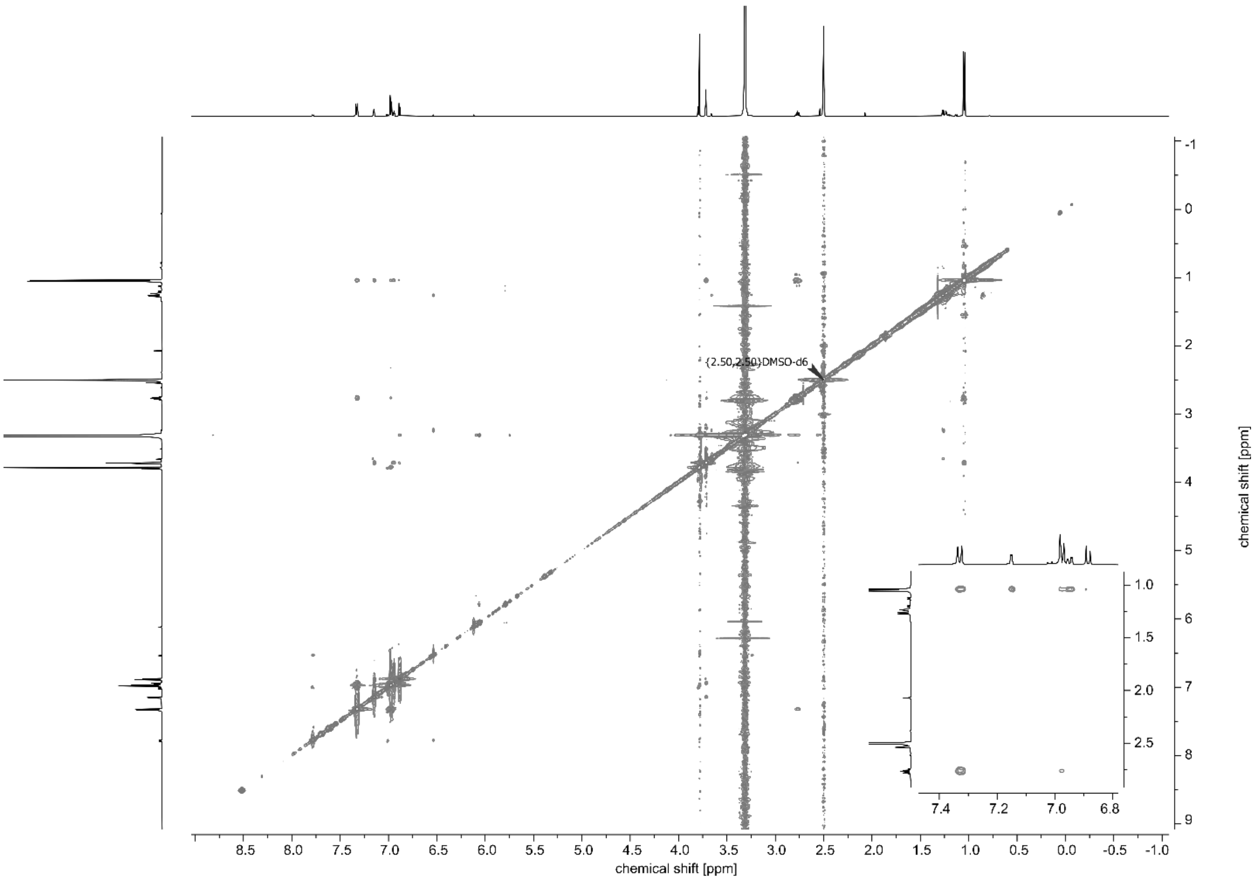

Figure S33. ROESY NMR spectrum (600 MHz) of (*E*)-**2** in DMSO-d_6. Inset shows expanded region of the spectrum from δ_H 6.8-7.4 ppm and δ_H 1.0-3.0 ppm, highlighting representative NOE correlations used to distinguish between the *E* and *Z* isomers.

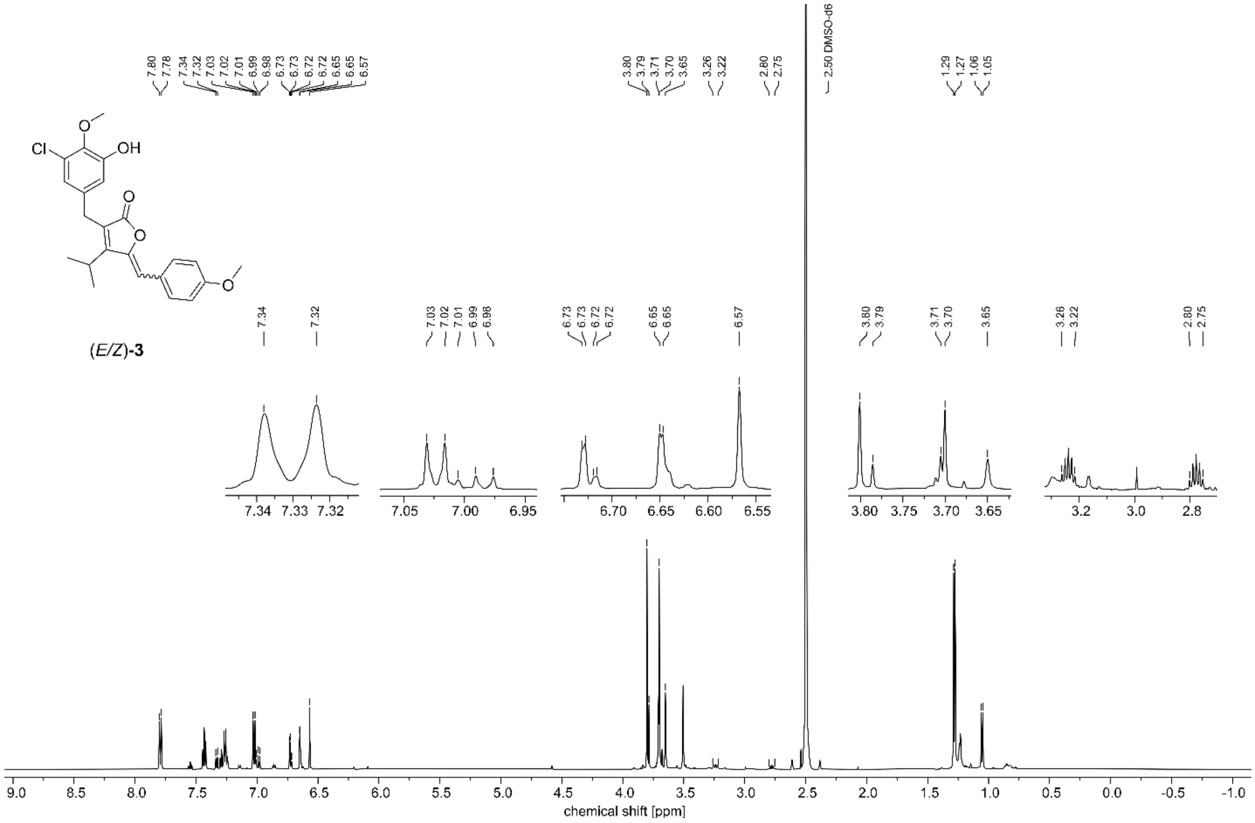

Figure S34. ^{1}H NMR spectrum (600 MHz) of (*Z*/*E*)-**3** in DMSO-d_6. Compounds *E*/*Z*-**3** were co-isolated, resulting in the coexistence of both isomers in the final NMR sample set. Insets show different expanded regions of the ^{1}H NMR spectrum.

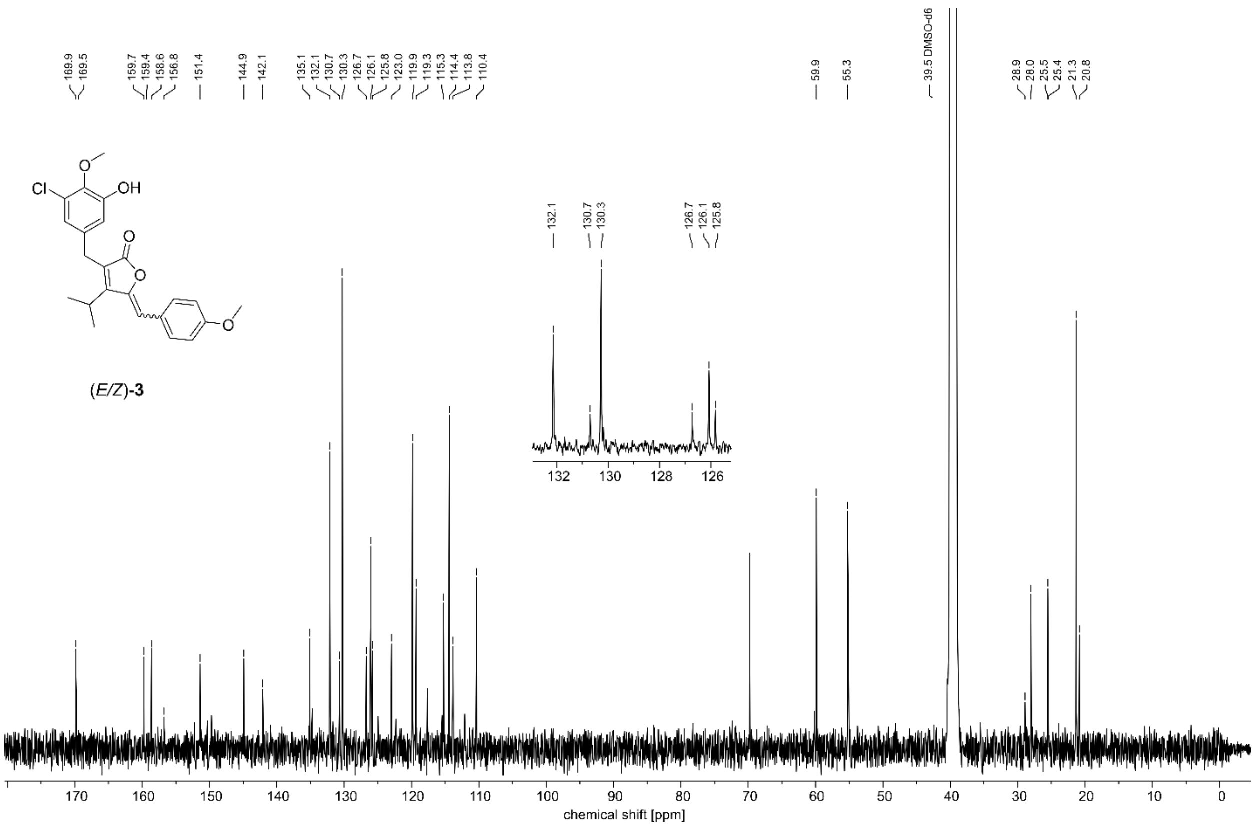

Figure S35. ^{13}C NMR spectrum (150 MHz) of (*E*/*Z*)-**3** in DMSO-d_6. Inset shows expanded region of the spectrum from δ_C 125-133 ppm.

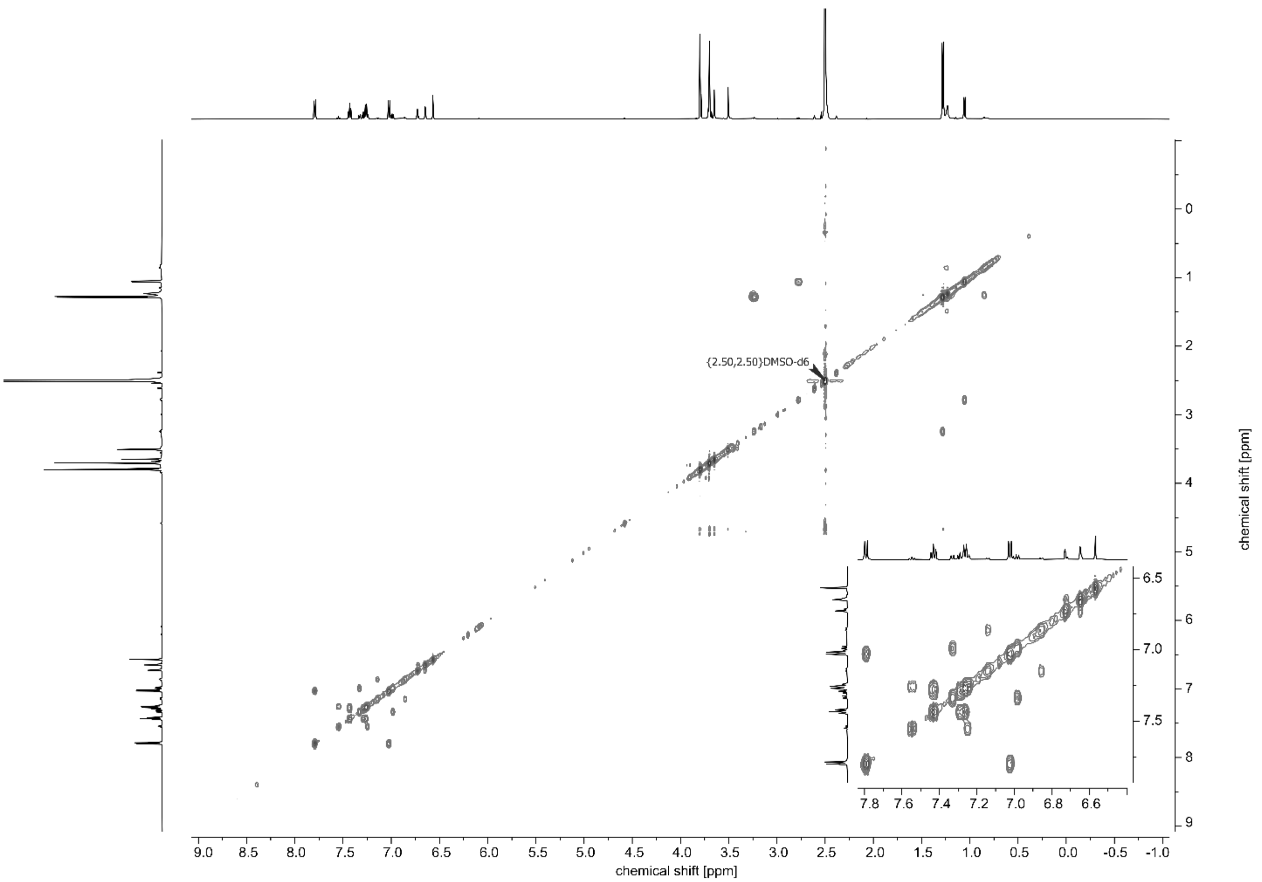

Figure S36. COSY NMR spectrum (600 MHz) of (*E*/*Z*)-**3** in DMSO-d_6. Inset shows expanded region of the spectrum from δ_H 6.5-8.0 ppm and δ_H 6.5-7.8 ppm.

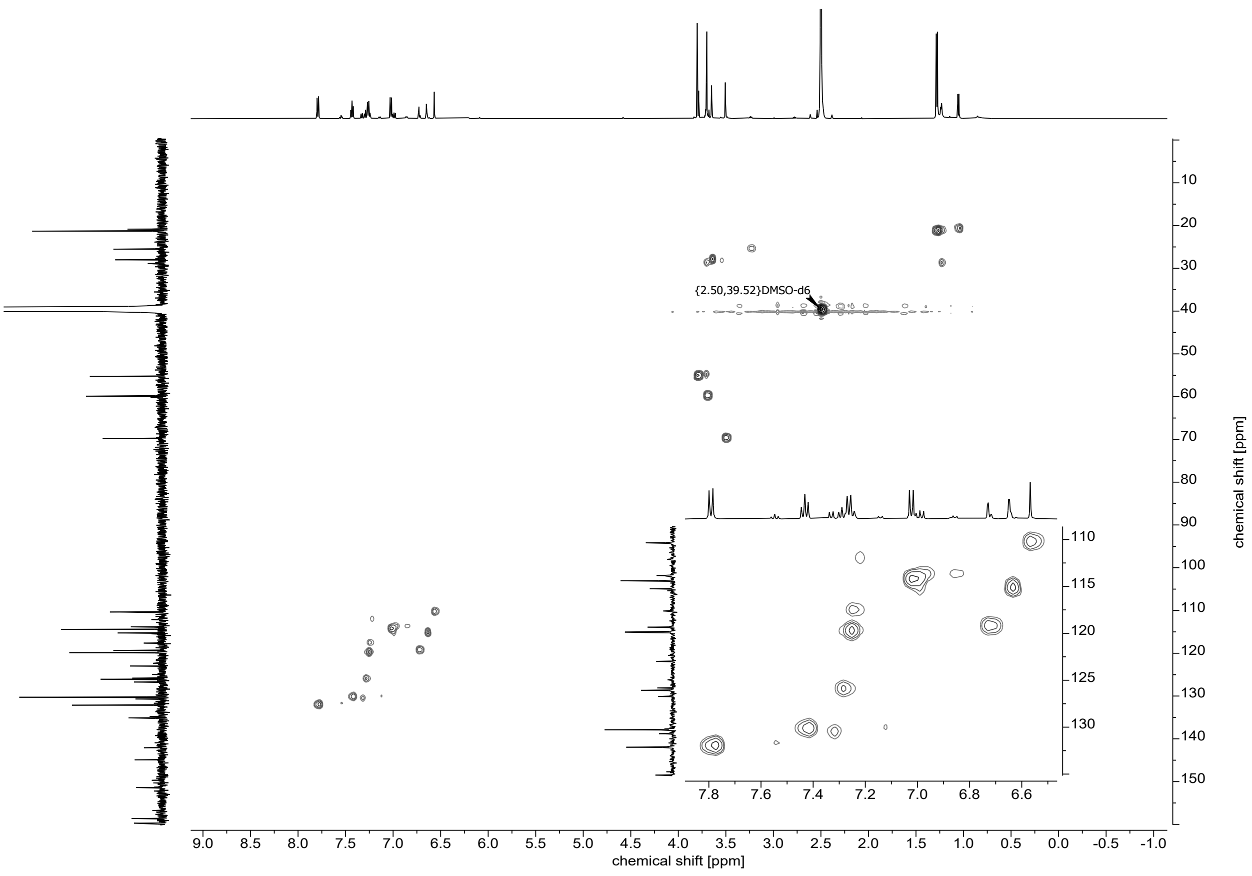

Figure S37. ^{13}C-HMQC NMR spectrum (600 MHz) of (*E*/*Z*)-**3** in DMSO-d_6. Inset shows expanded region of the spectrum from δ_C 110-140 ppm and δ_H 6.5-7.9 ppm.

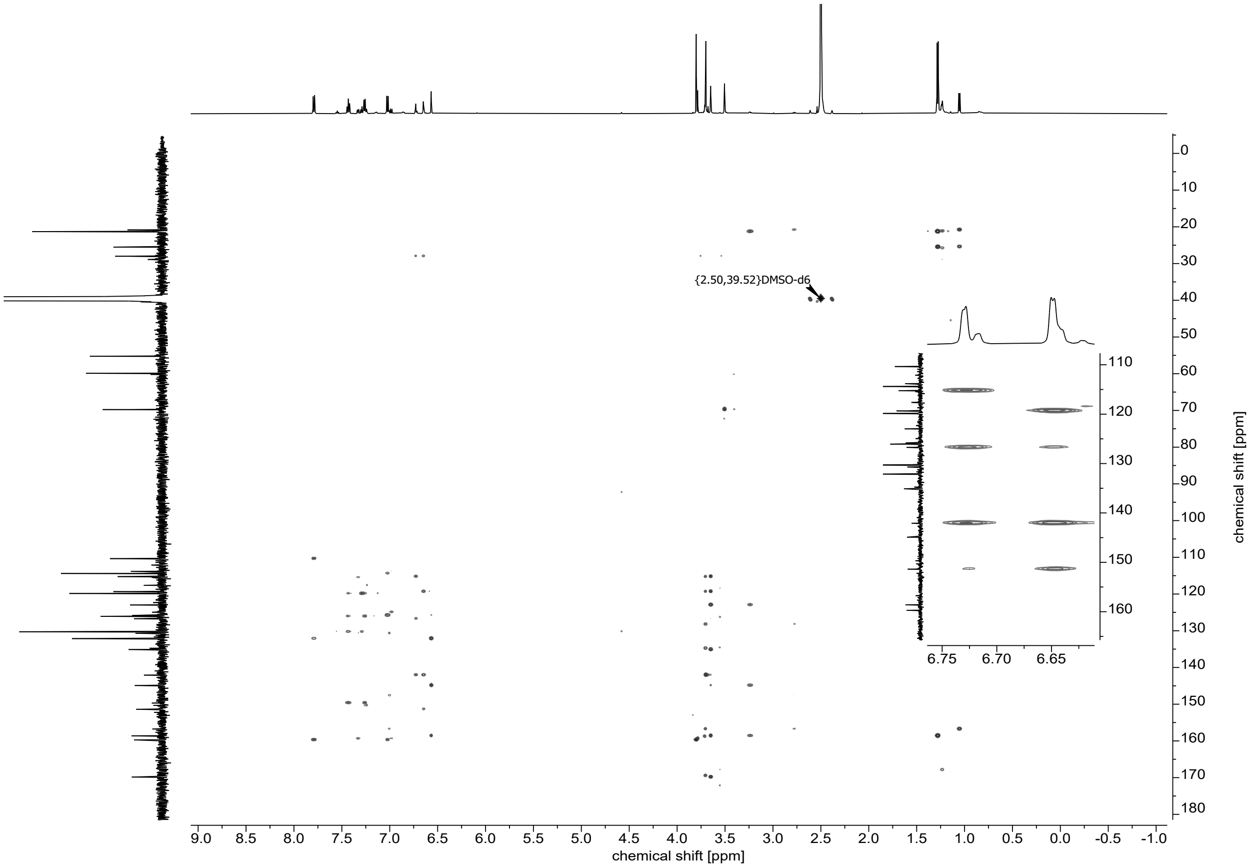

Figure S38. ^{13}C-HMBC NMR spectrum (600 MHz) of (*E*/*Z*)-**3** in DMSO-d_6. Inset shows expanded region of the spectrum from δ_C 110-165 ppm and δ_H 6.60-6.75 ppm.

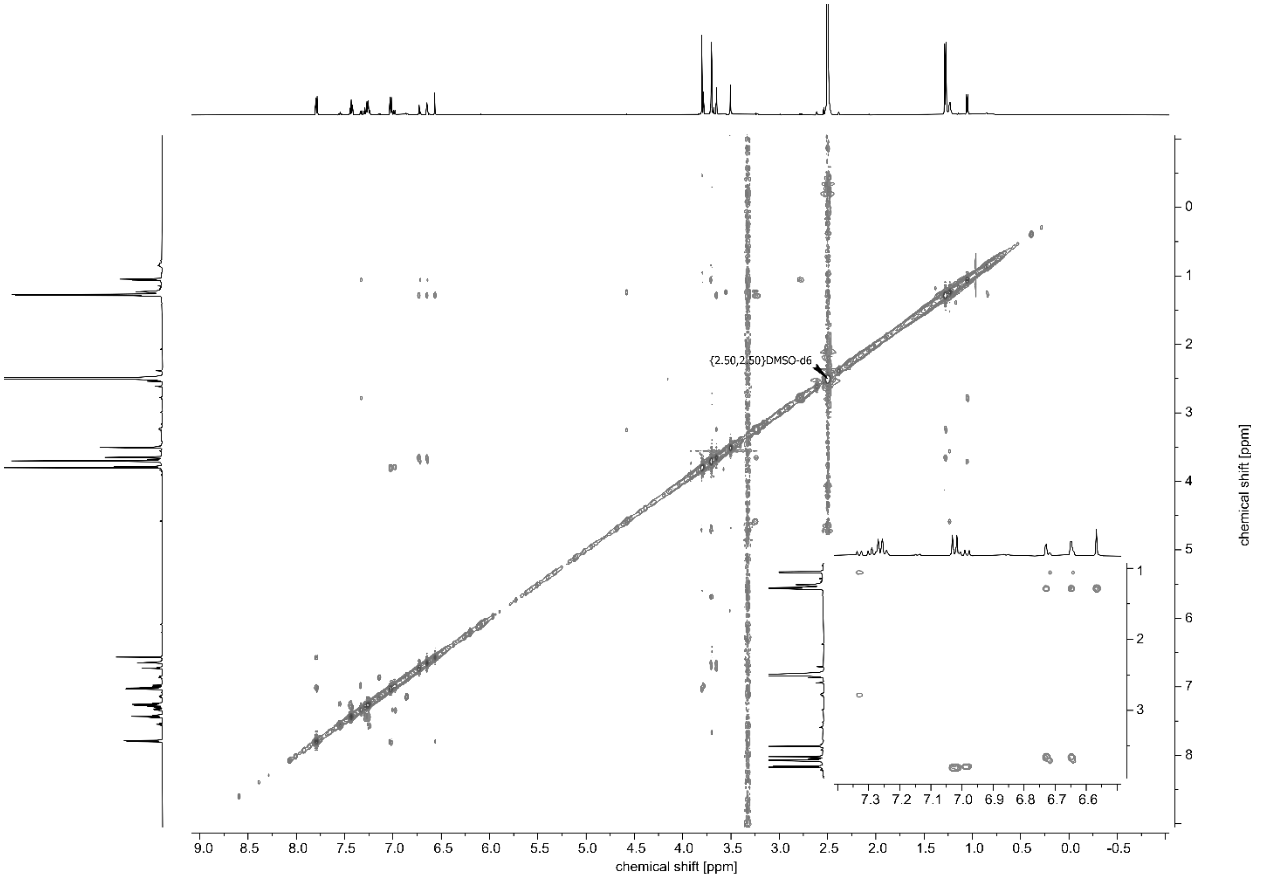

Figure S39. NOESY NMR spectrum (600 MHz) of (*E*/*Z*)-**3** in DMSO-d_6. Inset shows expanded regions of the spectrum from δ_H 6.5-7.4 ppm and δ_H 1.0-5.0 ppm, highlighting representative NOE correlations used to distinguish between the *E* and *Z* isomers.

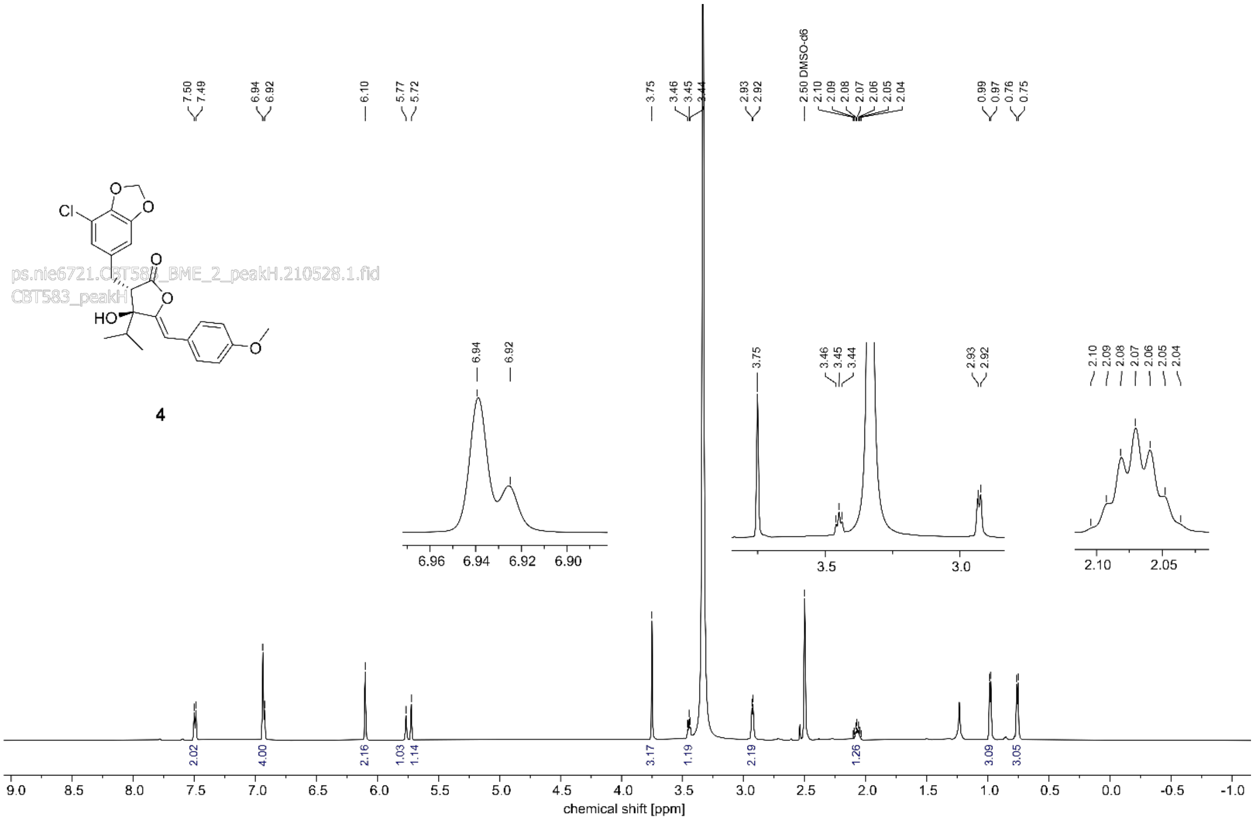

Figure S40. ^{1}H NMR spectrum (600 MHz) of **4** in DMSO-d_6. One inset shows an expanded region of overlapping aromatic signals (δ_H 6.89-6.97 ppm); HMQC data confirm that the four protons in this region are attached to distinct carbon atoms. Two additional insets highlight further details at δ_H 2.90-3.80 ppm and δ_H 2.00-2.10 ppm.

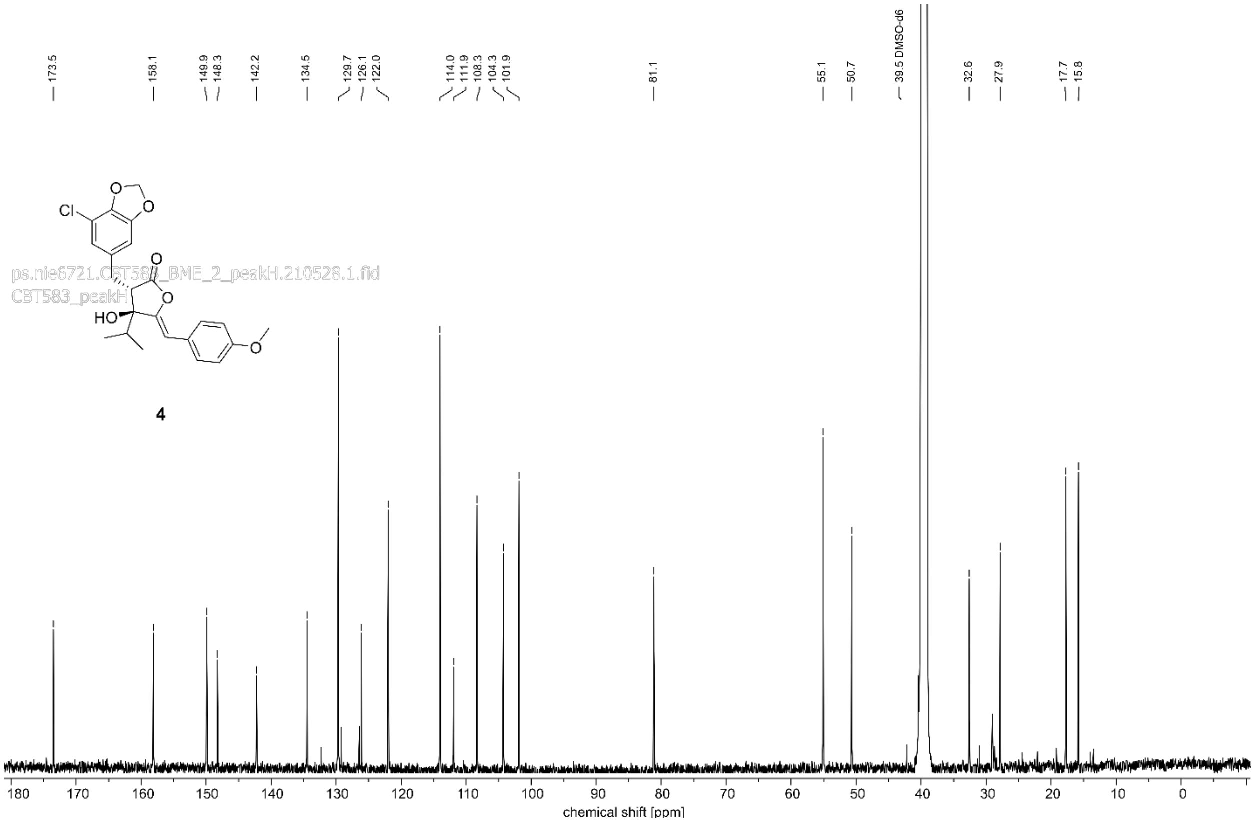

Figure S41. ^{13}C NMR spectrum (150 MHz) of **4** in DMSO-d_6.

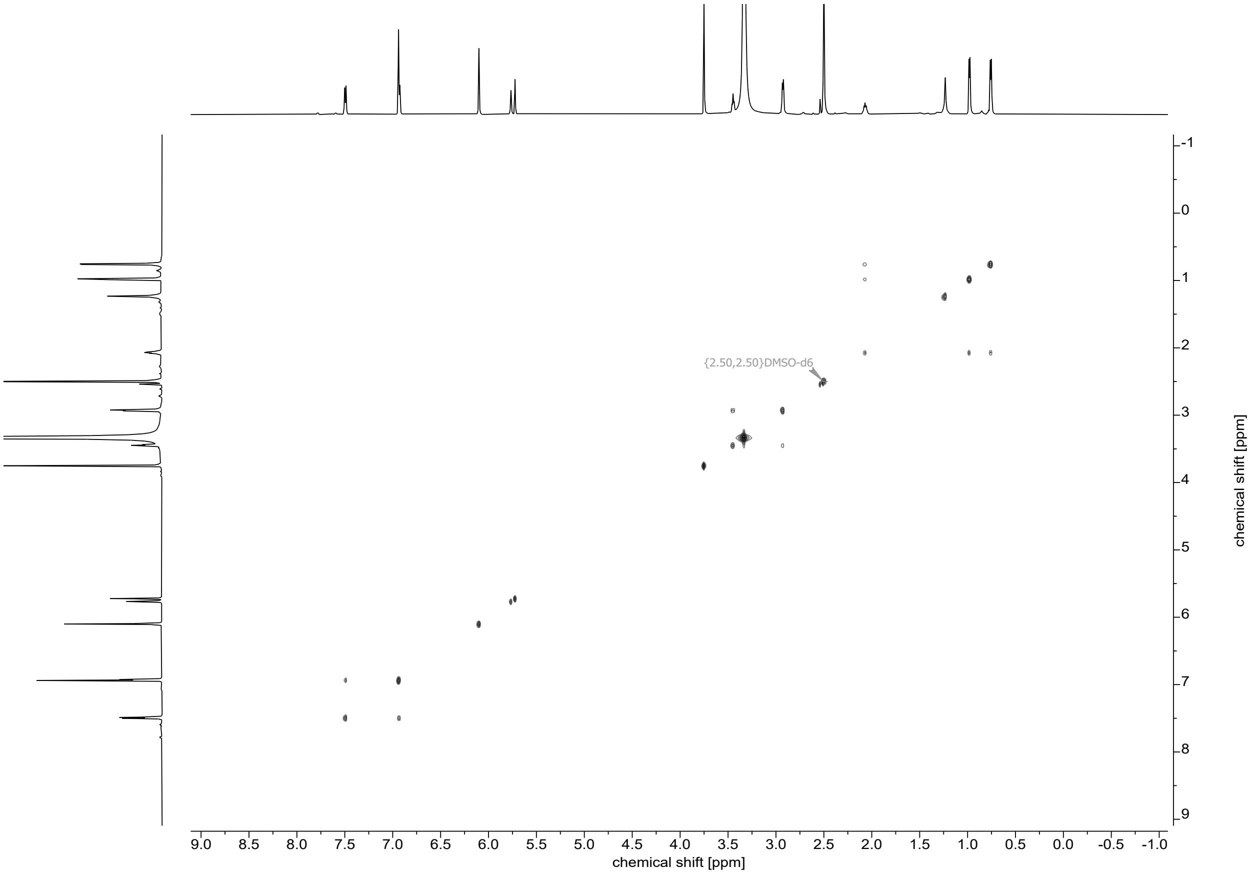

Figure S42. COSY NMR spectrum (600 MHz) of **4** in DMSO-d_6.

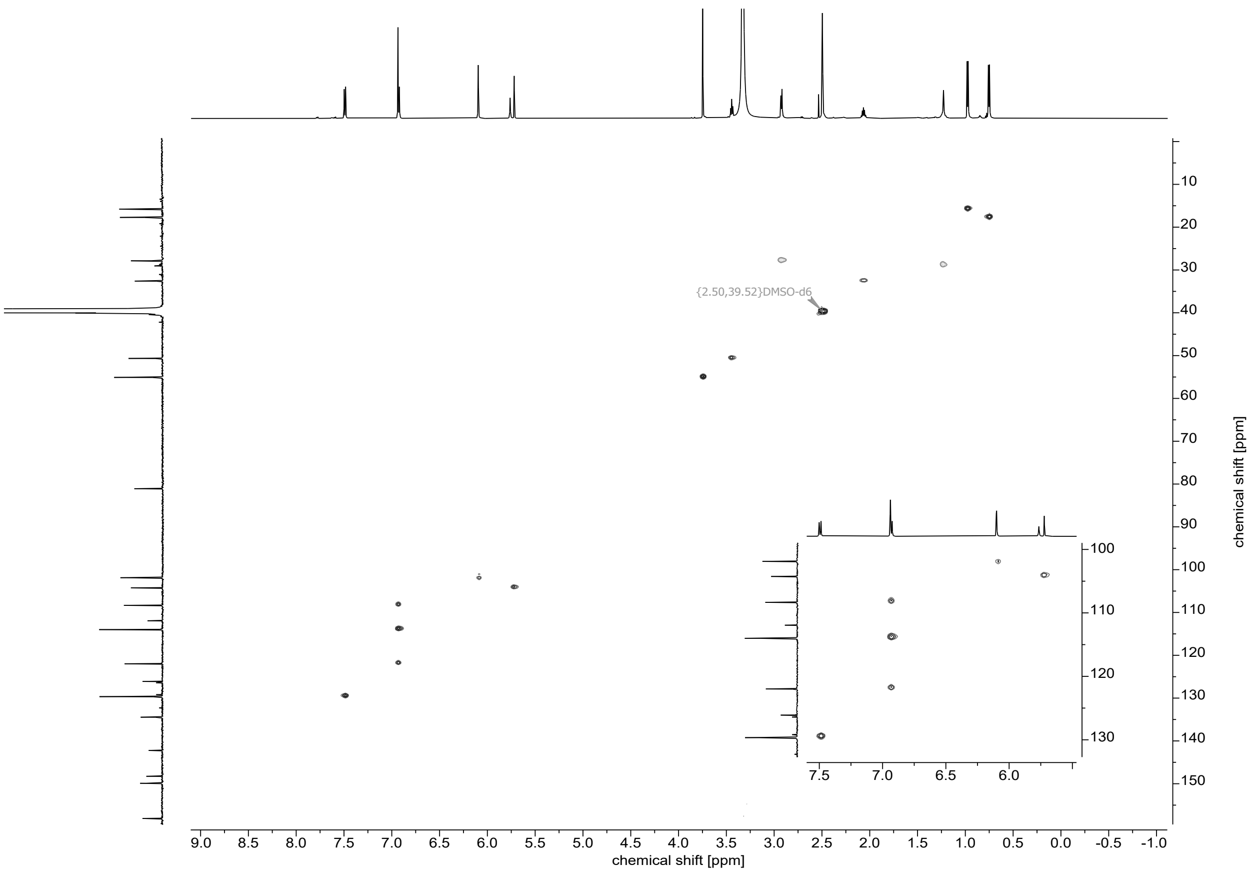

Figure S43. ^{13}C-HSQC NMR spectrum (600 MHz) of **4** in DMSO-d_6. Inset shows expanded regions of the spectrum from δ_H 5.5-7.5 ppm and δ_C 100.0-130.0 ppm, highlighting that the four protons in this region are attached to distinct carbon atoms.

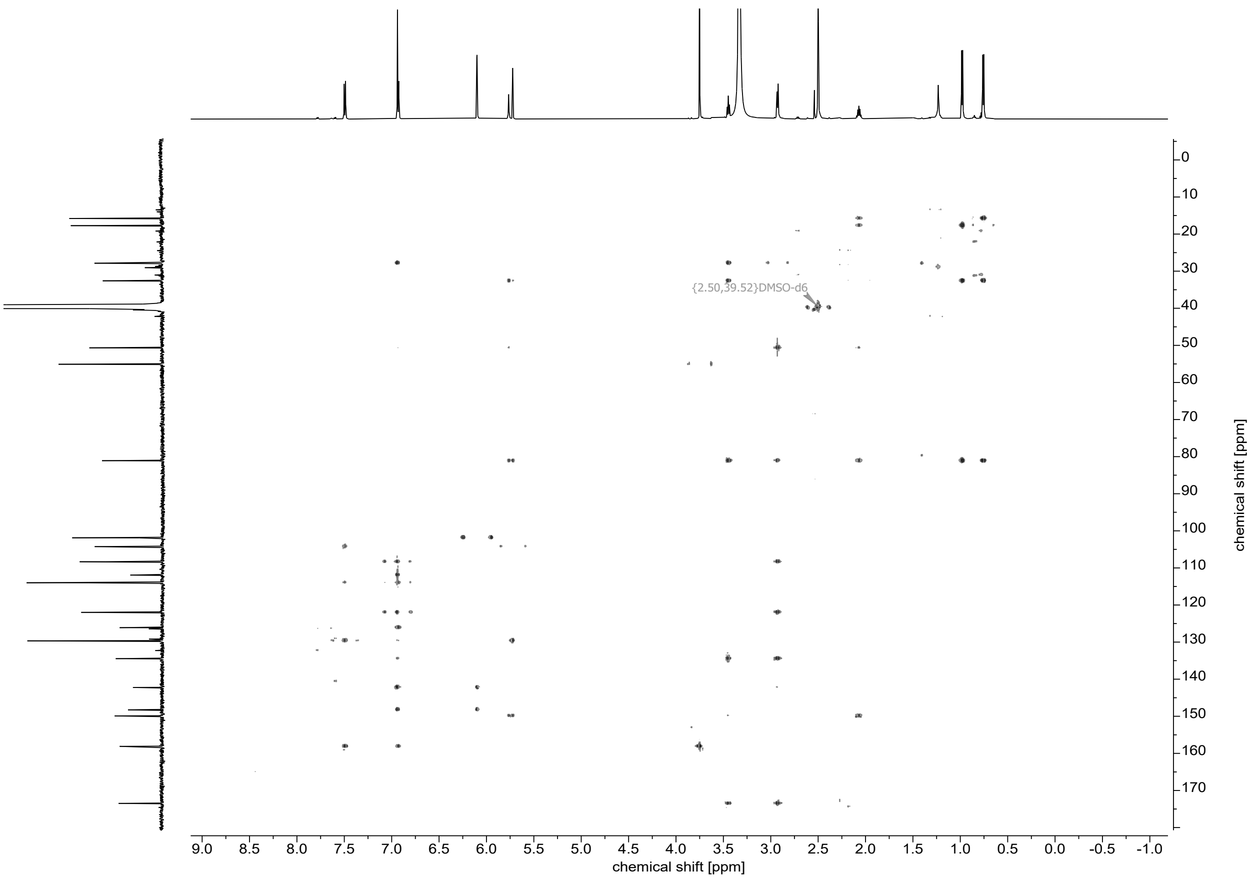

Figure S44.^{13}C-HMBC NMR spectrum (600 MHz) of **4** in DMSO-*d*₆.

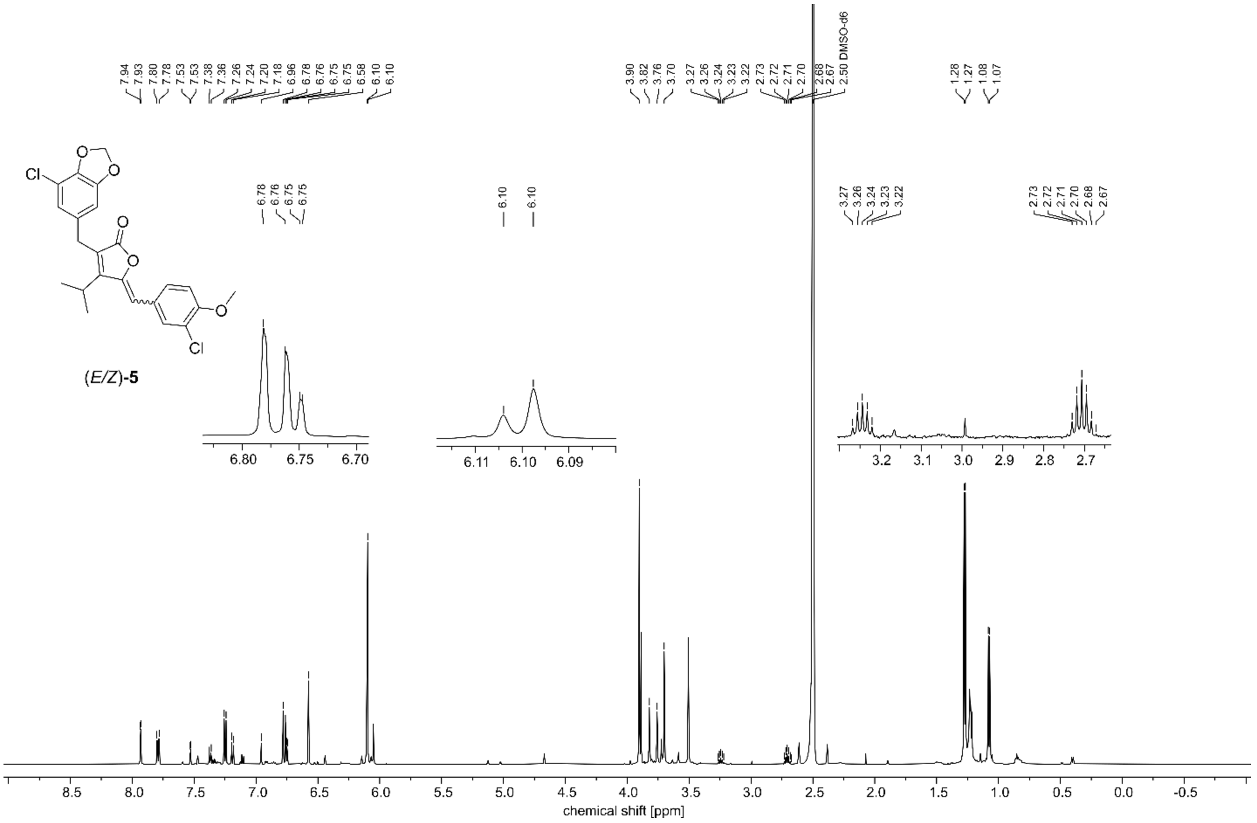

Figure S45. ^{1}H NMR spectrum (600 MHz) of (*Z*/*E*)-**5** in DMSO-d_6. Compounds *E*/*Z*-**5** were co-isolated, resulting in the coexistence of both isomers in the final NMR sample set. Insets show expanded regions of the spectrum from δ_H 6.7-6.8 ppm, δ_H 6.9-6.11 ppm, and δ_H 2.65-3.3 ppm.

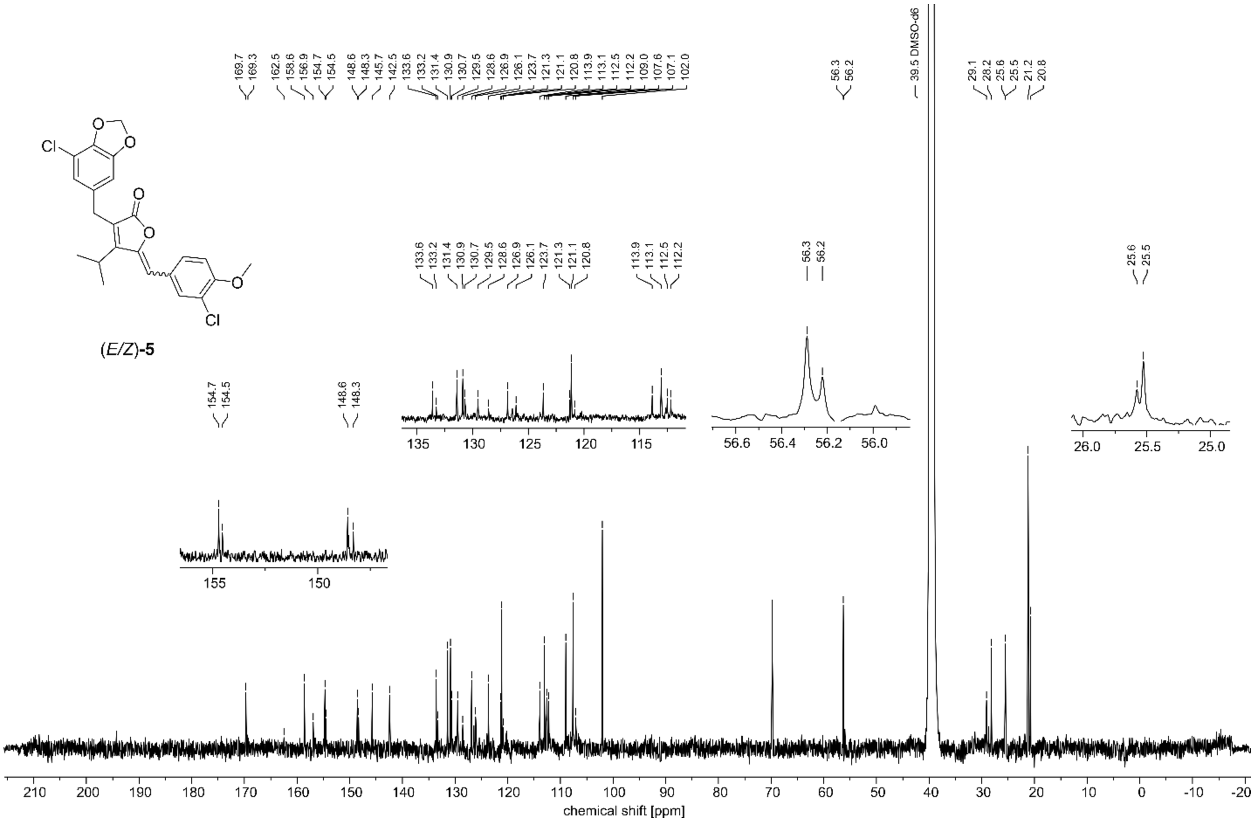

Figure S46. ^{13}C NMR spectrum (150 MHz) of (*E*/*Z*)-**5** in DMSO-d_6. Insets show expanded regions of the spectrum from δ_C 146-157 ppm, δ_C 111-135 ppm, δ_C 56.0-56.6 ppm, and δ_C 25.0-26.0 ppm.

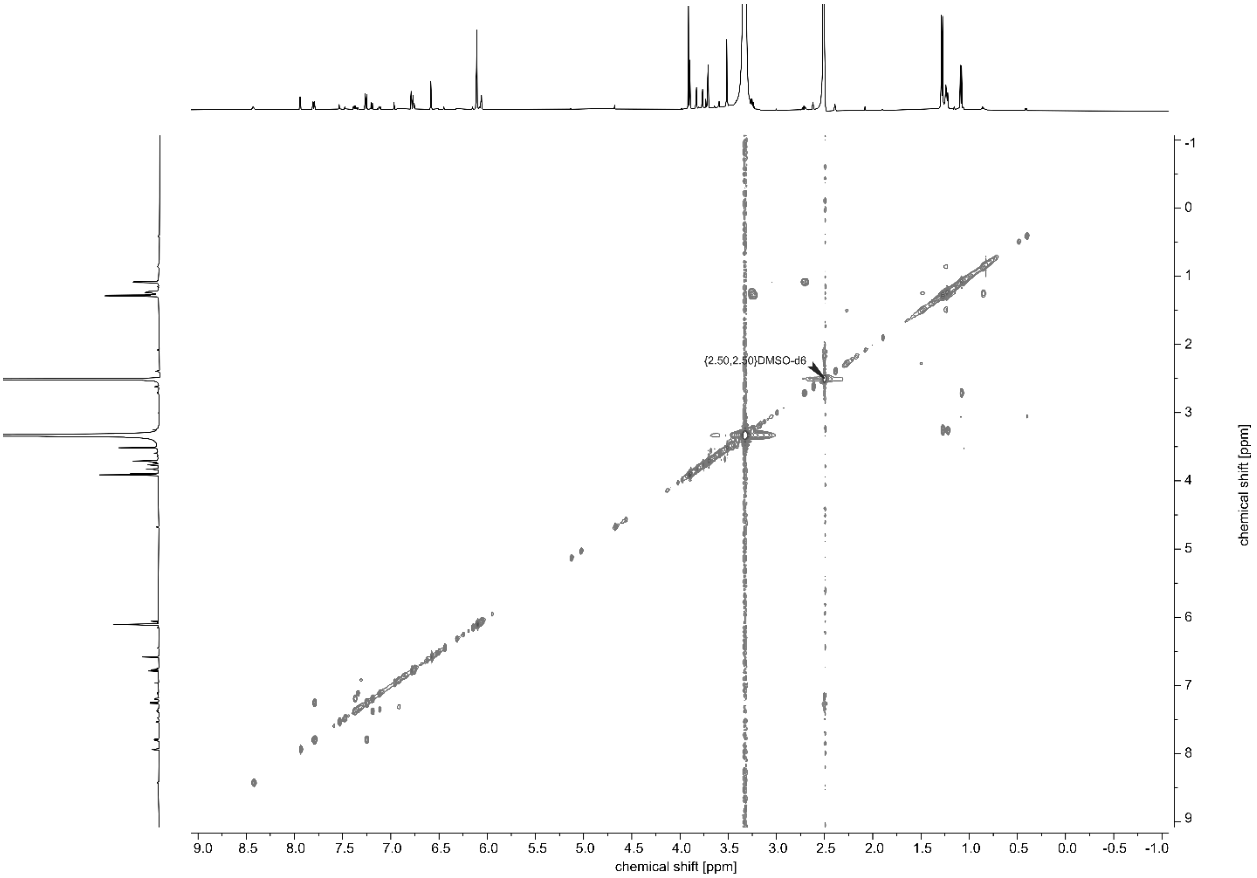

Figure S47. COSY NMR spectrum (600 MHz) of (*E*/*Z*)-**5** in DMSO-d_6.

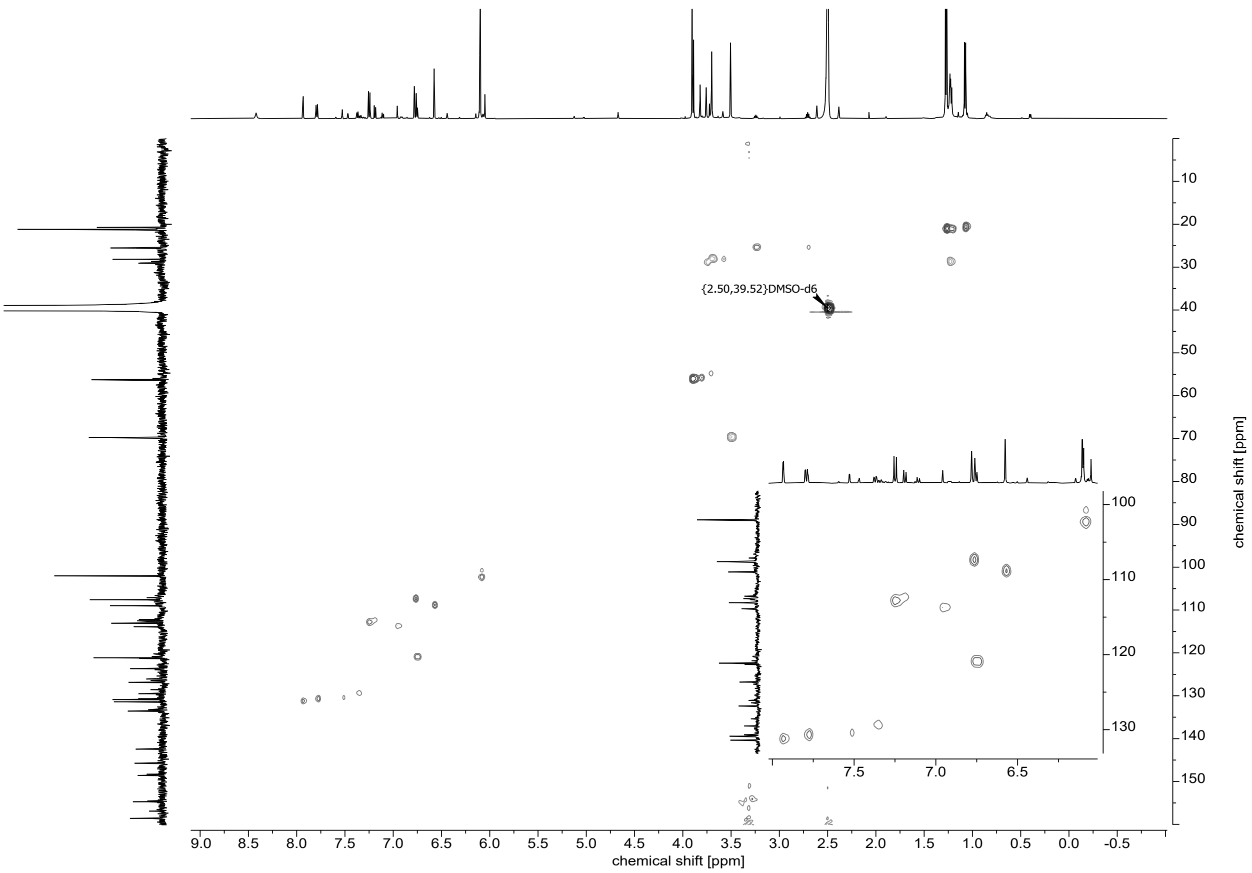

Figure S48. ^{13}C-HSQC NMR spectrum (600 MHz) of (*E*/*Z*)-**5** in DMSO-d_6. Inset shows expanded regions of the spectrum from δ_H 6.0-8.0 ppm, and δ_C 100-135 ppm.

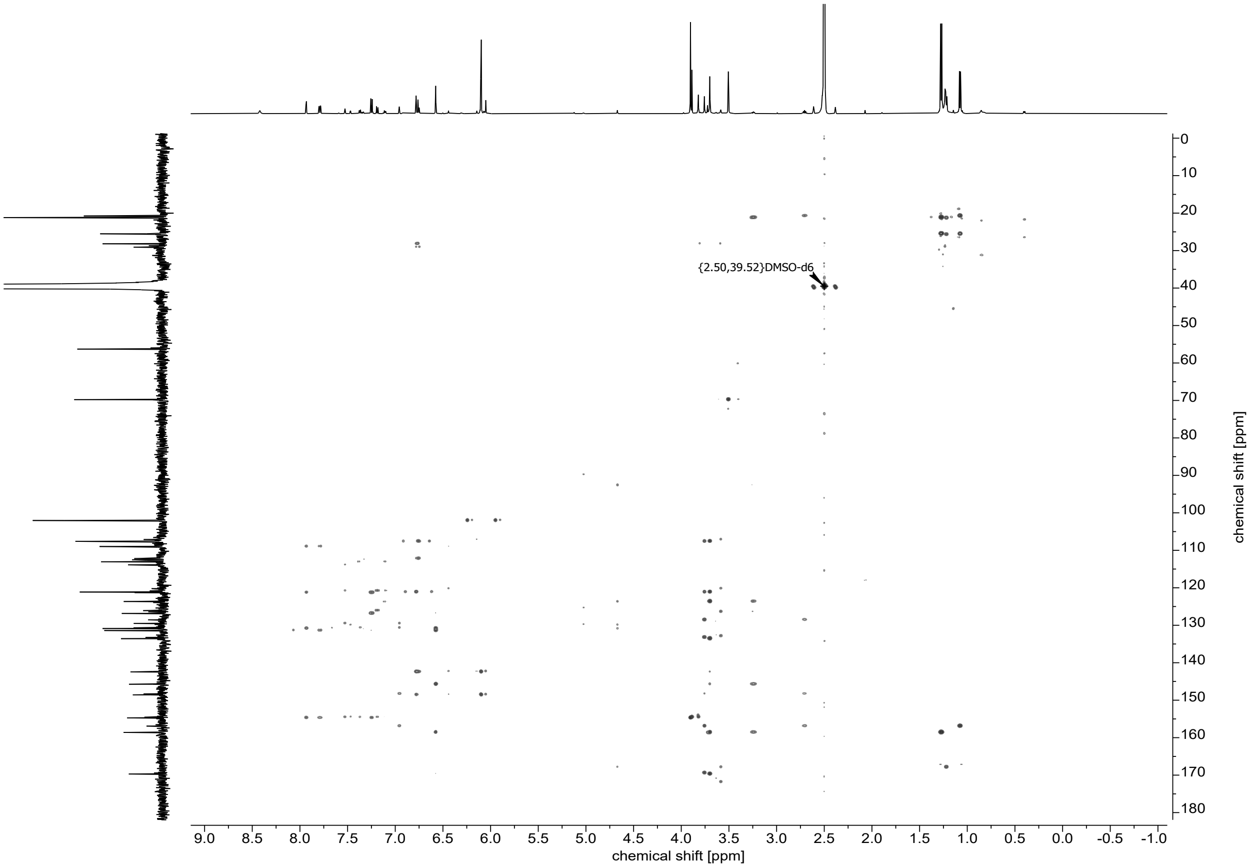

Figure S49. ^{13}C-HMBC NMR spectrum (600 MHz) of (*E*/*Z*)-**5** in DMSO-d_6.

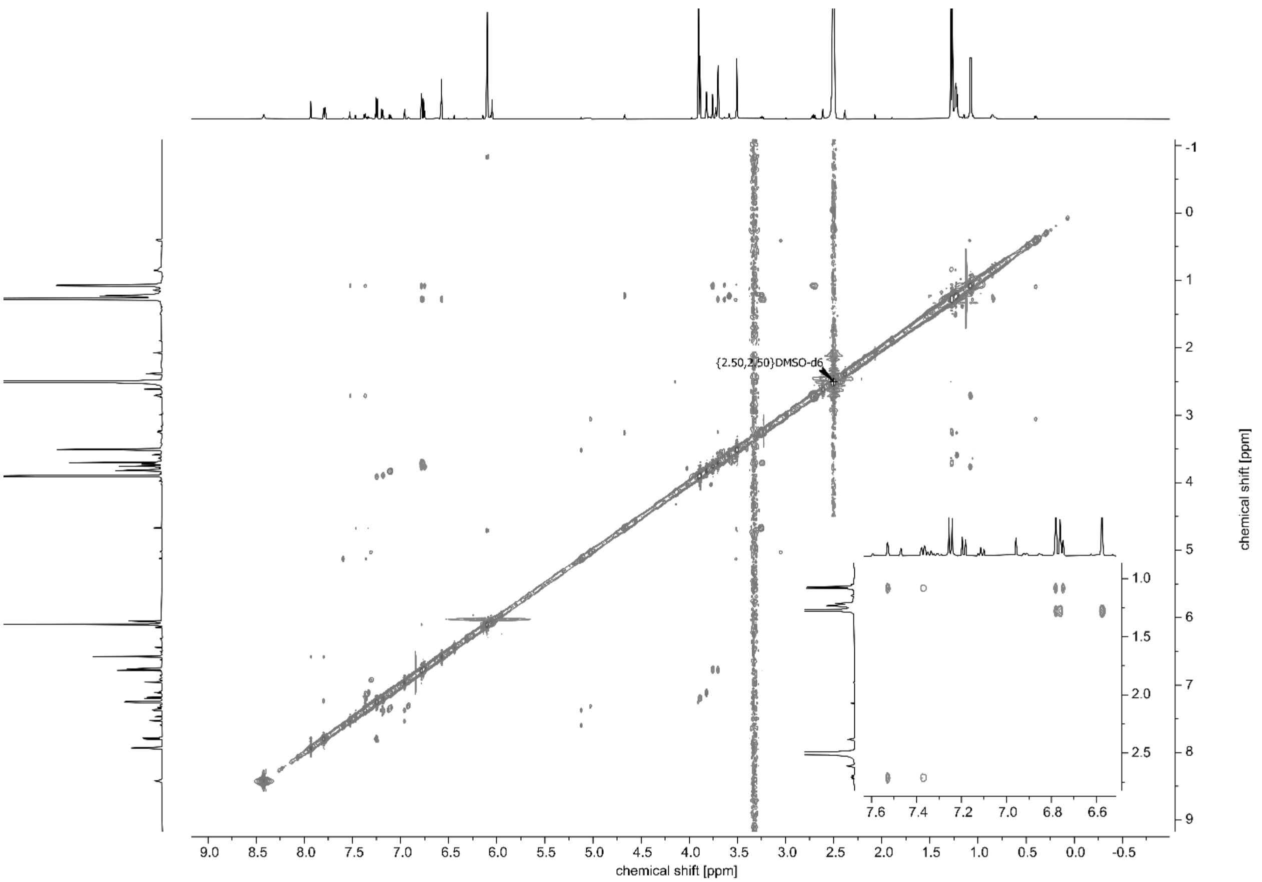

Figure S50. NOESY NMR spectrum (600 MHz) of (*E*/*Z*)-**5** in DMSO-d_6. Inset shows expanded regions of the spectrum from δ_H 6.5-7.6 ppm and δ_H 1.0-2.8 ppm, highlighting representative NOE correlations used to distinguish between the *E* and *Z* isomers.

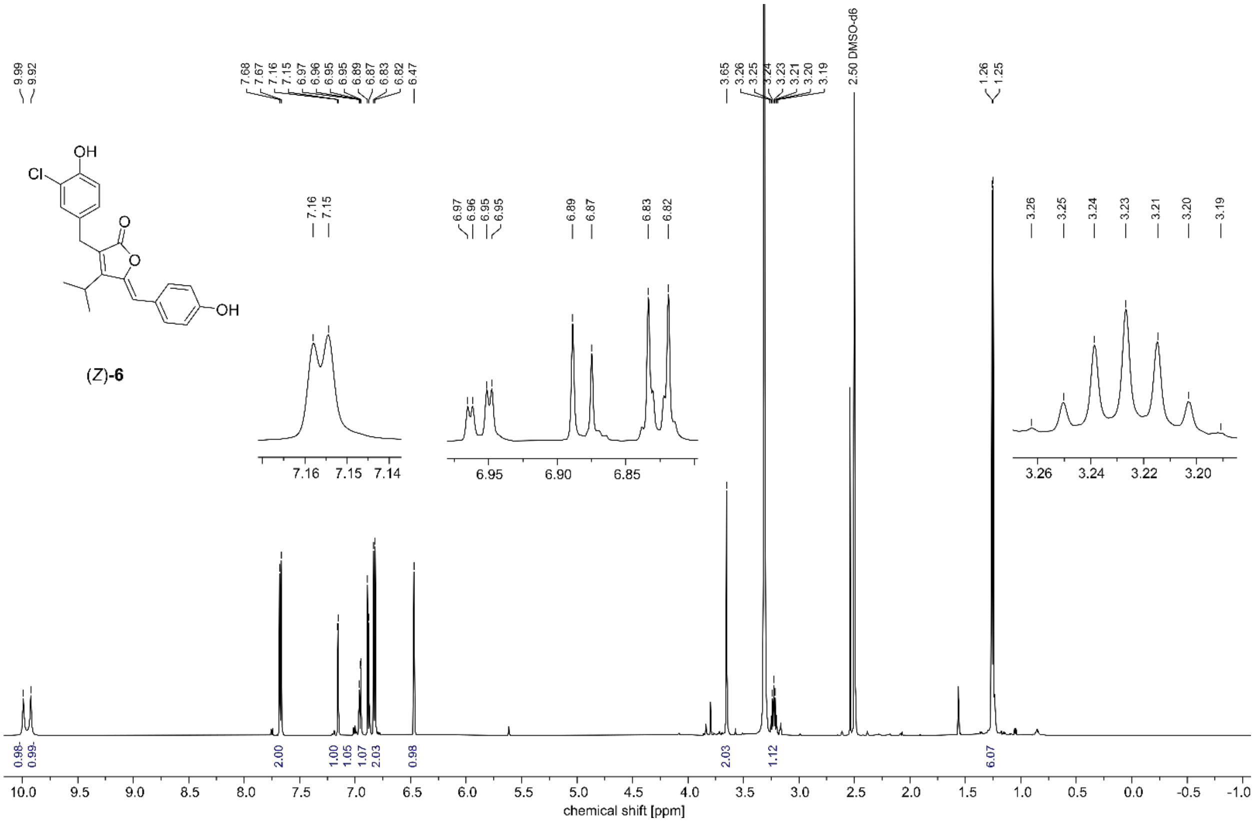

Figure S51. ^{1}H NMR spectrum (600 MHz) of *Z*-**6** in DMSO-d_6. Insets show expanded regions of the spectrum from δ_H 7.14-7.18 ppm, δ_H 6.80-6.98 ppm, and δ_H 3.19-3.26 ppm.

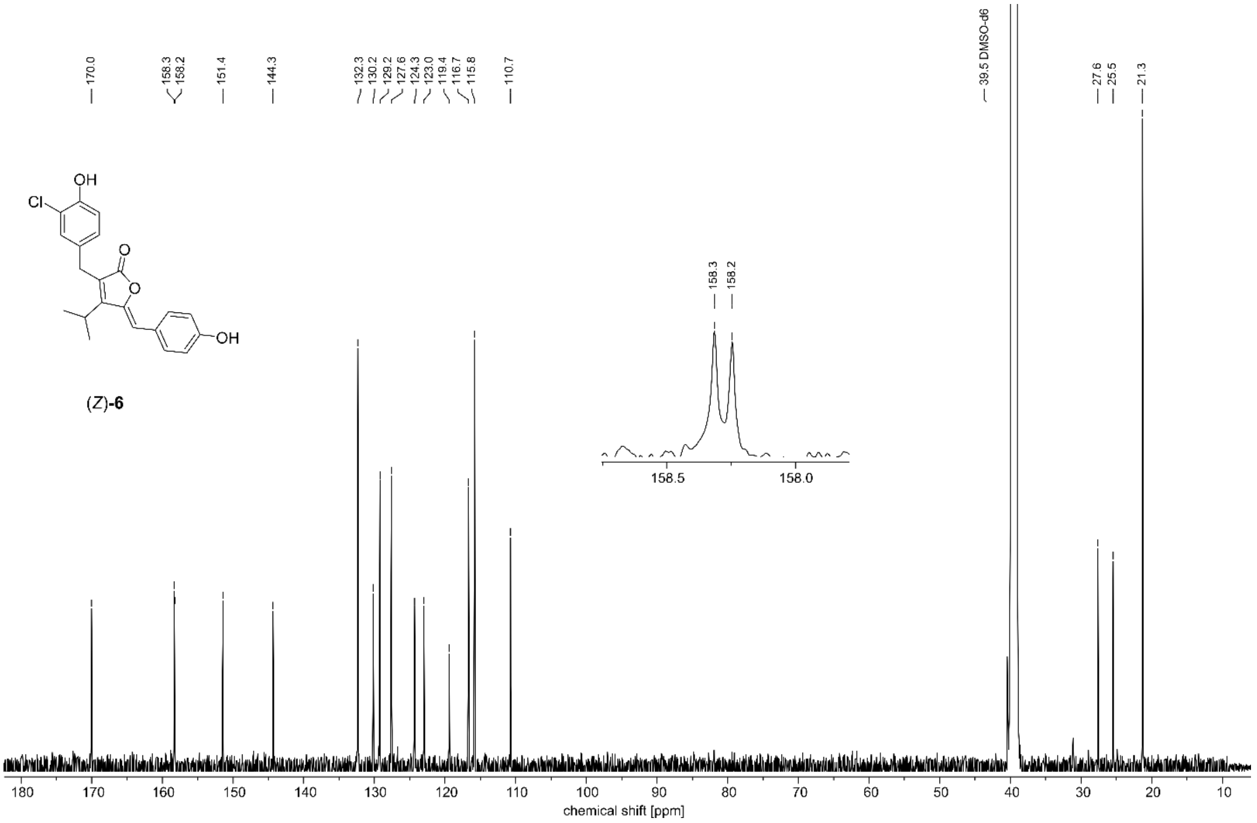

Figure S52. ^{13}C NMR spectrum (150 MHz) of *Z*-**6** in DMSO-d_6. Inset shows expanded region of the spectrum from δ_C 158.0-158.5 ppm.

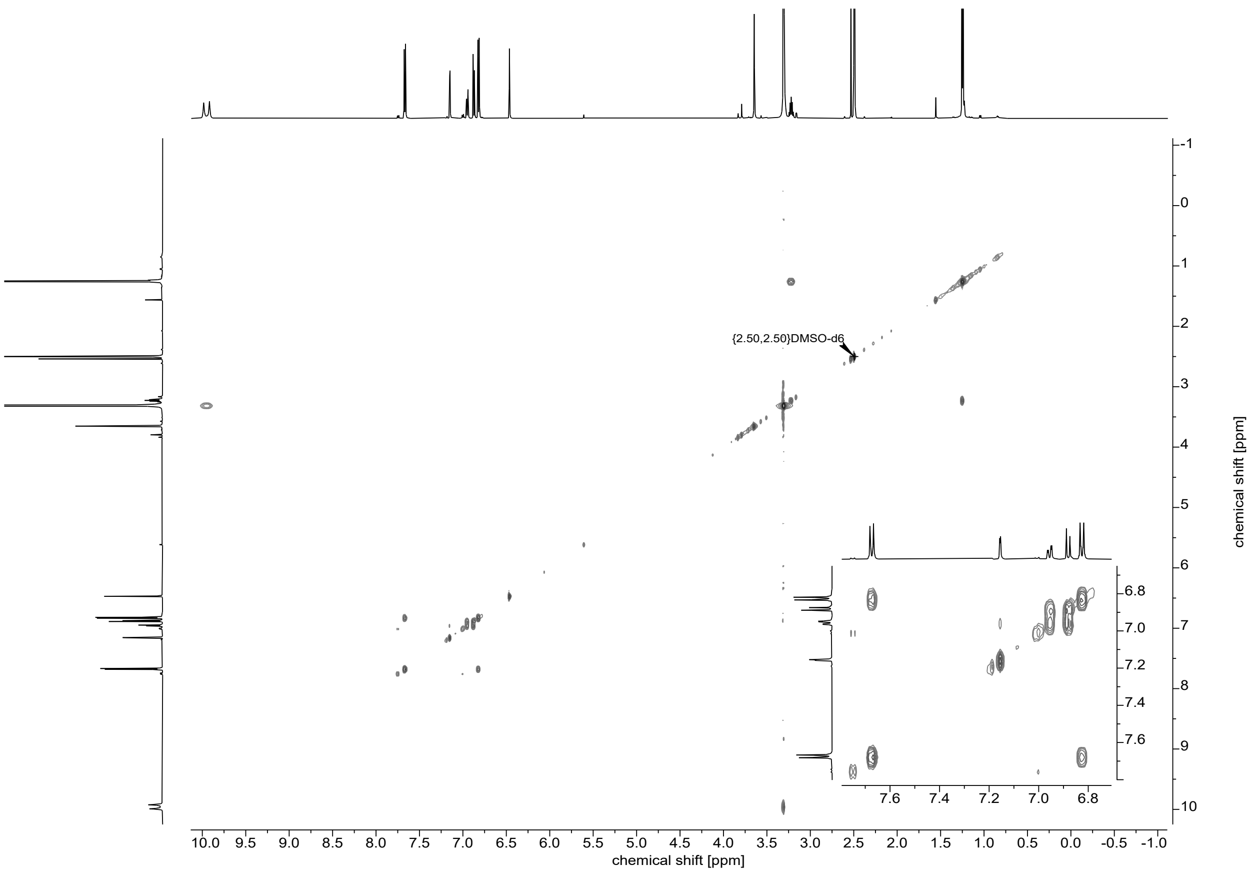

Figure S53. COSY NMR spectrum (600 MHz) of *Z*-**6** in DMSO-d_6. Inset shows expanded region of the spectrum from δ_H 6.7-7.8 ppm and δ_H 6.8-7.8 ppm.

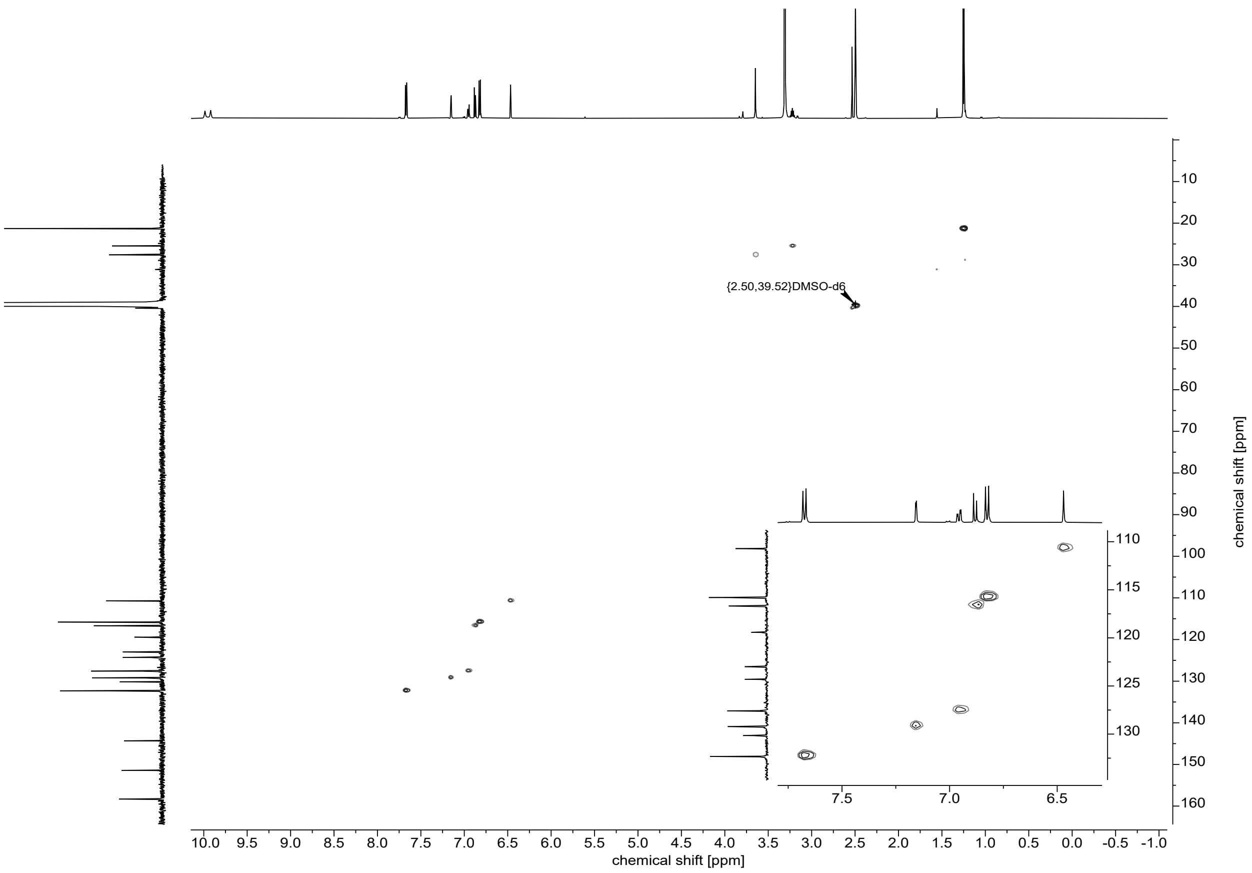

Figure S54. ^{13}C-HSQC NMR spectrum (600 MHz) of *Z*-**6** in DMSO-*d*$_6$. Inset shows expanded region of the spectrum from δ_C 110-135 ppm and δ_H 6.3-7.8 ppm.

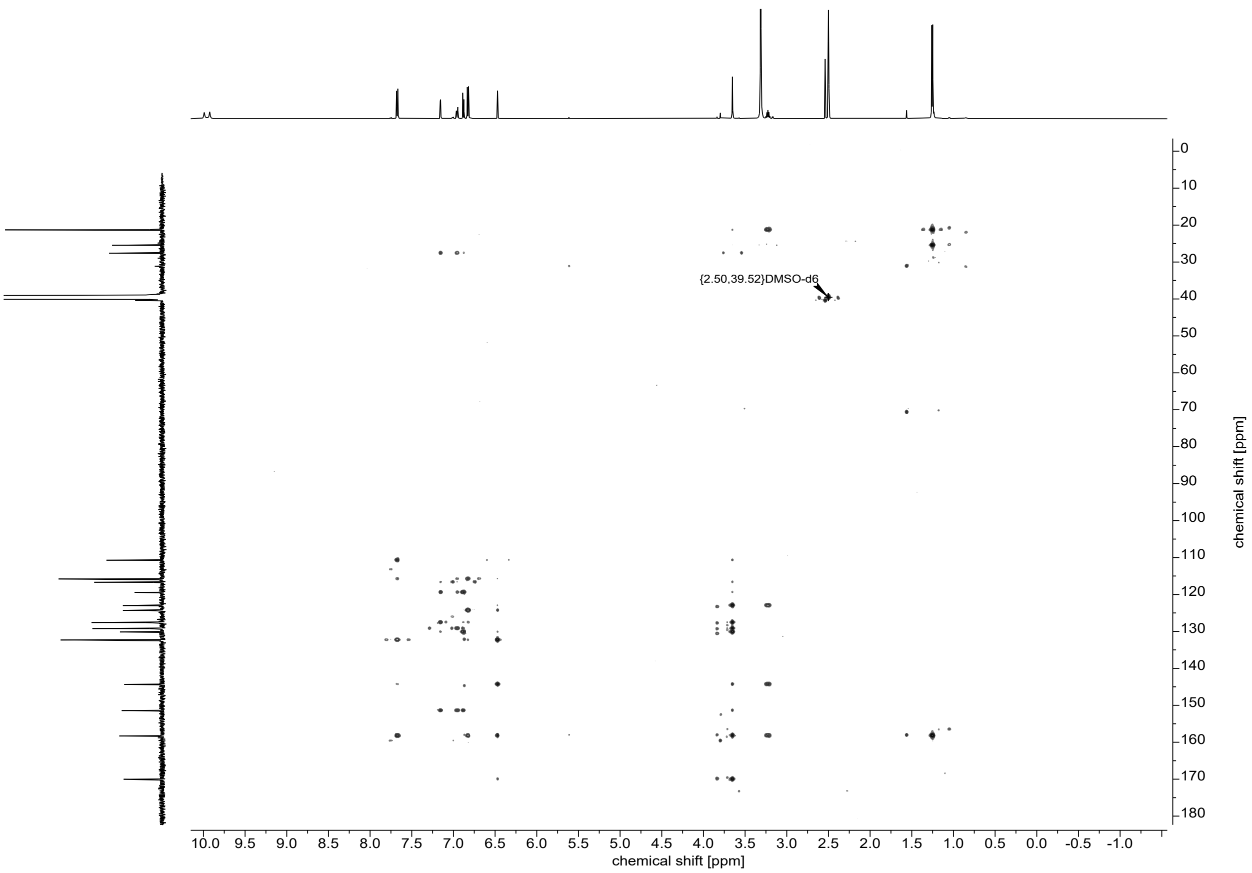

Figure S55. ^{13}C-HMBC NMR spectrum (600 MHz) of *Z*-**6** in DMSO-d_6.

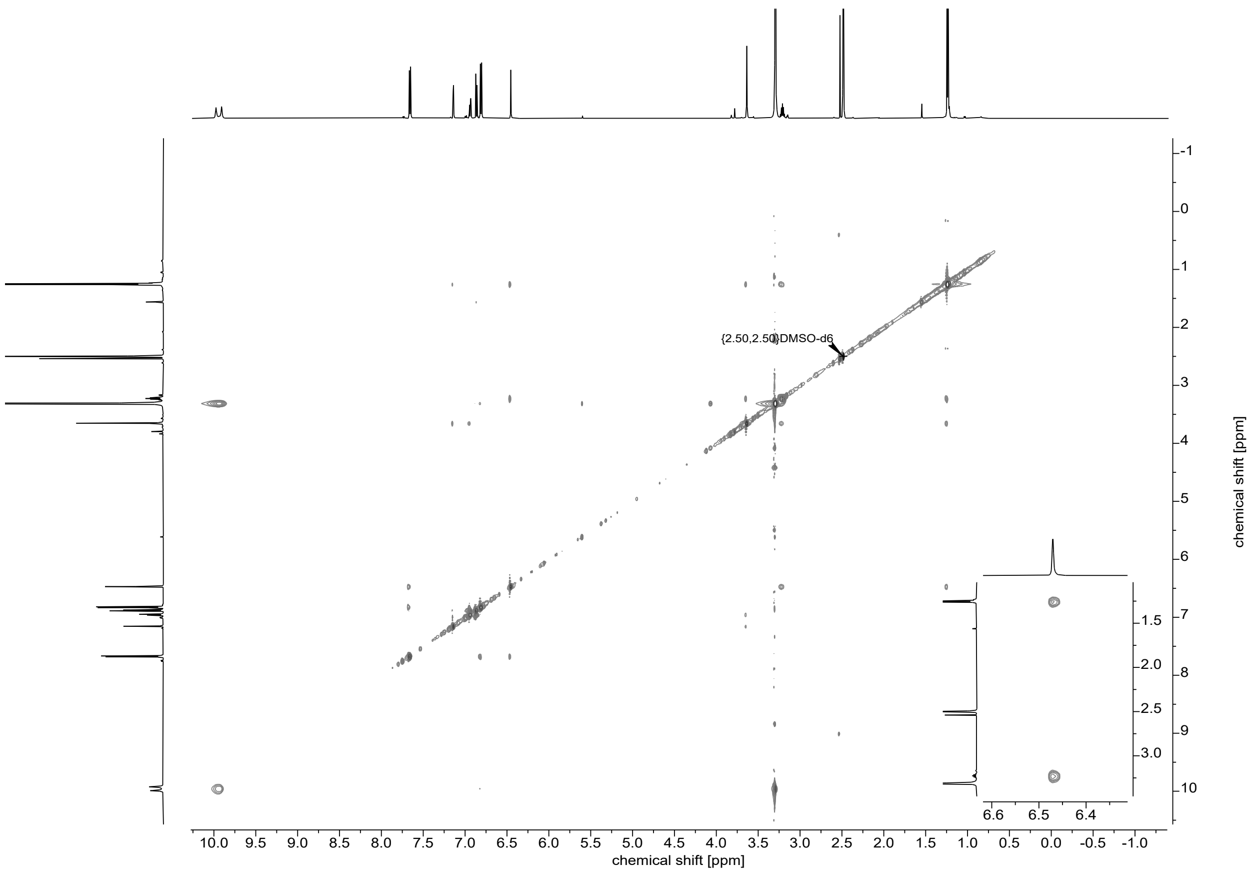

Figure S56. ROESY NMR spectrum (600 MHz) of *Z*-**6** in DMSO-d_6. Inset shows expanded region of the spectrum from δ_H 1.25-4.0 ppm and δ_H 6.3-6.6 ppm, highlighting representative NOE correlations hat confirm the presence of Z isomer.

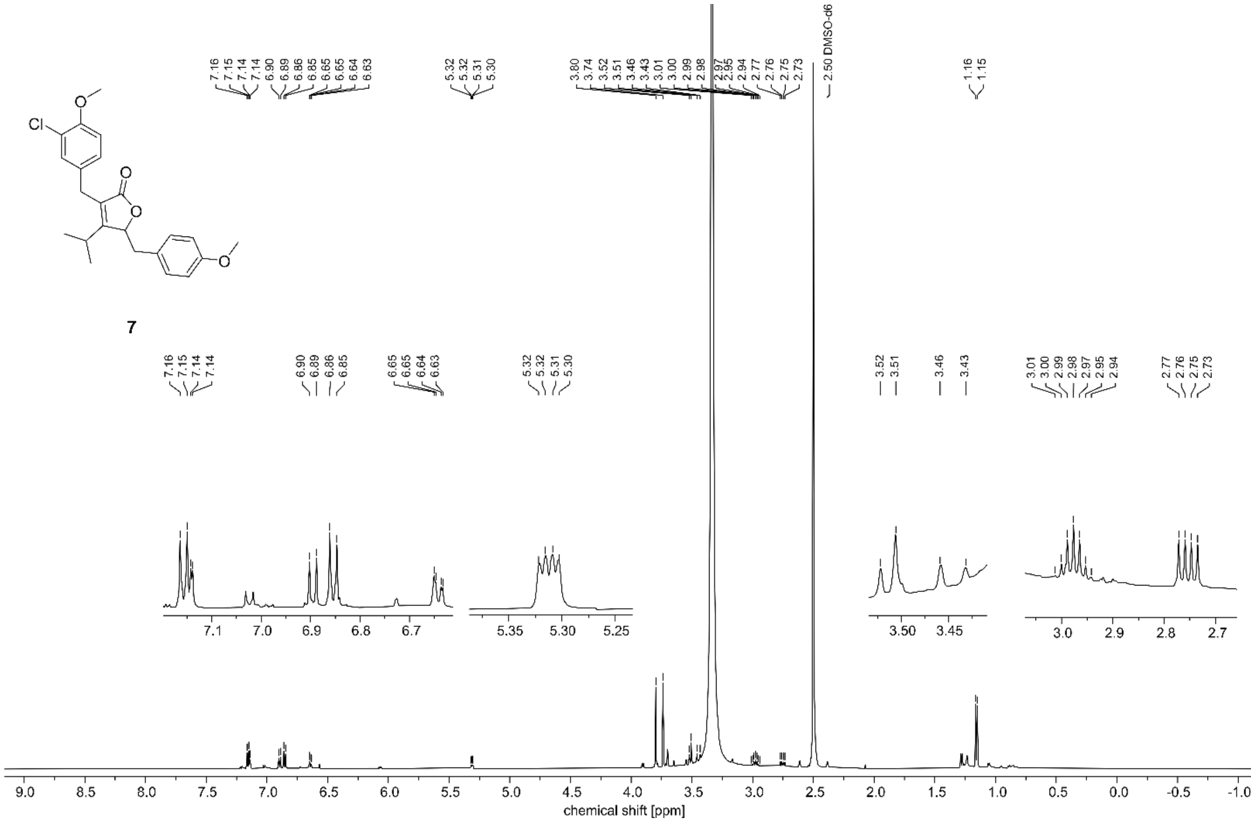

Figure S57. ^{1}H NMR spectrum (600 MHz) of **7** in DMSO-d_6. Insets show expanded regions of the spectrum from δ_H 6.6-7.2 ppm, δ_H 3.25-5.4 ppm, δ_H 3.40-3.55 ppm, and δ_H 2.7-3.0 ppm. One proton signal at δ_H 3.29 ppm is obscured by the residual water signal in the ^{1}H NMR spectrum, but its presence is confirmed by its correlation in the ^{13}C-HMQC NMR spectrum.

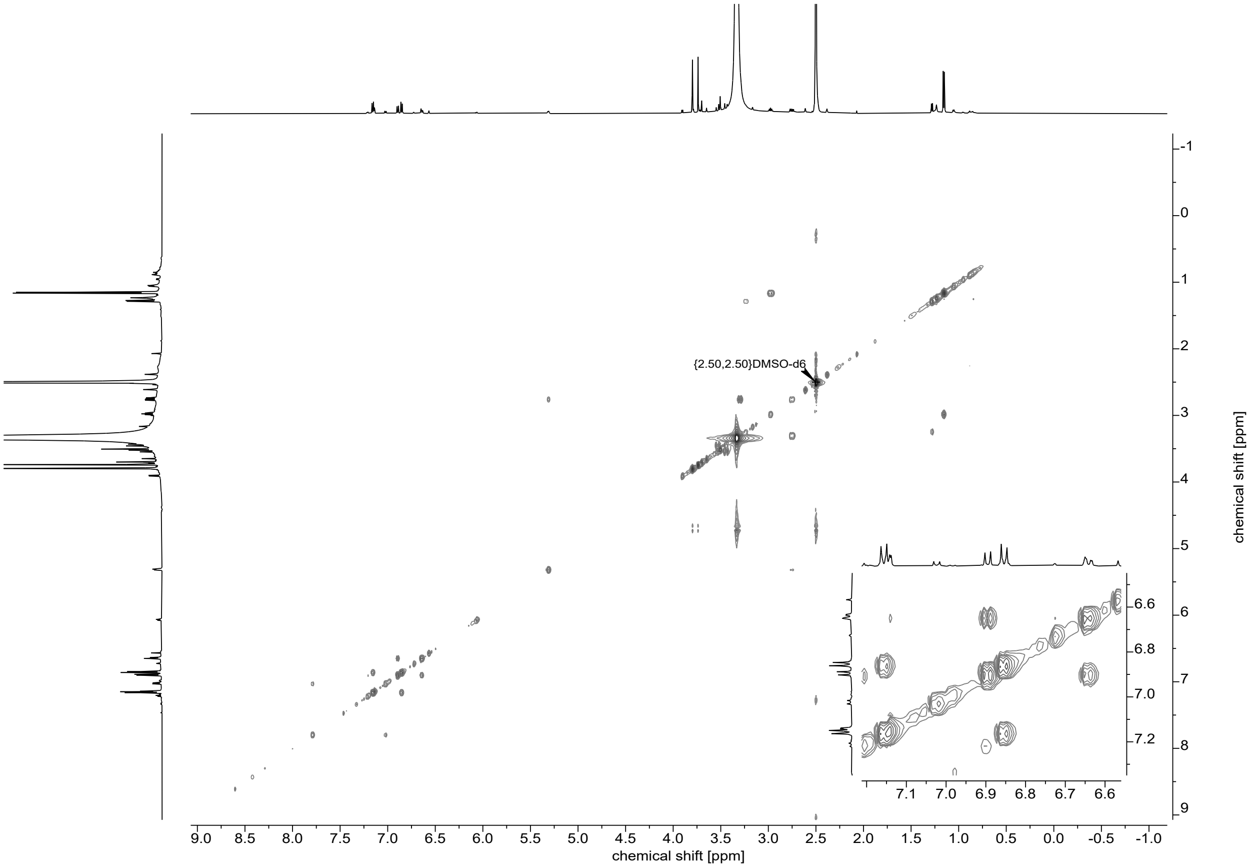

Figure S58. COSY NMR spectrum (600 MHz) of **7** in DMSO-d_6. Inset shows expanded region of the spectrum from δ_H 6.5-7.3 ppm, and δ_H 6.6-7.2 ppm.

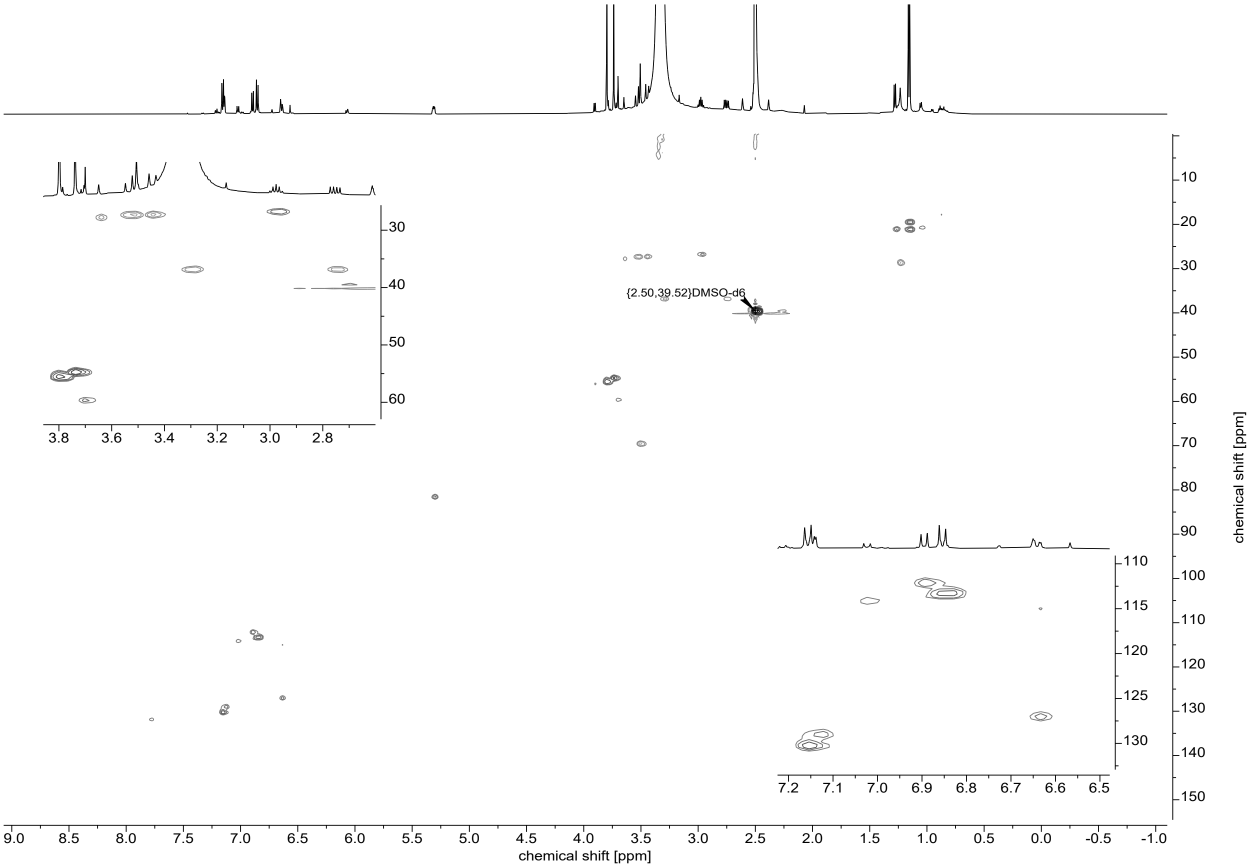

Figure S59. ^{13}C-HMQC NMR spectrum (600 MHz) of **7** in DMSO-*d*$_6$. Insets show expanded regions of the spectrum from δ_H 2.7-3.8 ppm and δ_C 25-65 ppm, and δ_H 6.5-7.2 ppm, and δ_C 110-135 ppm. One proton signal at δ_H 3.29 ppm, obscured by the residual water signal in the ^{1}H NMR spectrum, its presence is confirmed by its correlation in here in the ^{13}C-HMQC NMR spectrum.

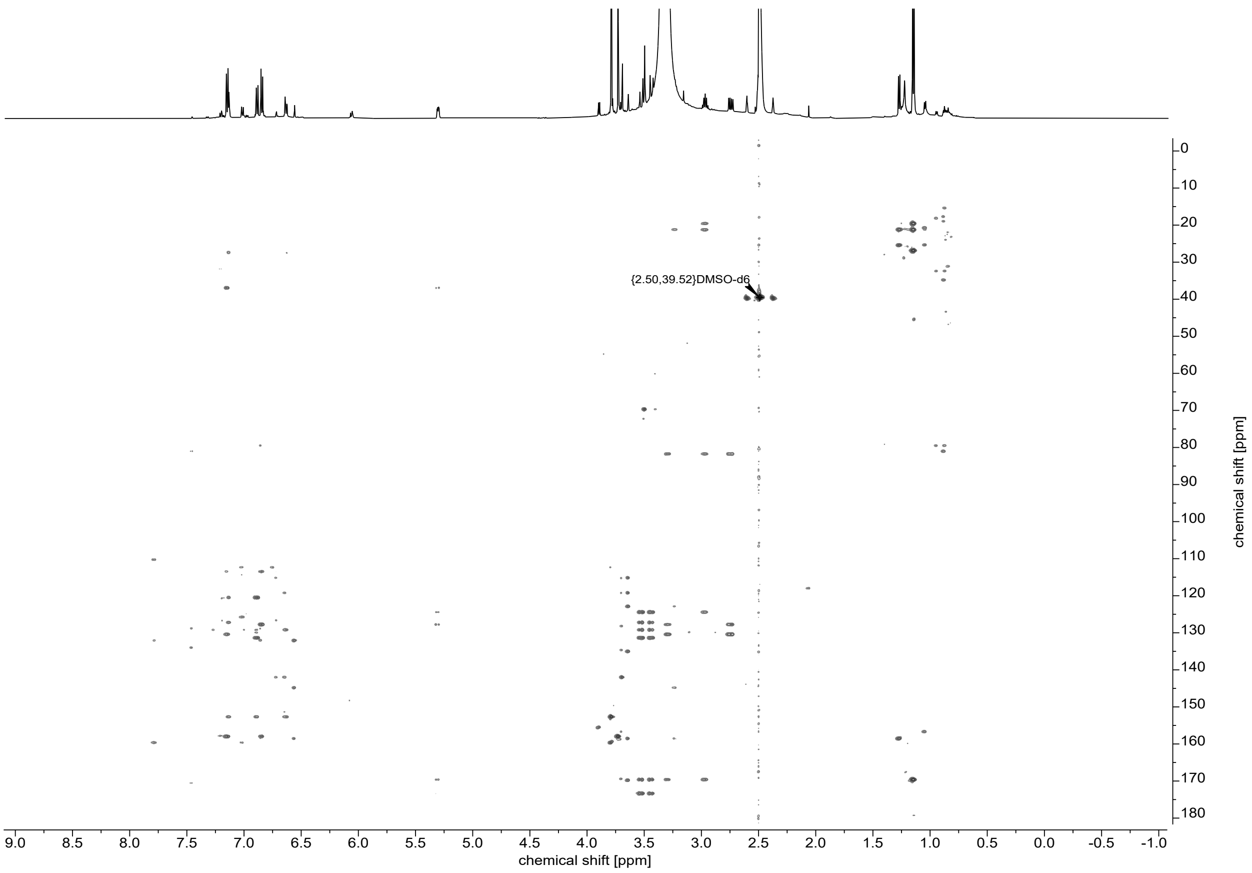

Figure S60. ^{13}C-HMBC NMR spectrum (600 MHz) of **7** in DMSO-d_6.

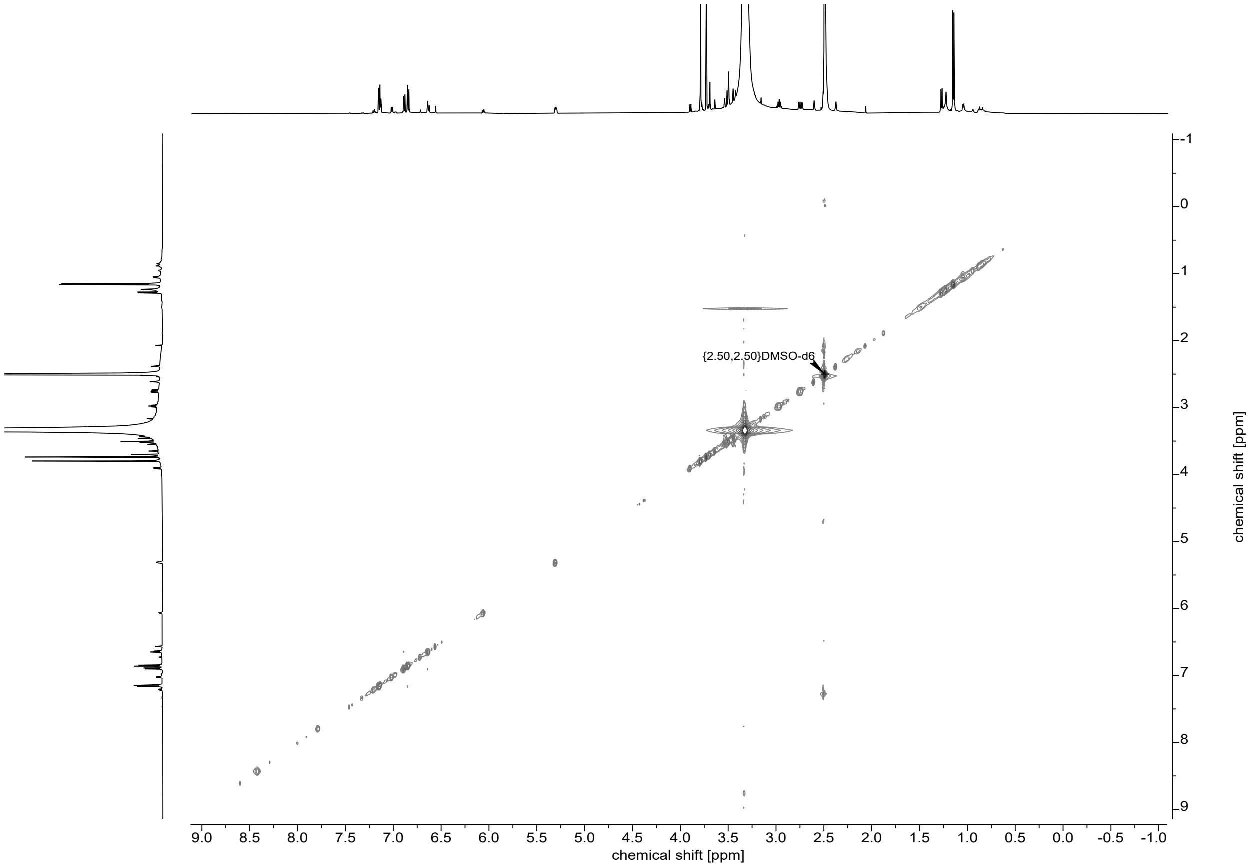

Figure S61. NOESY NMR spectrum (600 MHz) of **7** in DMSO-d_6.

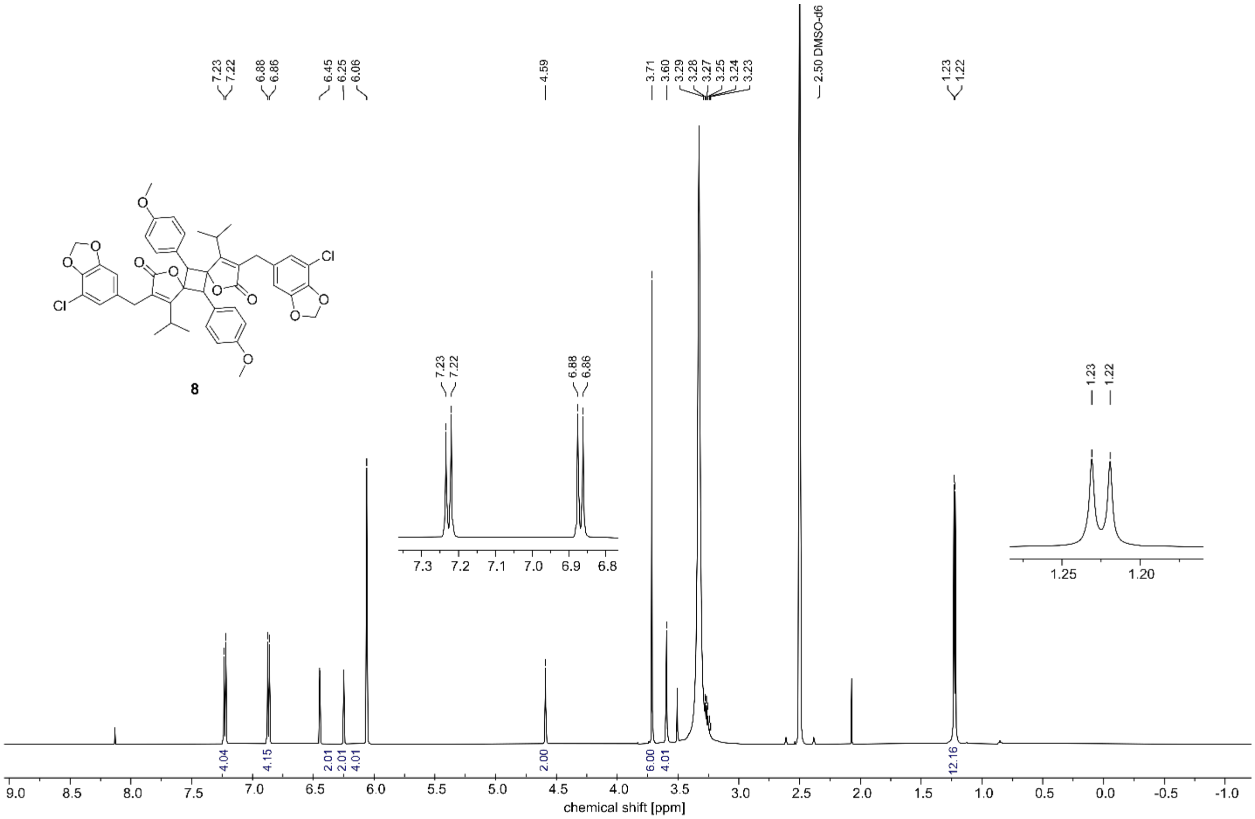

Figure S62. ^{1}H NMR spectrum (600 MHz) of **8** in DMSO-d_6.

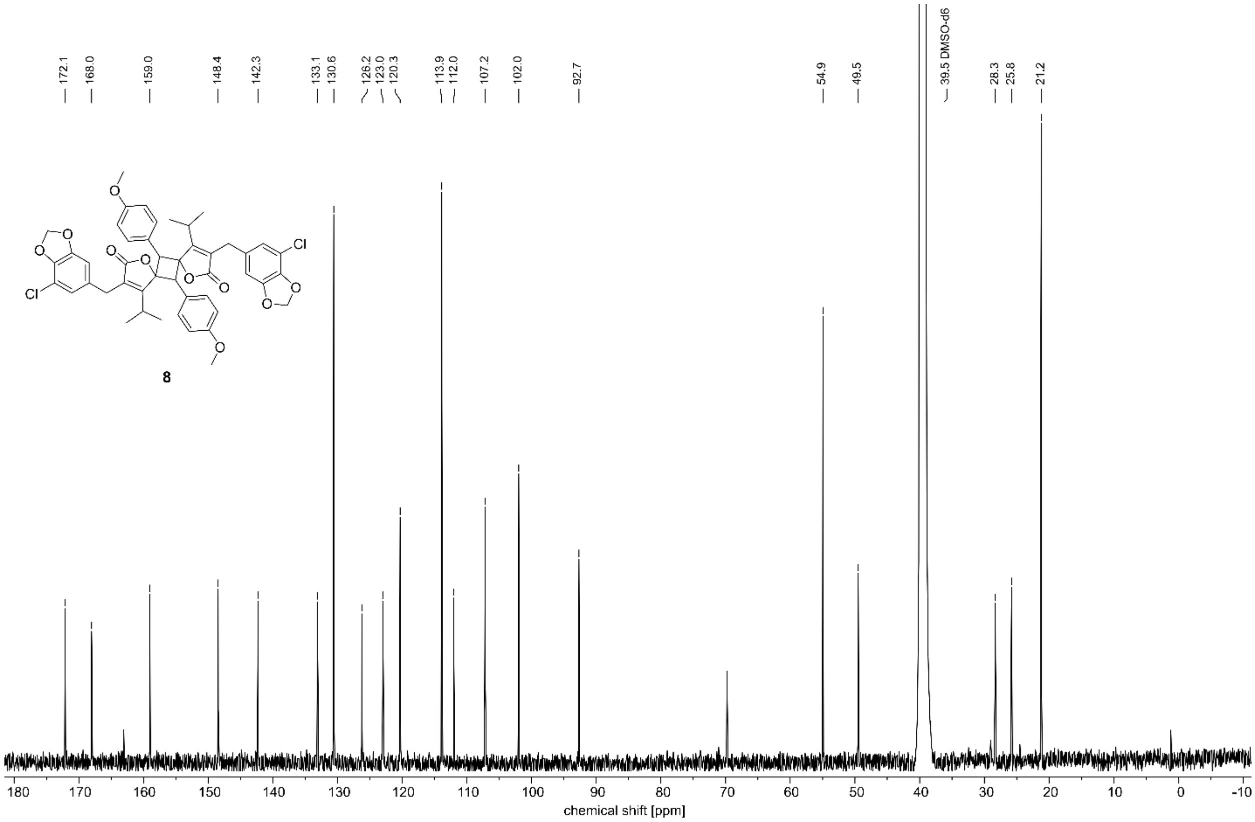

Figure S63. ^{13}C NMR spectrum (150 MHz) of **8** in DMSO-d_6.

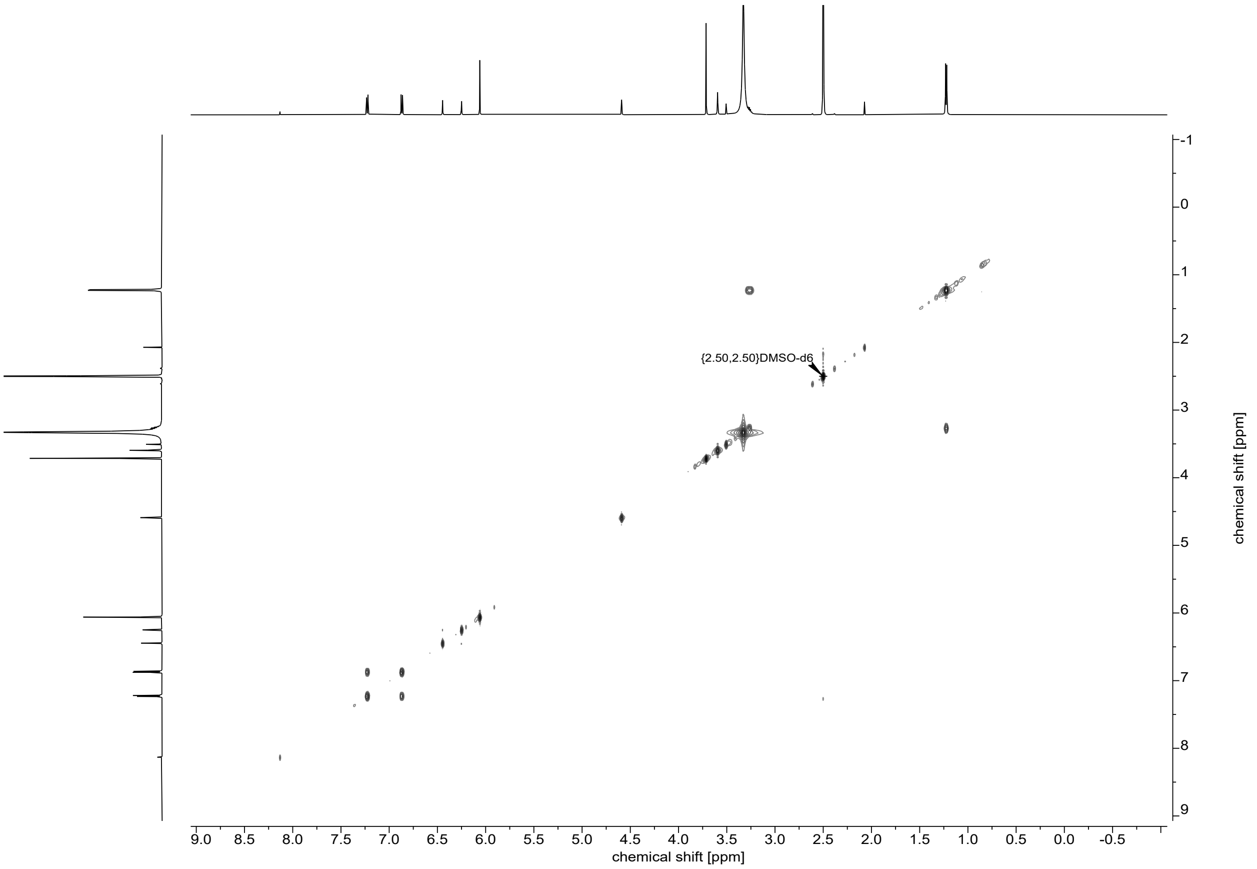

Figure S64. COSY NMR spectrum (600 MHz) of **8** in DMSO-d_6.

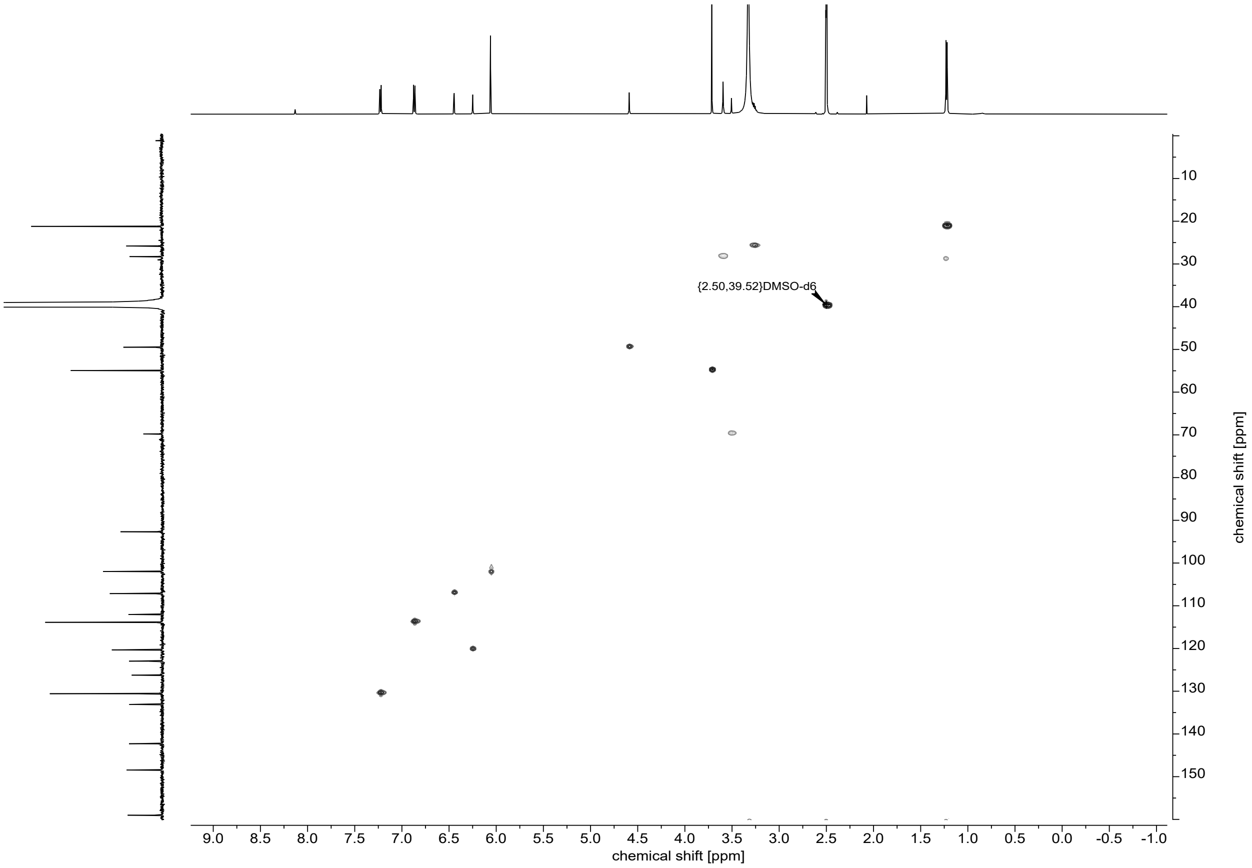

Figure S65. ^{13}C-HSQC NMR spectrum (600 MHz) of **8** in DMSO-d_6.

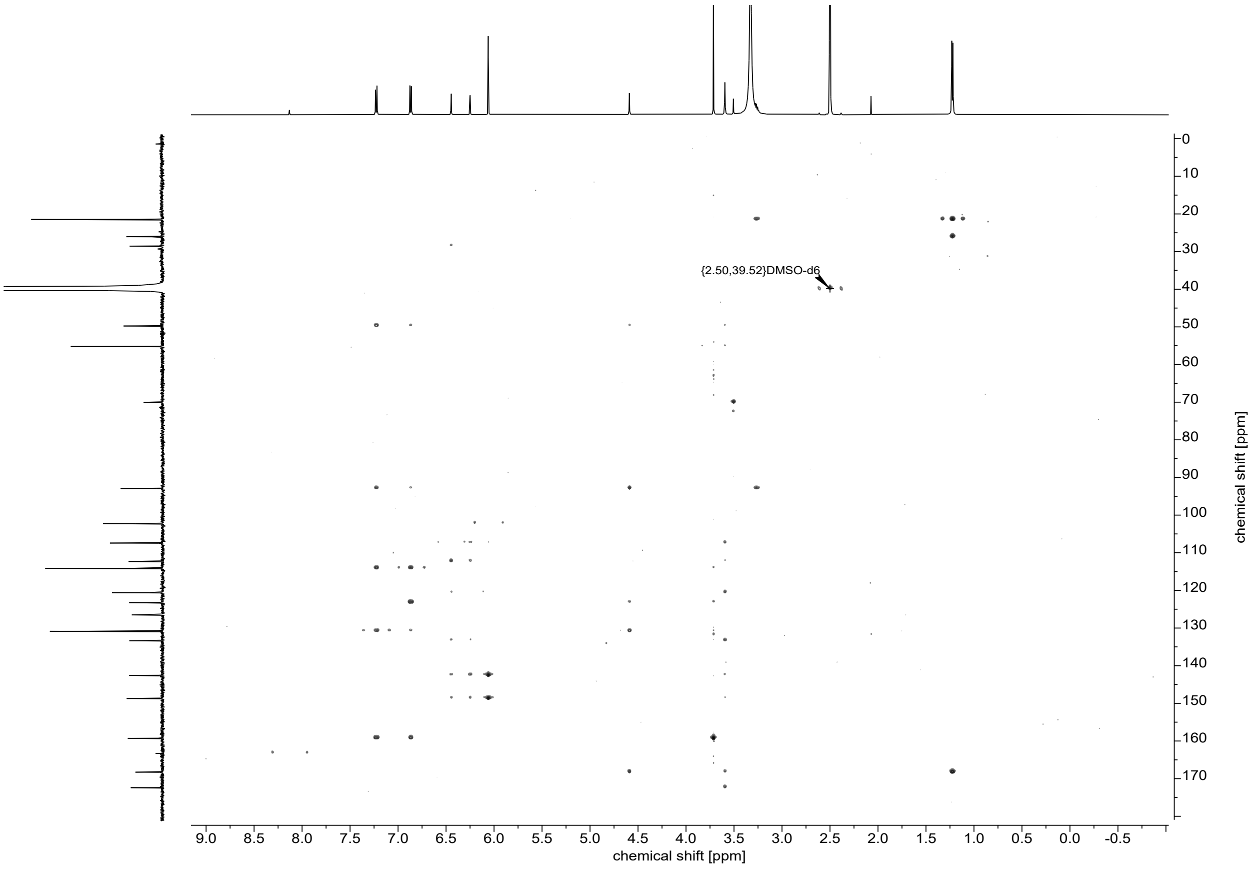

Figure S66. ^{13}C-HMBC NMR spectrum (600 MHz) of **8** in DMSO-d_6.

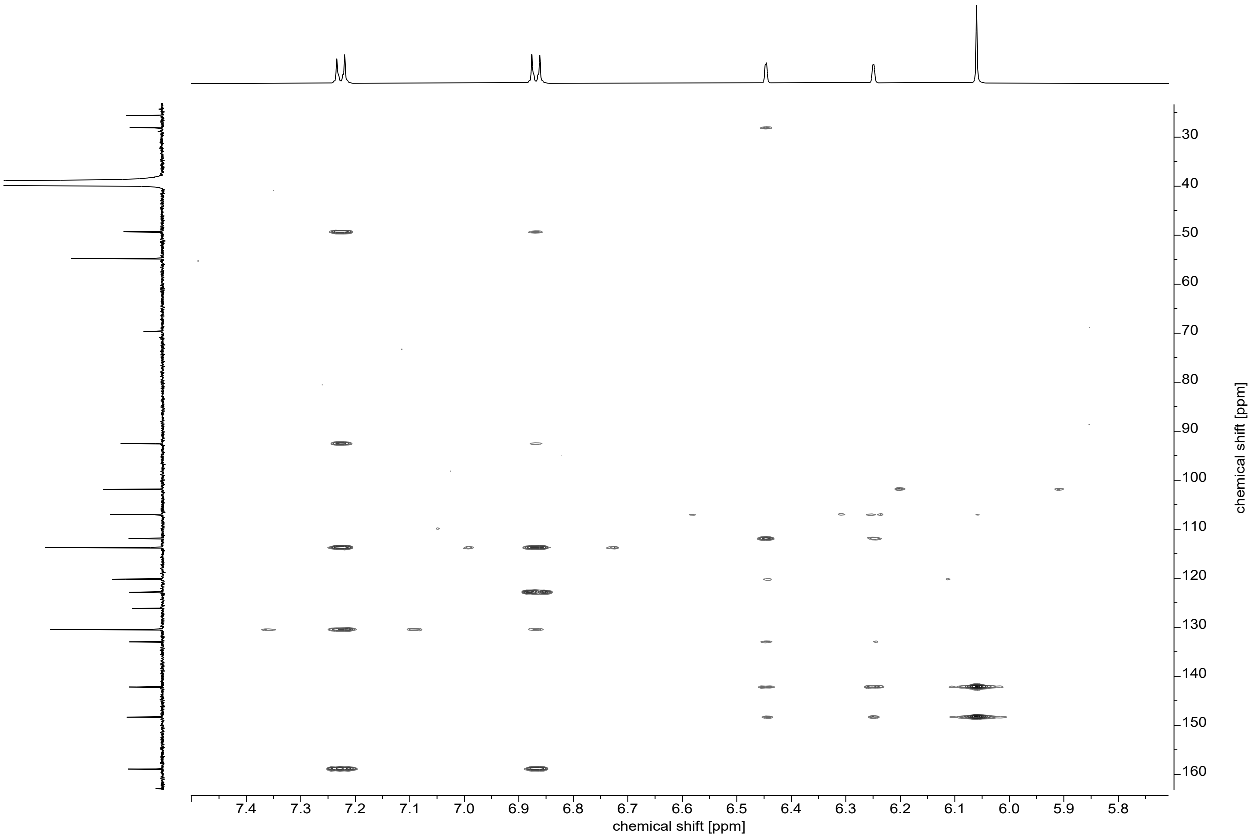

Figure S67. ^{13}C-HMBC NMR spectrum (600 MHz) of **8** in DMSO-d_6. Zoomed in the δ_H 5.75–7.5 ppm region.

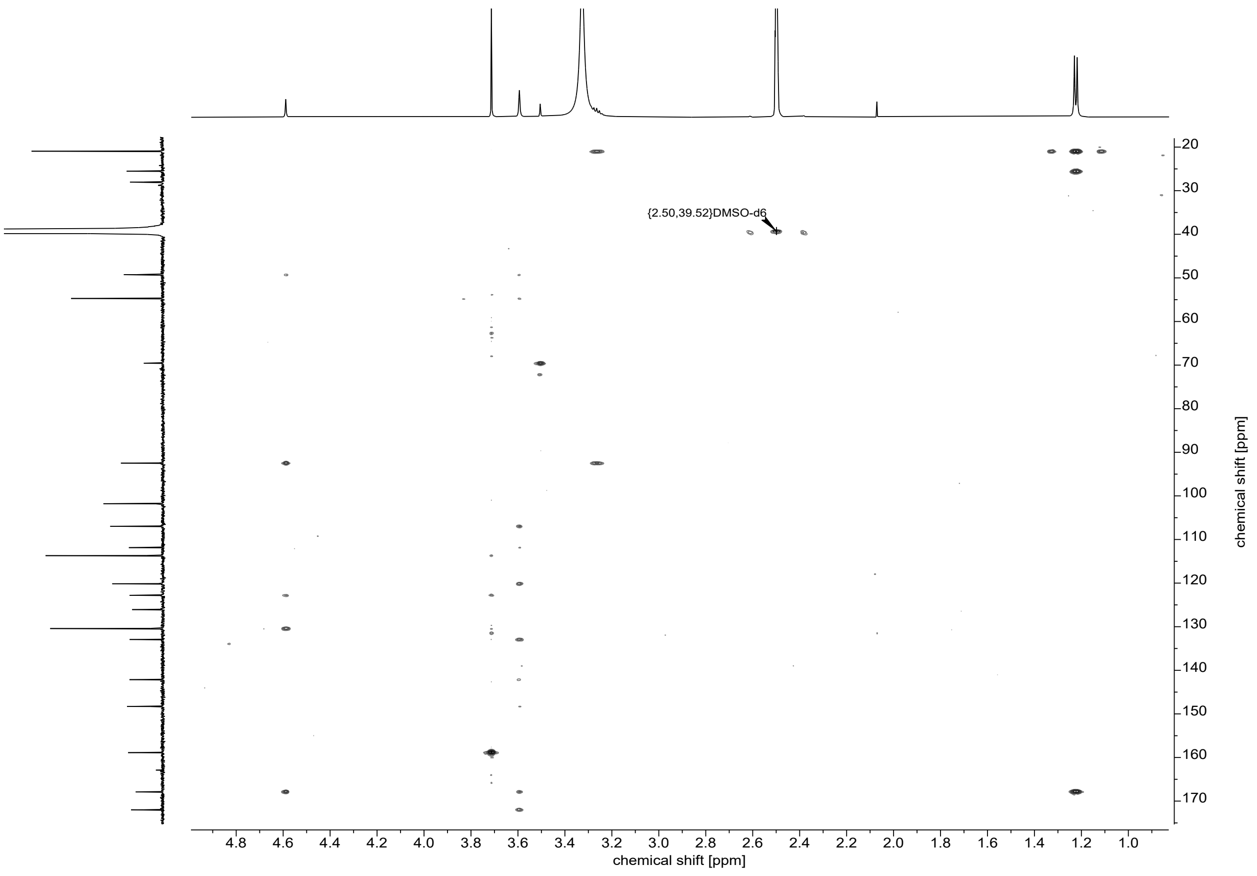

Figure S68. ^{13}C-HMBC NMR spectrum (600 MHz) of **8** in DMSO-d_6. Zoomed in the δ_H 0–4.9 ppm region.

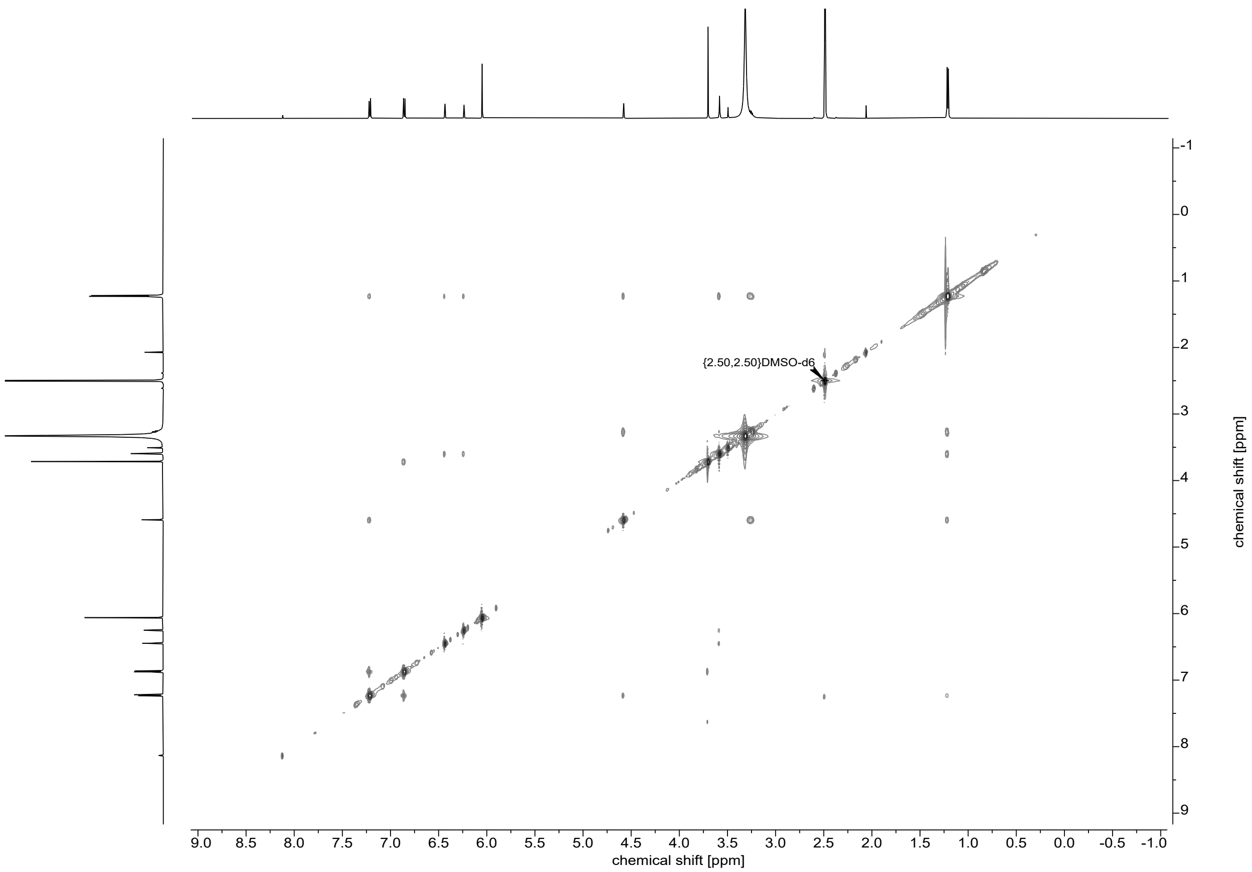

Figure S69. NOESY NMR spectrum (600 MHz) of **8** in DMSO-d_6.

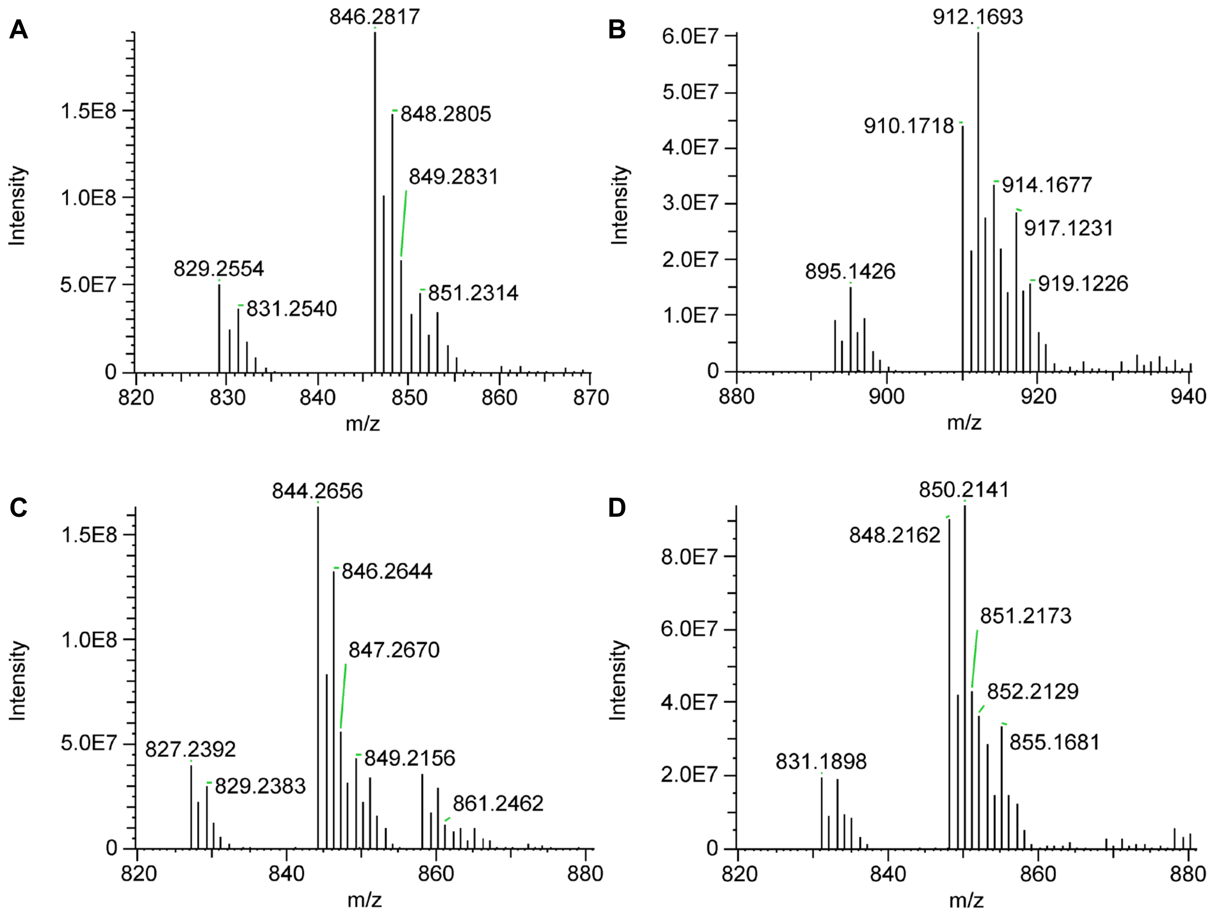

Figure S70. HRMS[1] spectra of additional dimers identified during targeted re-examination of the *Tolypothrix* sp. PCC9009 biomass extract, ESI pos. mode. **A** Derived from two monomers of **3** ($[M+H]^+$ *m/z* 829.2554, $C_{46}H_{47}O_{10}Cl_1$, calc. 829.2541, Δ 1.3 ppm). **B** Derived from two monomers of **5** ($[M+H]^+$ at *m/z* 893.1443, $C_{46}H_{41}O_{10}Cl_4$, calc. 893.1448, Δ 0.6 ppm). **C** Composed of two monomers that have not yet been characterized ($[M+H]^+$ *m/z* 827.2392, $C_{46}H_{45}O_{10}Cl_2$, calc. 827.2384, Δ 1.0 ppm). **D** Composed of two monomers that have not yet been characterized ($[M+H]^+$ *m/z* 831.1898, $C_{45}H_{42}O_9Cl_3$, calc. 831.1889, Δ 1.1 ppm).

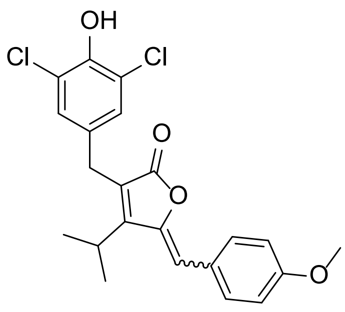

(*E/Z*)-**A**

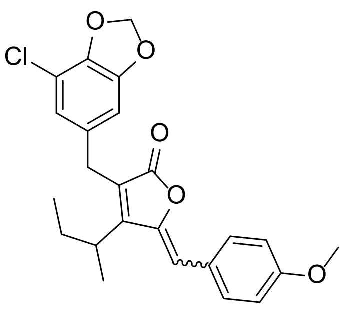

(*E/Z*)-**B**

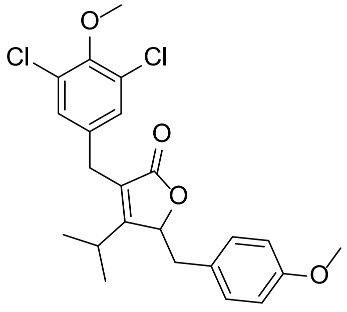

C

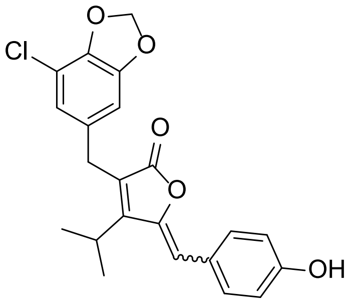

(*E/Z*)-**D**

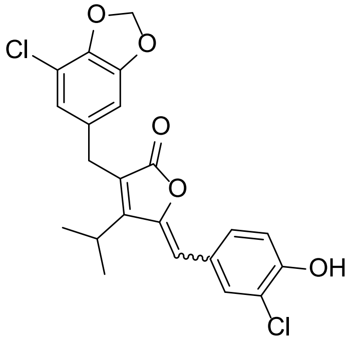

(*E/Z*)-**E**

Figure S71. Proposed structures of compounds **A** to **E** based on HRMS^2 data. Proposals were generated based on an in-depth FBMN analysis, comparative evaluation with NMR-elucidated congeners, and biosynthetic feasibility.

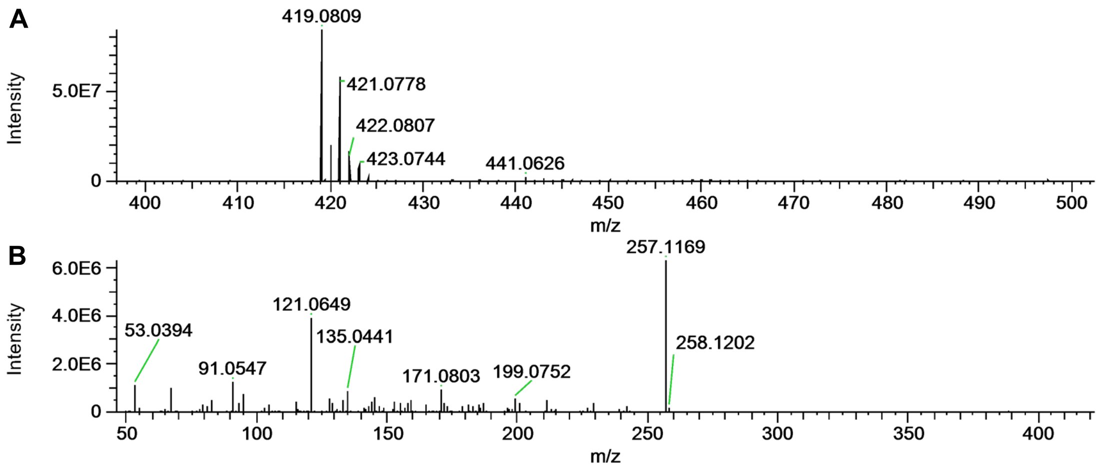

Figure S72. HRMS data of (*E*/*Z*)-**A**. **A** The HRMS[1] spectrum (ESI pos. mode, *m/z* 419.0809) showing the characteristic isotope pattern. **B** HRMS[2] spectrum (precursor ion *m/z* 419.0809), ESI pos. mode, collision energy 55 eV.

Table S8. Annotation of key ions observed in the HRMS[2] spectrum of (*E*/*Z*)-**A**.

m/z	mol. formula	proposed structure
257.1169	$C_{16}H_{17}O_3^+$ (Δ 0.4 ppm)	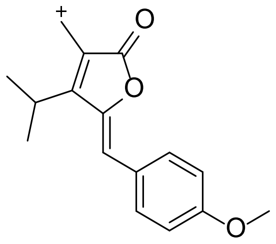
174.9710	$C_7H_5Cl_2O^+$ (Δ 1.1 ppm)	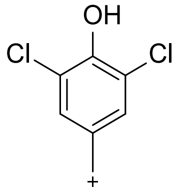
121.0649	$C_8H_9O^+$ (Δ 0.8 ppm)	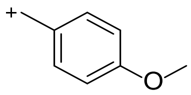

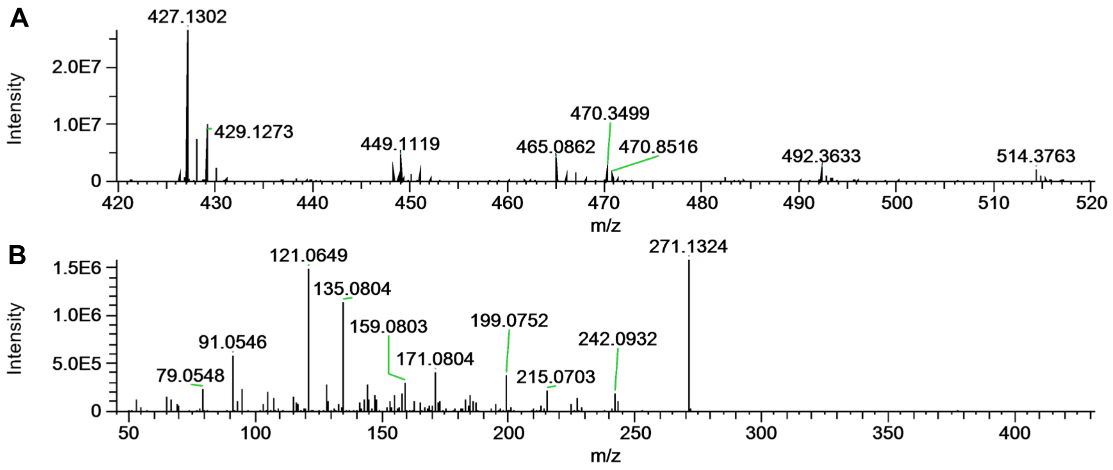

Figure S73. HRMS data of (*E*/*Z*)-**B** (ESI pos. mode). **A** $HRMS^1$ spectrum (*m/z* 427.1302) showing characteristic isotope pattern. **B** $HRMS^2$ spectrum (precursor ion *m/z* 427.1302), ESI pos. mode, collision energy 55 eV.

Table S9. Annotation of key ions observed in the $HRMS^2$ spectrum of (*E*/*Z*)-**B**.

m/z	mol. formula	proposed structure
271.1324	$C_{17}H_{19}O_3^+$ (Δ 1.8 ppm)	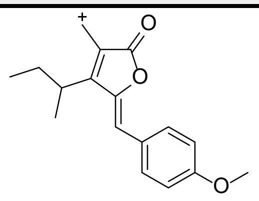
229.0859	$C_{14}H_{13}O_3^+$ (Δ 0.0 ppm)	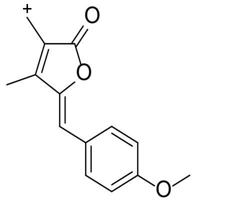
215.0703	$C_{13}H_{11}O_3^+$ (Δ 0.0 ppm)	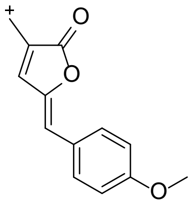
135.0804	$C_9H_{11}O^+$ (Δ 0.0 ppm)	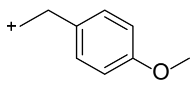

121.0649	$C_{17}H_{19}O_3^+$ (Δ 0.8 ppm)	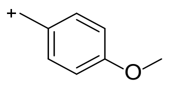

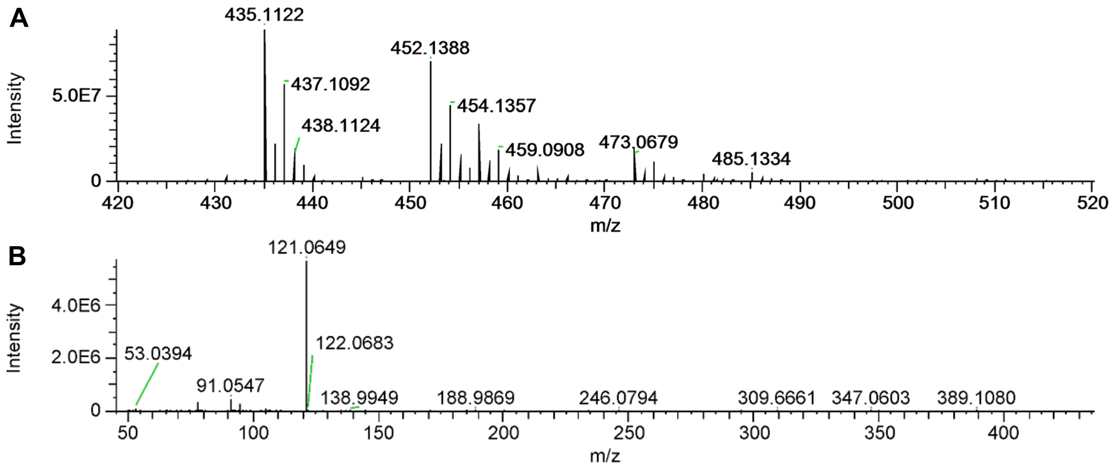

Figure S74. HRMS data of **C**. **A** The $HRMS^1$ spectrum (ESI pos. mode, *m/z* 435.1122) showing the characteristic isotope pattern. **B** $HRMS^2$ spectrum (precursor ion *m/z* 435.1122), ESI pos. mode, collision energy 55 eV.

Table S10. Annotation of key ions observed in the $HRMS^2$ spectrum of **C**.

m/z	mol. formula	proposed structure
188.9869	$C_7H_5Cl_2O^+$ (Δ 0.5 ppm)	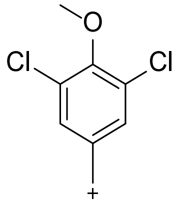
121.0649	$C_8H_9O^+$ (Δ 0.8 ppm)	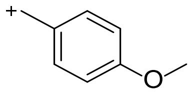

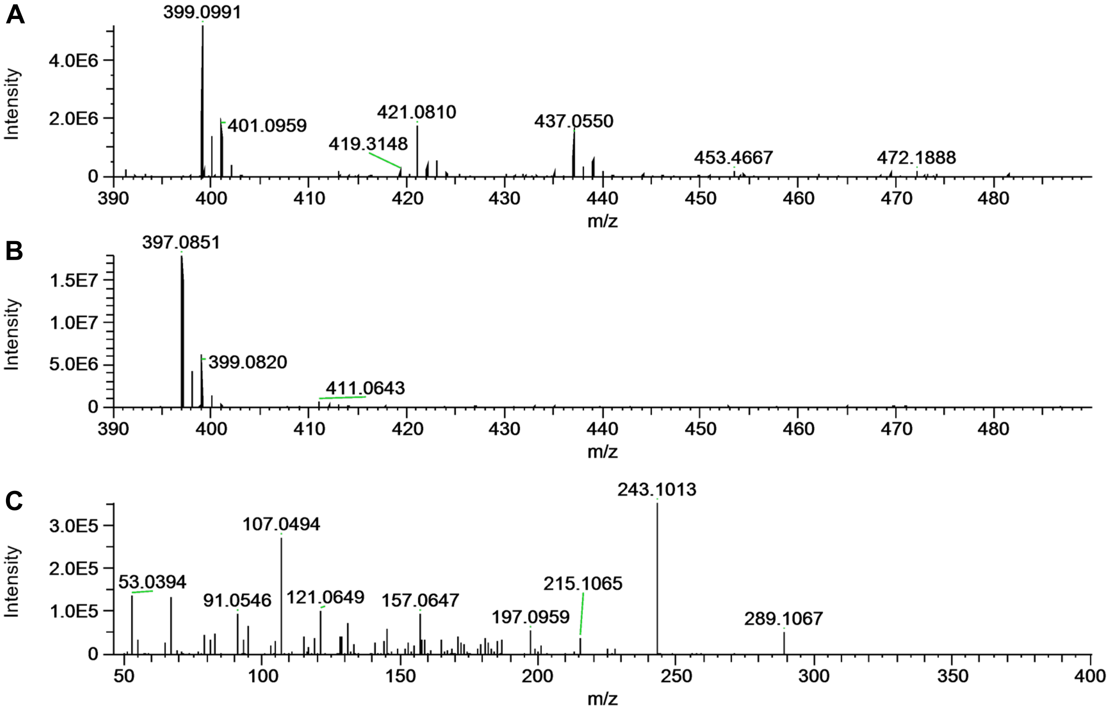

Figure S75. HRMS data of (*E*/*Z*)-**D**. **A** HRMS[1] spectrum (ESI pos. mode, *m/z* 399.0991), characteristic isotope pattern. **B** The HRMS[1] spectrum (ESI neg. mode, *m/z* 397.0851), characteristic isotope pattern. **C** HRMS[2] spectrum (precursor ion *m/z* 399.0991), ESI pos. mode, CE 55 eV.

Table S11. Annotation of key ions observed in the HRMS[2] spectrum of (*E*/*Z*)-**D**.

m/z	mol. formula	proposed structure
243.1013	$C_{15}H_{15}O_3^+$ (Δ 1.2 ppm)	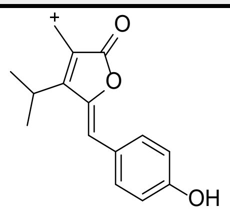
169.0051	$C_8H_6ClO_2^+$ (Δ 0.0 ppm)	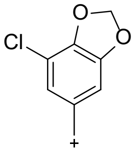
107.0494	$C_8H_6ClO^+$ (Δ 2.8 ppm)	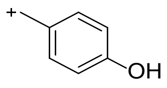

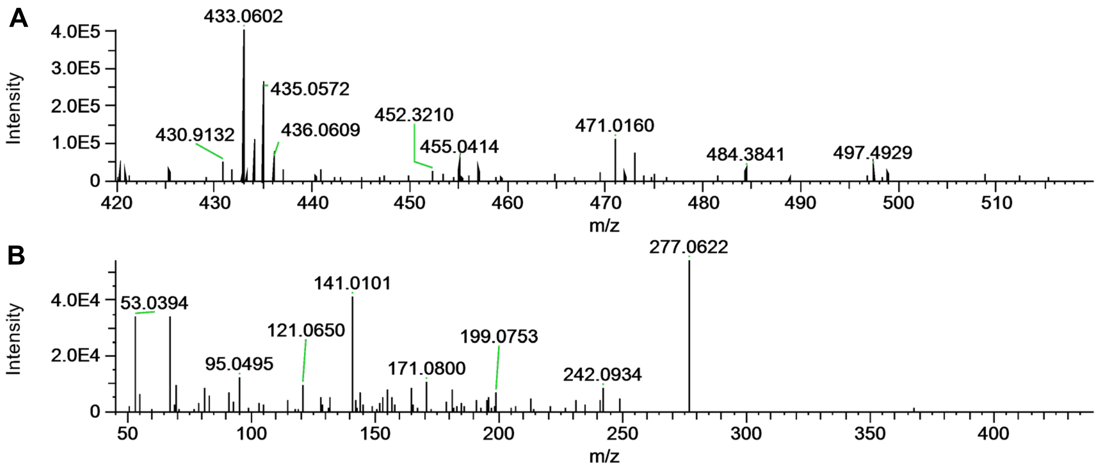

Figure S76. HRMS data of (*E*/*Z*)-**E**. **A** The HRMS[1] spectrum (ESI pos. mode, *m/z* 435.1122) showing the characteristic isotope pattern. **B** HRMS[2] spectrum (precursor ion *m/z* 435.1122), ESI pos. mode, collision energy 55 eV.

Table S12. Annotation of key ions observed in the HRMS[2] spectrum of (*E*/*Z*)-**E**.

m/z	mol. formula	proposed structure
277.0622	$C_{15}H_{14}ClO_3^+$ (Δ 1.4 ppm)	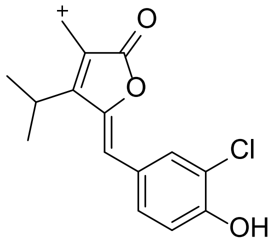
153.0102	$C_8H_6ClO^+$ (Δ 0.0 ppm)	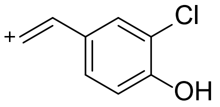
141.0101	$C_7H_6ClO^+$ (Δ 0.7 ppm)	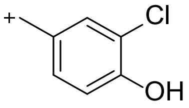

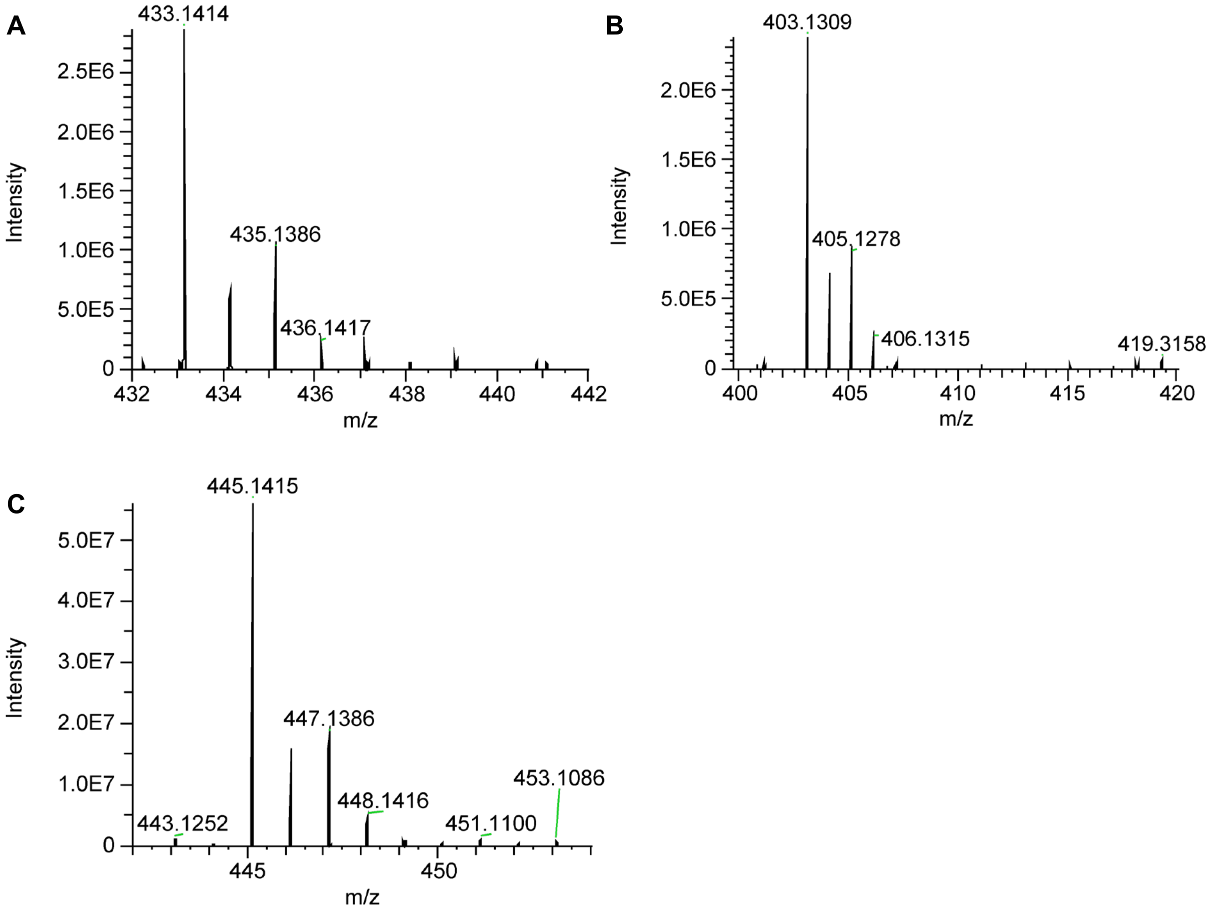

Figure S77. HRMS[1] spectra of corresponding putatively 3-hydroxy derivatives for: **A** compound **3** ($[M+H]^+$ *m/z* 433.1414, $C_{23}H_{26}O_6Cl$, calc. 433.1412, Δ 0.5 ppm). **B** Compound **2** ($[M+H]^+$ *m/z* 403.1309, $C_{22}H_{24}O_5Cl$, calc. 403.1307, Δ 0.5 ppm). **C** Compound **B** ($[M+H]^+$ *m/z* 445.1413, $C_{24}H_{26}O_6Cl$, calc. 445.1412, Δ 0.7 ppm).

Table S13. Sulforhodamine B Cytotoxicity Assay. Assay performed against HCT116 human colon carcinoma cells in three independent biological replicates. Mean absorbance values normalized to the solvent control (0.1% DMSO). SD: standard deviation (1X).

**Treatment**	**4**		**1**		**7**	
**c** [**µM**]	mean [%]	± SD [%]	mean [%]	± SD [%]	mean [%]	± SD [%]
**0.1**	101.5	11.3	102.8	10.5	99.3	12.8
**1**	102.5	8.9	100.0	10.5	99.6	12.8
**10**	95.5	10.5	91.9	8.4	97.5	12.0
**25**	91.5	9.4	79.3	12.8	93.1	10.4
**50**	83.1	8.9	72.9	12.6	79.2	10.6
